# Duplication of a *Pks* gene cluster and subsequent functional diversification facilitate environmental adaptation in *Metarhizium* species

**DOI:** 10.1371/journal.pgen.1007472

**Published:** 2018-06-29

**Authors:** Guohong Zeng, Peng Zhang, Qiangqiang Zhang, Hong Zhao, Zixin Li, Xing Zhang, Chengshu Wang, Wen-Bing Yin, Weiguo Fang

**Affiliations:** 1 Institute of Microbiology, Zhejiang University, Hangzhou, China; 2 State Key Laboratory of Mycology, Institute of Microbiology, Chinese Academy of Sciences, Beijing, People’s Republic of China; 3 CAS Key Laboratory of Insect Developmental and Evolutionary Biology, Shanghai Institute of Plant Physiology and Ecology, Chinese Academy of Sciences, Shanghai, China; Vanderbilt University, UNITED STATES

## Abstract

The ecological importance of the duplication and diversification of gene clusters that synthesize secondary metabolites in fungi remains poorly understood. Here, we demonstrated that the duplication and subsequent diversification of a gene cluster produced two polyketide synthase gene clusters in the cosmopolitan fungal genus *Metarhizium*. Diversification occurred in the promoter regions and the exon-intron structures of the two *Pks* paralogs (*Pks1* and *Pks2*). These two *Pks* genes have distinct expression patterns, with *Pks1* highly expressed during conidiation and *Pks2* highly expressed during infection. Different upstream signaling pathways were found to regulate the two *Pks* genes. *Pks1* is positively regulated by Hog1-MAPK, Slt2-MAPK and Mr-OPY2, while *Pks2* is positively regulated by Fus3-MAPK and negatively regulated by Mr-OPY2. *Pks1* and *Pks2* have been subjected to positive selection and synthesize different secondary metabolites. PKS1 is involved in synthesis of an anthraquinone derivative, and contributes to conidial pigmentation, which plays an important role in fungal tolerance to UV radiation and extreme temperatures. Disruption of the *Pks2* gene delayed formation of infectious structures and increased the time taken to kill insects, indicating that *Pks2* contributes to pathogenesis. Thus, the duplication of a *Pks* gene cluster and its subsequent functional diversification has increased the adaptive flexibility of *Metarhizium* species.

## Author summary

In fungi, gene clusters that synthesize secondary metabolites are hotspots for the generation of fungal metabolic diversity through gene duplication, but their ecological importance remains poorly understood. *Metarhizium* species are adapted to life as insect pathogens, plant symbionts and saprophytes, enabling the function of individual genes to be studied in diverse fungal lifestyles. We discovered that a duplication of a *Pks* (polyketide synthase) gene cluster in *Metarhizium* species has facilitated its ecological opportunism. Sequence diversifications occurred in the promoter regions, the intro-exon structures, and the coding sequences of the two *Pks* paralogs, and they synthesize different secondary metabolites, have different expression patterns, and are regulated by different signaling pathways. PKS1s involved in synthesis of conidial pigments and tolerance to several abiotic stresses. The *Pks2* gene is involved in formation of infectious structures (appressoria), enabling these fungi to kill insects faster. This *Pks* gene cluster duplication event may have been important for the adaptation of *Metarhizium* species to diverse environments.

## Introduction

Metabolic gene clusters are hotspots for the generation of fungal metabolic diversity through gene duplication, but the ecological importance of these gene clusters remains poorly understood [1]. Gene clusters that biosynthesize secondary metabolites (SMs) are particularly challenging, because they are often lineage-specific and their enzymatic activities are often poorly characterized [1]. Type I polyketides are common in fungi; they are usually synthesized by gene clusters that include polyketide synthase (*Pks*) genes [2, 3]. Fungi often have multiple *Pks* gene clusters as a result of gene duplication (typically) and horizontal gene transfer (less often) [1, 4–6]. After gene duplication, further diversification of *Pks* gene clusters might occur via lineage-specific duplication and loss events, or via functional divergences in response to ecological pressures [3, 4]. Functional analyses have shown that the SMs synthesized by some *Pks* gene clusters have important biological functions. For example, melanin allows some fungi to tolerate adverse environmental conditions, and allows other pathogenic fungi to infect hosts [7–9]. However, little is known about the relationship between the evolutionary diversification of *Pks* gene clusters and ecological adaptation in fungi.

The ascomycete genus *Metarhizium* is found worldwide, from the arctic to the tropics, and occupies an impressive array of environments including forests, savannahs, swamps, coastal zones, and deserts [10]. This worldwide distribution is largely attributed to the diverse lifestyles of *Metarhizium* species, and their tolerance to a broad range of environmental stresses including UV radiation and extreme temperatures [11–13]. *Metarhizium* has versatile lifestyles: it is a pathogen of arthropods, a saprophyte, and a colonizer of rhizosphere and plant roots [14]. The genomes of seven *Metarhizium* species (*Metarhizium robertsii*, *M*. *brunneum*, *M*. *anisopliae*, *M*. *guizhouense*, *M*. *majus*, *M*. *acridum*, and *M*. *album*) have previously been published [15]. A comparative genomic analyses of species in this genus indicated that host shift and speciation in *Metarhizium* were coupled with various evolutionary mechanisms including horizontal gene transfer and gene duplication. A significant relationship between SM-synthesizing gene clusters and infection structure (appressorium) formation suggested that the SMs produced by *Metarhizium* species might be pathogenicity factors [15]. The seven available *Metarhizium* genomes contain between 10 to 20 *Pks* genes, and in some species there is evidence of lineage-specific expansion [2, 15]. Few studies have focused on *Pks* genes in *Metarhizium*. To date, only *Mr-Pks1* (herein referred to as *Pks1*) and *Mr-Pks2* (herein referred to as *Pks2*) in a single species (*M*. *robertsii*) have been identified and investigated [16, 17]. Although it was shown that *Pks1* was involved in conidial pigmentation, the biological functions of *Pks2* have not been determined [16, 17]. The SMs synthesized by the PKS1 and PKS2 proteins also remain unidentified.

Here, we found that two *Pks* gene clusters in *Metarhizium* species were formed through the duplication of an ancient *Pks* gene cluster and following gene losses. Subsequent diversification in coding sequences, gene structures and promoter regions resulted in the two *Pks* paralogs (*Pks1* and *Pks2*). These paralogs have different biological functions: they have different expression patterns, and encode proteins that synthesize different SMs. We found that PKS1 is involved in synthesis of an anthraquinone derivative. *Pks2* is related to entomopathogenicity, while *Pks1* facilitates tolerance to UV radiation, and heat and cold stress.

## Results

### Two *Pks* gene clusters in *Metarhizium* species result from a gene cluster duplication

Using the PKS1 (MAA_07745) and PKS2 (MAA_03239) protein sequences in *M*. *robertsii* as queries, we performed a reciprocal BLASTP against the NCBI Fungal database (taxid: 4751). The best hit of PKS1 is different from that of PKS2 in each of five other *Metarhizium* species (*M*. *brunneum*, *M*. *anisopliae*, *M*. *guizhouense*, *M*. *majus*, and *M*. *acridum*) [15]. However, the best hit of PKS1 is the same as that of PKS2 in the basal *Metarhizium* species (*M*. *album*) and in the non-*Metarhizium* species. When the best hits of the *M*. *robertsii* PKS1 and PKS2 in *M*. *album* and the non-*Metarhizium* species were used as queries for reverse BLASTP against the *M*. *robertsii* protein database, the best hit was either PKS1 or PKS2. Based on this reciprocal BLASTP analysis, we speculated that PKS1 and PKS2 in *Metarhizium* species might result from gene duplication. To confirm this speculation, we performed phylogenetic analysis and predicted gene duplication with *Metarhizium*’s PKS1s and PKS2s, and their best hits (e-value cutoff 1e^-05^) from other Ascomycota species (S1 Table).

In *M*. *brunneum*, *M*. *anisopliae*, *M*. *guizhouense*, *M*. *majus*, and *M*. *robertsii*, the *Pks1* gene is adjacent to *EthD* [17]. In GenBank, however, the corresponding genomic region in *M*. *acridum* and *M*. *album* was annotated as a single gene encoding a protein containing all of EthD and part of PKS1. Using qRT-PCR (quantitative reverse transcription polymerase chain reaction), we found that the transcription level of the *EthD* gene region was dramatically different from that of *Pks1* in *M*. *acridum* and *M*. *album*, suggesting that the *Pks1* and *EthD* regions were not contained within a single gene (S1 Fig). Further manual annotations and RT-PCR (reverse transcription PCR) analyses indicated this region in *M*. *acridum* and *M*. *album* contained two genes: *Pks1* and *EthD* (S1 Fig). We have deposited the sequences of these newly determined *Pks1* genes in GenBank (*M*. *acridum Pks1*, GenBank accession number: MG385100; *M*. *album Pks1*, GenBank accession number: MG385101).

**Fig 1:**
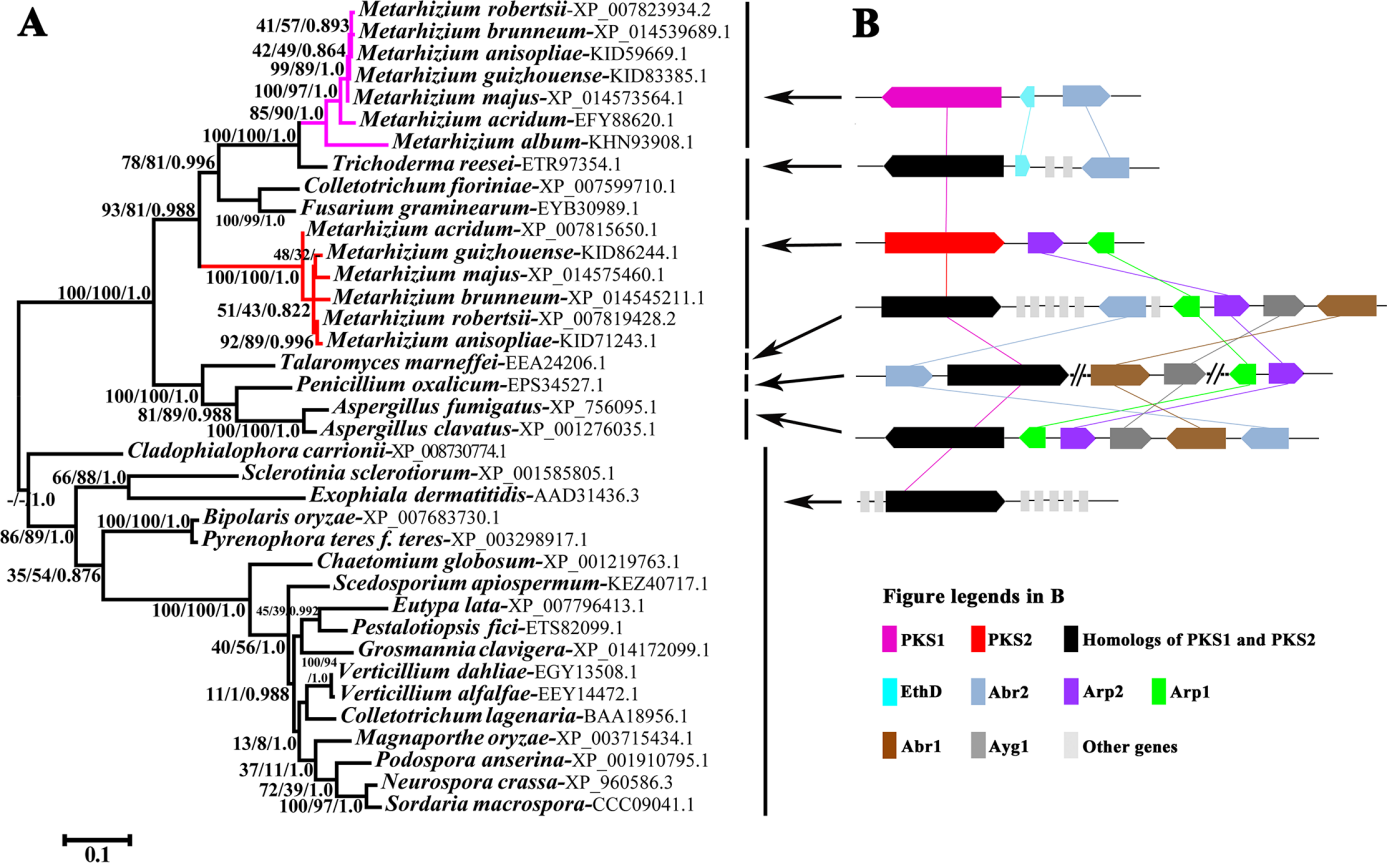
Phylogenetic and syntenic assays of PKS1 and PKS2 from *M*. *robertsii*, and their homologs in other ascomycete fungi. (**A**) Phylogenetic tree based on the amino acid sequences of the PKS KS domains. The Genbank accession number for each full-length amino acid sequence of each PKS is shown after the species name. The clade containing the *Metarhizium* PKS1s is highlighted in pink, that containing PKS2s is highlighted in red. Numbers at nodes represent the bootstrap values of Maximum Likelihood (left), Neighbor-Joining (middle), and the Bayesian posterior probabilities (right). A hyphen (-) indicates no support for the given node in the corresponding method. The scale bar corresponds to the estimated number of amino acid substitutions per site. (**B**) Schematic maps of the gene clusters containing *Pks1*, *Pks2*, or their homologs. Colors in the inset legend represent genes in the cluster; gray bars indicate genes that are not homologous to each other. The groups of fungal species that contain specific gene clusters are indicated with arrows.

As reported in previous studies [5, 16], domains are usually used for phylogenetic analysis of PKSs. We thus used the Batch Search program provided by PFAM (http://pfam.xfam.org/) to analyze the domain structures of 37 PKSs from 31 fungal species (S1 Table). Eight types of domains (S1 Table) were identified in the PKSs, six of which were found in all the PKSs. These six domains are KS-N (N-terminus of β-ketoacyl synthase) (PF00109), KS-C (C-terminus of β-ketoacyl synthase) (PF02801), AT (acyltransferase) (PF00698), PS-DH (polyketide synthase dehydratase) (PF14765), PP-binding (Phosphopantetheine attachment site) (PF00550), and TE (thioesterase) (PF00975). Because whole KS domains are typically used for phylogenetic analysis [5, 16], we used the protein regions (designated as KS domain below) that contained KS-N and KS-C domains. Sequences corresponding to homologous domains across all 37 PKSs were aligned with MUSCLE [18] and used to construct Maximum Likelihood (ML), Bayesian Inference, and Neighbor-Joining phylogenetic trees (Figs 1A and S2). For the trees constructed based on the KS domains, all analyses recovered a major clade of PKS1 and PKS2 proteins with high support (93% for ML; 81% for NJ; 0.988 for Bayesian Inference) (Fig 1A). This major clade was divided into two well-supported clades (Fig 1A). One clade (100% for NJ and ML, 1.0 for Bayesian Inference) contained PKSs from the six *Metarhizium* species, including *M*. *robertsii*’s PKS2 [16]. We thus designated this as the PKS2 clade. The other clade (85% for ML, 90% for NJ and 1.0 for Bayesian Inference), designated as the PKS1 clade, contained PKSs from seven *Metarhizium* species including *M*. *robertsii*’s PKS1 [17]. Phylogenetic analyses based on the other four domains (AT, PP, PS-DH and TE) generated trees with similar topologies to the KS domain tree (S2 Fig). We further compared the topology of the obtained KS domain tree with alternative KS domain trees using CONSEL [19]. The approximately unbiased (au) test showed that the obtained tree was the best supported. The alternative hypothesis, where the PKS1 and PKS2 clades were forced into a sister relationship, was statistically (*P* < 0.05) rejected (S3 Fig, S2 Table, S1 Dataset). The placement of the PKS2 clade outside of the major clade containing PKS1s and PKS2s was also statistically (*P* < 0.05) rejected (S3 Fig, S2 Table, S1 Dataset). The results of the seven other tests (np, bp, pp, kh, sh, wkh and wsh) available in CONSEL were consistent with the AU test: the obtained tree was the most well-supported and alternative trees were statistically (*P* < 0.05) rejected (S3 Fig, S2 Table).

**Fig 2:**
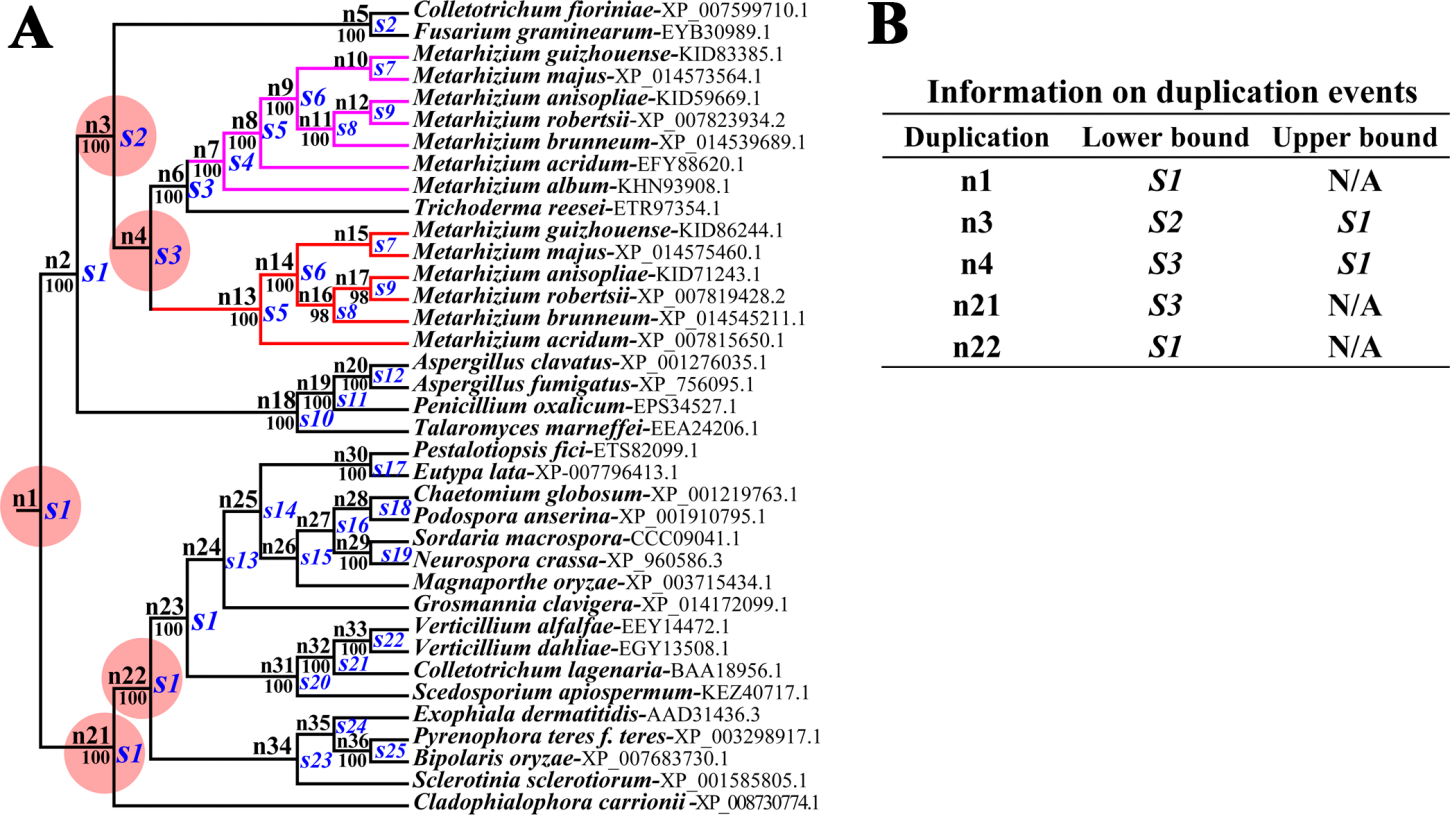
Estimation of *Pks* gene duplication and loss events in the fungal species shown in Fig 1. (**A**) The reconciliation of the rearranged ML tree of the *Pks* genes (Fig 1) with the species tree (S4 Fig) using NOTUNG (v 2.9) with a duplication-loss (DL) model. The predicted loss events are shown in S5 Fig. Red circles indicate duplication events that are also inferred by the reconciliation of the raw ML tree of the *Pks* genes with the species tree (S6 Fig). Numbers at nodes represent Maximum Likelihood bootstrap values. The letter “n” followed by a number indicates an internal node in the rearranged ML tree in the reconciliation assay. The letter “s” followed by a number indicates an internal node species. The clade containing the *Metarhizium* PKS1s is highlighted in pink, that containing PKS2s is highlighted in red. (**B**) The lower and upper bounds of duplication event timing. The upper bound shows the most recent ancestral species where a duplication event was not present, while the lower bound indicates the oldest ancestral species where the duplication event must have been present.

Gene duplication and loss events were then predicted with the NOTUNG [20]. To this end, we first constructed the species tree of the 31 fungal species presented in Fig 1A (S4 Fig, S2 Dataset). To reduce bias resulting from weakly supported branches (< 90%) in the ML tree of the KS domains (Fig 1A); the tree was rearranged with NOTUNG. Using NOTUNG with a duplication-loss (DL) model or a duplication-transfer-loss (DTL) model, the rearranged and the raw ML trees were each separately reconciled with the species tree. For the DL model with default parameters (1.5 for duplication and 1.0 for a loss), reconciliations of the species tree with the raw or rearranged ML trees both estimated gene duplication events at five nodes (Figs 2, S5 and S6). The gene duplication event that generated the *Pks1* and *Pks2* genes in *Metarhizium* species could have occurred at node n3 and n4. The gene duplication event at the node n4 was the latest one, and it is more likely that this event generated *Metarhizium*’s *Pks1* and *Pks2* genes (Fig 2). Therefore, the duplication event that produced *Pks1* and *Pks2* might have occurred in the common ancestor of *Metarhizium* and *Trichoderma* (Fig 2), implying that one of the resulting two paralogs was lost in *M*. *album* and *T*. *reesei* (Figs 2, S5 and S6). Reconciliation assays using the DL model with other parameters generated the same results as that with the default parameters (S5 and S6 Figs). Using the DTL model with several parameter combinations, reconciliation assays also showed that *Pks1* and *Pks2* in *Metarhizium* species resulted from gene duplication, and that the duplication event could have occurred in the common ancestor of *Metarhizium* and *Trichoderma* (S3 Dataset).

As previously reported, there are 20 PKSs in *M*. *robertsii* [2]. We performed phylogenetic analysis combining the KS domains of the 37 PKSs previously analyzed (Fig 1A) with the 18 additional PKSs in *M*. *robertsii*. The resulting tree (S7 Fig) showed that the 18 *M*. *robertsii* PKSs (excluding PKS1 and PKS2) formed clades basal to the major clade containing the 37 PKSs previously analyzed (Fig 1A; including *Metarhizium* PKS1s and PKS2s). This result further indicated that the PKS1s and PKS2s in *Metarhizium* species were two paralogs resulting from gene duplication.

We next examined the genomic context, i.e. the genes upstream and downstream on the chromosome, of *Pks1*, *Pks2* and their homologs in the other fungal species we had used for phylogenetic analysis. Basal to the *Metarhizium* clade was a clade including the Eurotiomycetidae species *Aspergillus fumigatus*, *A*. *clavatus*, *Talaromyces marneffei*, and *Penicillin oxalicum* (Fig 1A). It has been previously shown that the *Pks* gene *Alb1* of *A*. *fumigatus* is contained within a cluster of genes (*Alb1*, *Arp1*, *Arp2*, *Abr1*, *Abr2*, and *ayg1*) encoding DHN-melanin biosynthesis proteins [8]. We also identified this gene cluster in *A*. *clavatus* and *T*. *marneffei* (Fig 1B). In *P*. *oxalicum*, these six genes were divided into three groups widely separated in the genome; each group contained two physically linked genes (Fig 1B). In the six *Metarhizium* species possessing the *Pks2* gene, homologs of *Arp1* and *Arp2* were adjacent to *Pks2*. We designated this gene cluster as *Pks2-gc*. Only the homologs of *Abr2* clustered with *Pks1* homologs in the seven *Metarhizium* species, *T*. *reesei*, *F*. *graminearum*, and *C*. *fioriniae*. In these species, other genes are inserted between the homologs of *Pks1* and *Abr2*, including the homolog of *EthD*; *EthD* now forms part of the *Pks* gene cluster in *M*. *robertsii* [17]. We designated the *Metarhizium* gene cluster containing the *Pks1* gene as *Pks1-gc*. This cluster included *Abr2*, *EthD* and *Pks1*. In the other fungal species shown in the phylogenetic tree (Fig 1A), no homologs of *Arp1*, *Arp2*, *Abr1*, *Abr2*, and *ayg1* were found in the vicinity of *Pks1* and *Pks2* homologs (Fig 1B). Gene clusters similar to *Pks1-gc* and *Pks2-gc* were absent in the fungi basal to the clade containing *Aspergillus*, *Penicillium*, *Talaromyces*, *Metarhizium*, *Fusarium*, *Colletotrichum* and *Trichoderma* fungi (Fig 1A).

We next constructed single gene phylogenies of *Abr2*, *Arp1*, and *Arp2* in the fungi with gene clusters similar to *Pks1-gc* or *Pks2-gc*. The individual gene phylogenies had topologies nearly congruent with that of the *Pks* gene phylogeny (S8 Fig), suggesting that all genes in the two clusters could have followed similar evolutionary paths.

### Diversification of the two *Pks* paralogs in *Metarhizium*

**Fig 3:**
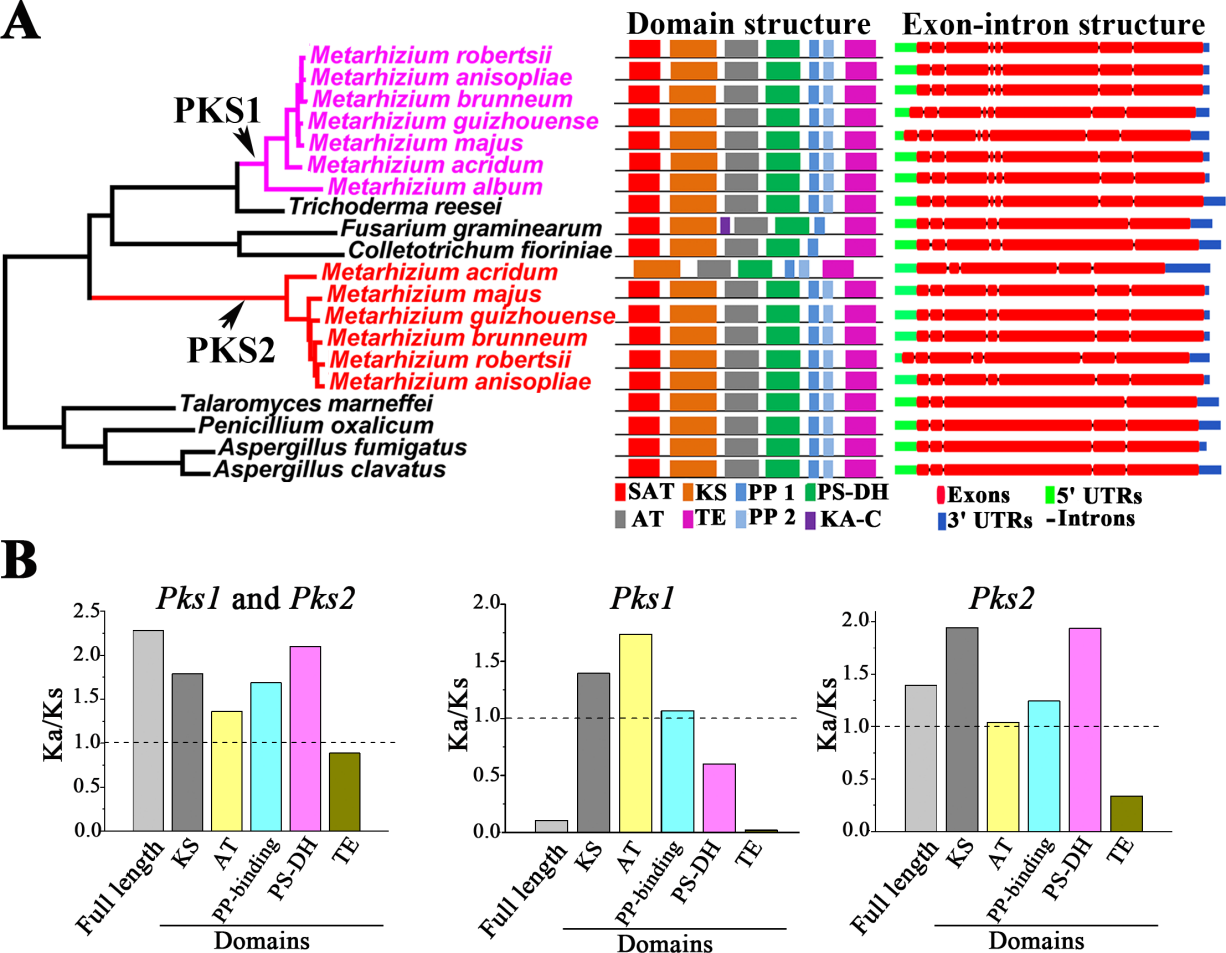
Gene structure diversification and selection pressure acting on *Pks1* and *Pks2* genes. (**A**) Domain and exon-intron structure of *Metarhizium Pks1* and *Pks2* genes and their homologs in the non-*Metarhizium* fungal species that had gene clusters similar to *Pks1-gc* and *Pks2-gc*. Left panel: Summary schematic of the phylogeny shown in Fig 1A. Middle panel: Domain structures drawn according to PFAM analysis. SAT: starter unit acyltransferase (PF16073); KS: the KS domain containing the N-terminal domain of β-ketoacyl synthase (PF00109) and C-terminal domain of β-ketoacyl synthase (PF02801); AT: acyltransferase (PF00698); PS-DH: polyketide synthase dehydratase (PF14765); PP1 and PP2: two copies of the PP-binding domain [phosphopantetheine attachment site (PF00550)]; TE: thioesterase domain (PF00975); KA-C: ketoacyl-synthetase C-terminal extension (PF16197). Note: *M*. *acridum* PKS2 lacks the SAT domain. Right panel: Exon-intron structures of the genomic sequences corresponding to the coding sequences of the *Pks* genes. The exon-intron structure images were prepared with Gene Structure Display Server v2.0 (CBI, Peking University, China). 5*'*UTR: predicted upstream untranslated region; 3*'*UTR: predicted downstream untranslated region. (**B**) Selection pressure acting on the full-length *Pks1* and *Pks2* genes and their domains. Left panel: *Pks1* and *Pks2* were analyzed simultaneously. Middle panel: Only the *Pks1* genes were analyzed. Left panel: Only the *Pks2* genes were analyzed.

Based on our domain analysis results, we drew schematic domain structures for PKS1s and PKS2s in *Metarhizium* species and their homologs in other fungi with gene clusters similar to the *Pks1-gc* and *Pks2-gc* (Fig 3A). Except for *M*. *acridum*’s PKS2 that lacks a SAT domain, the PKS1s and PKS2s contain a SAT domain, a KS domain, an AT domain, a PS-DH domain, two PP-binding domains and a TE domain (Fig 3A). Homologs of PKS1 and PKS2 in *T*. *reesei*, *T*. *marneffei*, *P*. *oxalicum*, *A*. *fumigatus* and *A*. *clavatus* had the same domain structures as PKS1 and PKS2 in *M*. *robertsii*. The homologs in *F*. *graminearum* and *C*. *fioriniae* differed from *M*. *robertsii*’s PKS1 and PKS2 in having only one PP-binding domain. Additionally, *F*. *graminearum* had a KA-C domain not found in the PKS1s or the PKS2s in *Metarhizium* species (Fig 3A).

We next investigated the exon-intron structure of *Pks1*, *Pks2* and their homologs in fungi with gene clusters similar to the *Pks1-gc* or *Pks2-gc*. The exons, introns, 5*'*UTRs and 3*'*UTRs were predicted with Gene Structure Display Server v2.0 (CBI, Peking University, China). For each gene, the introns predicted were the same as those annotated in NCBI (The accession numbers of the analyzed genes are shown in S1 Table). All *Pks1* genes in *Metarhizium* had the same exon-intron structure with seven introns (Fig 3A). *Pks*2 in *M*. *acridum* had four introns; *Pks2* in all other *Metarhizium* species had six introns (Fig 3A). The homolog of *Pks1* and *Pks2* in *T*. *reesei* had the same exon-intron structure as *Pks1* in *Metarhizium* species, but homologs in other non-*Metarhizium* fungi had different exon-intron structures (Fig 3A).

We then used the ratio of non-synonymous to synonymous rate ratio (Ka/Ks) to calculate the extent of selection pressures on the full-length sequences of *Metarhizium’*s *Pks1* and *Pks2* genes, and their individual domains. The Ka/Ks value of the full-length sequences was 2.3 (Fig 3B), suggesting that *Pks1* and *Pks2* genes were under positive selection for beneficial mutants. The selection pressures acting on the domains varied, with the KS, AT, PP-binding, and PS-DH domains under positive selection and the TE domain under purifying selection (Fig 3B). Analysis of the *Pks1* genes separately produced a Ka/Ks value of 0.1 (Fig 3B), indicating that purifying selection dominates, but this only held true for the PS-DH and TE domains. The KS, AT and PP-binding domains were under positive selection (Fig 3B). When the *Pks2* genes were analyzed separately, the Ka/Ks value was 1.4, consistent with overall positive selection (Fig 3B). Only the TE domain in *Pks2* genes was under purifying selection, the other four domains were all under positive selection (Fig 3B). Among the six *Pks2* genes identified in *Metarhizium* species, *M*. *acridum Pks2* gene had the largest Ka/Ks value (3.5) (S9 Fig).

Using protein sequence alignment, we analyzed the amino acid variation in PKS1 and PKS2 domains from *Metarhizium* species, and their homologs in other fungi with *Pks1-gc* and *Pks2-gc* like gene clusters (S4 Dataset). In the KS, PP-binding, PS-DH and AT domains, conserved amino acid residues specific to PKS1 were identified, while their corresponding sites in the PKS2s were changed to other conserved amino acid residues. In the KS domain, six conserved consensus motifs were previously characterized in fungal pigmentation PKSs [21]. An amino acid difference in one of the six motifs was found between PKS1s and PKS2s: the conserved motif sequence was DPGQRL in the PKS1s and DPAQRL in the PKS2s.

**Fig 4:**
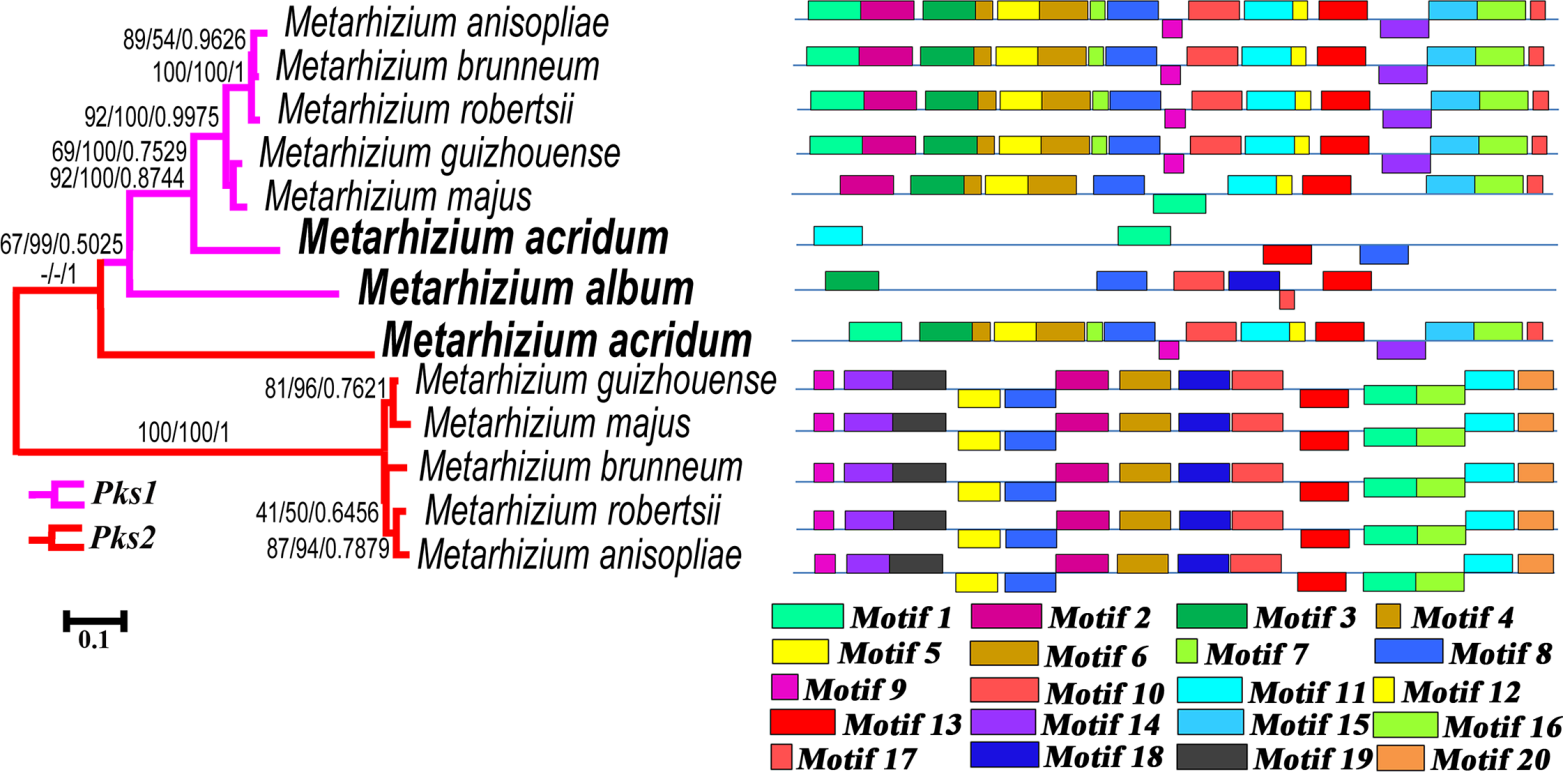
Analysis of the promoters of the *Pks1* and *Pks2* genes in *Metarhizium* species. Left panel: phylogenetic analysis of the promoter regions (645 bp) of the *Pks1* genes (highlighted in pink) and the *Pks2* genes (highlighted in red) across *Metarhizium* species. Numbers at nodes represent bootstrap values of Maximum Likelihood (left), Neighbor-Joining (middle), and Bayesian posterior probabilities (right). Hyphen (-) indicates no support for the given node in the corresponding method. The scale bar corresponds to the estimated number of base substitutions per site. Right panel: the overrepresented motifs in the promoter regions as predicted by MEME (http://meme-suite.org/). Each color block represents a specific motif. Blocks above the horizontal line indicate motifs that are identical to their respective promoter sequences; blocks below the horizontal line indicate motifs that are the reverse complement of their corresponding promoter sequences. Block lengths are proportional to motif lengths. The motif logos and sequences for each gene are presented in S5 Dataset. Note: the *Pks1* promoters in *M*. *acridum* and *M*. *album* differ markedly from those of other *Pks1s*; the *Pks2* promoter in *M*. *acridum* is different from those in other *Metarhizium* species.

We also analyzed the promoter regions of the *Pks1* and *Pks2* genes. Phylogenetic analysis of the promoter sequences (645 base pairs, S5 Dataset) recovered a clade of *Pks1* promoters (Fig 4). The promoter of *Pks2* in *M*. *acridum* clustered with the *Pks1* promoters, but the five other *Pks2* promoters formed a separate clade (Fig 4). We next looked at the overrepresented motifs (motifs that are found in two or more species) in the *Pks1* and *Pks2* promoters using MEME (http://meme-suite.org/); see details of the overrepresented motifs in S5 Dataset. The *Pks1* promoters in the generalist species (*M*. *robertsii*, *M*. *anisopliae*, and *M*. *brunneum*) and the intermediate host range *M*. *guizhouense* had the same motif structure. However, three of these overrepresented motifs (Motif 9, Motif 10, and Motif 14) were absent in *M*. *majus*, a species that also has an intermediate host range (Fig 4). The motif structures of the *Pks1* promoters in the two specialist species (*M*. *acridum* and *M*. *album*) differed substantially, both from each other and from the other five *Metarhizium* species (Fig 4). The three generalists and the two species with intermediate host ranges had 13–17 overrepresented motifs, but only four of these motifs were identified in *M*. *acridum* and only six in *M*. *album*. Furthermore, three of the four overrepresented motifs in *M*. *acridum* had a different directionality as compared to the motifs in the other species. Similarly, the motif structures of the *Pks2* promoters in generalist and intermediate host range species were the same. However, eight of the overrepresented motifs identified in the generalists were not found in the *Pks2* promoter of the specialist *M*. *acridum* (Fig 4).

### Expression and regulation of *Pks1* and *Pks2* genes in *Metarhizium* species

**Fig 5:**
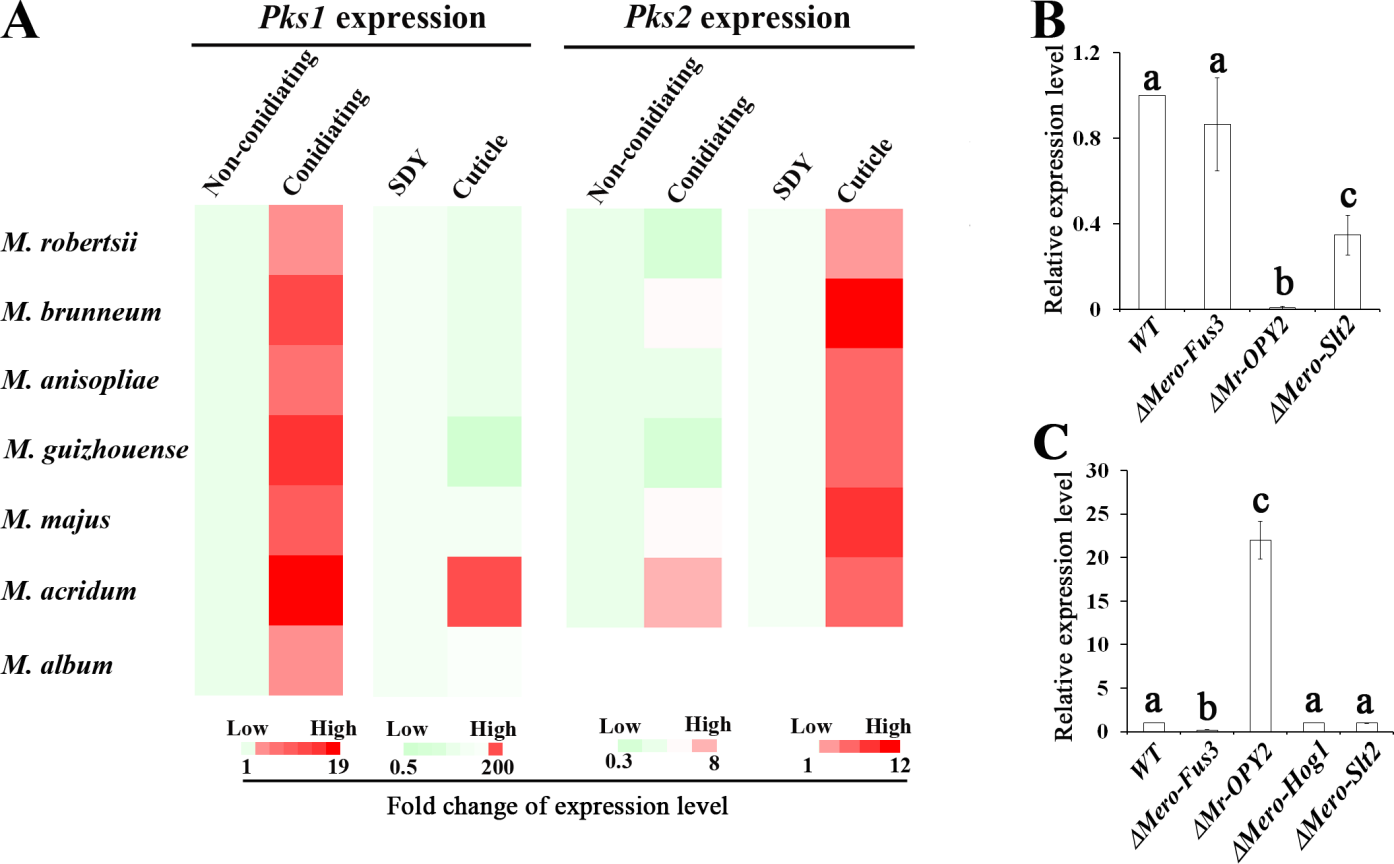
Expression of *Pks1* and *Pks2* genes in seven *Metarhizium* species, and the regulation of the *Pks1* and the *Pks2* genes in *M*. *robertsii*. (**A**) qRT-PCR analysis of *Pks1* and *Pks2* expression during conidiation and cuticle penetration in seven *Metarhizium* species. Gene expression during conidiation (five days after inoculation of conidia on PDA) was calculated relative to that in non-conidiating mycelia (two days after fungal inoculation), which is set to 1. For cuticle penetration, gene expression on a locust cuticle was calculated relative to that in mycelia grown in the nutrient-rich SDY medium, which is set to 1. qRT-PCR analysis of the expression level of *M*. *robertsii*’s *Pks1* (**B**) and *Pks2* (**C**) in the WT, in *ΔMr-OPY2* (a membrane protein), and in the three MAPK mutants (*ΔMero-Hog1*, *ΔMero-Fus3* and *ΔMero-Slt2*) during cuticle penetration. The expression level in the WT is set to 1. Data are expressed as mean ± SE. Values with different letters are significantly different (n = 3, *P* < 0.05, Tukey’s test in a one-way ANOVA). All assays were repeated three times with three replicates per repeat.

Because the promoter regions of *Pks1* and *Pks2* genes were diversified, we investigated whether they have different expression patterns in *Metarhizium* species. Previously, we found that the *Pks1* gene was highly expressed during conidiation in *M*. *robertsii* [17]. Our previously published RNA-seq analyses [22] and the qRT-PCR analyses conducted here showed that *M*. *robertsii Pks2* was upregulated in appressoria-forming germlings on locust cuticle relative to hyphae grown in nutrient-rich SDY (Sabroud dextrose broth plus 1% yeast extract) (Fig 5A). We used qRT-PCR to test whether *Pks1* and *Pks2* genes in the other *Metarhizium* species had the same expression patterns.

Consistent with the gene expression patterns observed in *M*. *robertsii*, *Pks1* expression was upregulated in mycelia conidiating on PDA (potato dextrose agar) as compared to non-conidiating mycelia (Fig 5A). Except for *M*. *acridum*, *Pks1* expression in SDY or on the locust cuticle was the same in *Metarhizium* species (Fig 5A). *Pks1* gene expression in the appressoria-forming germlings of *M*. *acridum* was 500-fold greater than that in SDY (Student’s *t* test, n = 3, *P* < 0.01). *Pks2* gene expression in conidiating *M*. *acridum* mycelia was significantly greater than in non-conidiating mycelia (Student’s *t* test, n = 3, *P* < 0.01); but in the other five *Metarhizium* species, no significant (Student’s *t* test, *P* > 0.05) difference in *Pks2* gene expression was observed between conidiating and non-conidiating mycelia (Fig 5A). Compared to mycelia grown in SDY, *Pks2* genes in all six *Pks2*-containing *Metarhizium* species (*M*. *robertsii*, *M*. *brunneum*, *M*. *anisopliae*, *M*. *guizhouense*, *M*. *majus*, and *M*. *acridum*) were significantly upregulated in appressoria-forming germlings on locust cuticle (Student’s *t* test, *P* < 0.01) (Fig 5A).

In previous studies, we reported that several key signaling pathways were involved in conidial pigmentation and appressorium formation in *M*. *robertsii* [14, 17, 22], and Hog1-MAPK was shown to regulate *Pks1* during conidiation [17]. Using qRT-PCR, we further compared *Pks1* gene expression during conidiation in the wild-type strain (WT) and several signaling mutants. The expression level of *Pks1* in the WT was significantly higher than in *ΔMr-OPY2* and *ΔMero-Slt2* (*P* < 0.05 for both), and *ΔMero-Slt2* expressed more (*P* < 0.05) *Pks1* than *ΔMr-OPY2* (Fig 5B). This suggested that *Pks1* was positively regulated by Mr-OPY2 and Slt2-MAPK. Similarly, we compared *Pks2* gene expression between the WT and the same set of signaling mutants during appressorium formation on locust cuticle. *Pks2* expression by the WT was significantly greater than *ΔMero-Fus3*, but lower than *ΔMr-OPY2* (n = 3, *P* < 0.05, Tukey’s test in one-way ANOVA) (Fig 5C), suggesting that the *Pks2* gene is positively regulated by Fus3-MAPK and negatively regulated by Mr-OPY2.

### *Pks1* is involved in conidial pigmentation and tolerance to environmental stresses

**Fig 6:**
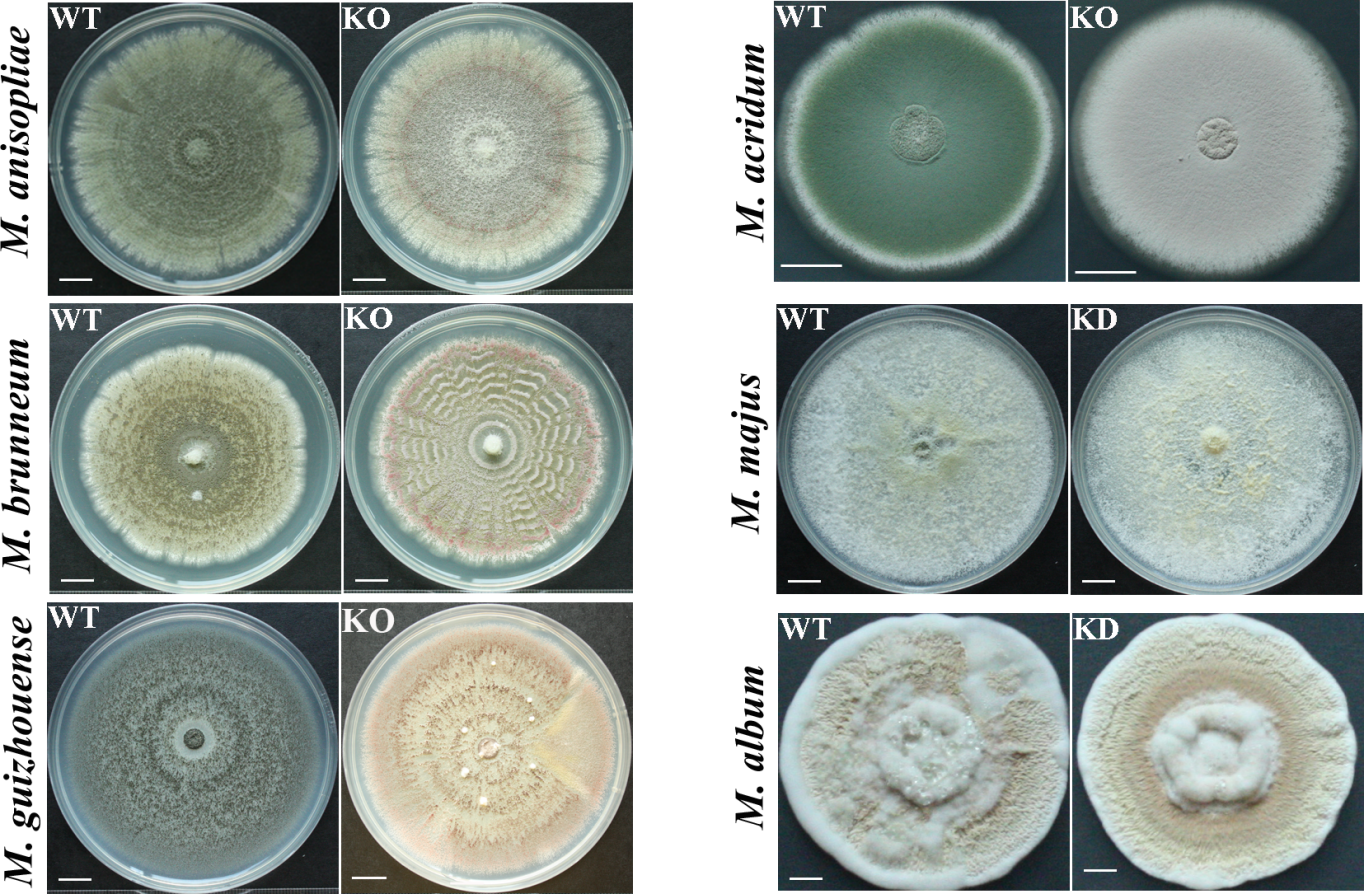
Morphology of colonies of *Pks1* gene mutants and their respective parental wild-type strains. WT: the wild-type strain; KO: *Pks1* knockout mutants; KD: *Pks1* knockdown mutants. Pictures were taken 18 days after inoculation with 5 μl of conidial suspension (4×10^7^ conidia/ml) on a PDA plate. Scale bars represent 1 mm in *M*. *album* and 10 mm in *M*. *anisopliae*, *M*. *brunneum*, *M*. *guizhouense*, *M*. *majus*, and *M*. *acridum*.

Except for *M*. *album* that produces nearly white conidia, *Metarhizium* species produce conidia with pigments ranging from light to dark green (Fig 6). Previously, we constructed *Pks1* KO (knock out) mutants of *M*. *robertsii* [17]. Here, we successfully constructed *Pks1* KO mutants for four other species (*M*. *anisopliae*, *M*. *brunneum*, *M*. *guizhouense*, and *M*. *acridum*) (S10 Fig). Inability to clone the very large *Pks1* genes into a plasmid for *Agrobacterium tumefaciens*-mediated fungal transformation precluded complementation of the KO mutants. We therefore selected three independent KO isolates for each mutant. As these three isolates did not differ in any subsequent analyses, we only present data for one isolate/mutant in the main text; data for the two other isolates are shown in the supplementary figures and tables. RNAi (RNA interference) was used to KD (knock down) *Pks1* in *M*. *majus* and *M*. *album* (S10 Fig). Three independent isolates for each KD mutant were selected for further analysis. As these three isolates did not differ in any subsequent analyses, we only present data for one isolate per mutant in the main text, and data for the two other isolates are shown in the supplementary figures and tables.

As with the *M*. *robertsii Pks1* mutant, *Pks1* KO mutants of *M*. *anisopliae*, *M*. *brunneum*, *M*. *guizhouense*, and *M*. *acridum* all produced red conidia (Figs 6 and S11). The conidial color of the *Pks1* KD *M*. *majus* mutant was lighter than that of its parental WT strain (Figs 6 and S11). Conidia of the *Pks1* KD *M*. *album* mutant were almost white, identical in color to the conidia of its parental WT strain (Figs 6 and S11).

Conidial pigments have long been thought to be involved in abiotic stress tolerance in fungi [13, 17]. Therefore, we investigated the involvement of *Pks1* in tolerating UV radiation and temperature stresses. Under optimal conditions (26°C in 1/2 SDY), the deletion of *Pks1* had no impact on conidial germination in *M*. *anisopliae*, *M*. *brunneum*, and *M*. *guizhouense*, as indicated by the GT_50_ (time taken for 50% of the conidia to germinate) (S3 Table). However, compared to their respective WT strains, GT_50_ was significantly reduced in the *Pks1* mutants of *M*. *robertsii*, *M*. *majus*, *and M*. *album* (Student’s *t* test, n = 3, *P* < 0.05), and was significantly increased in the *Pks1* mutant of *M*. *acridum* (Student’s *t* test, n = 3, *P* < 0.05) (S3 Table). In previous studies [e.g. 23], relative germination inhibition (defined in the Materials and Methods) has been used to show fungal tolerance to abiotic stresses. Similar to *M*. *robertsii* [17], the deletion of *Pks1* significantly reduced the UV tolerance of *M*. *anisopliae* and *M*. *brunneum* (Student’s *t* test, n = 3, *P* < 0.05) (Tables 1 and S4). Knocking out or knocking down *Pks1* had no impact on UV radiation tolerance in *M*. *guizhouense*, *M*. *acridum*, or *M*. *album* (Tables 1 and S4). Compared to their respective WT strains, heat stress tolerance was significantly (*P* < 0.05 for all) reduced in the *Pks1* mutants of *M*. *robertsii*, *M*. *guizhouense*, and *M*. *album* (Tables 1 and S4). Cold stress tolerance was reduced only in the *Pks1* mutant of *M*. *album* (Tables 1 and S4). In contrast to all other *Metarhizium* species, the *Pks1* mutant of *M*. *majus* germinated significantly faster than the WT strain under UV and cold stress (Student’s *t* test, n = 3, *P* < 0.05) (Tables 1 and S4).

**Table 1:** Relative germination rates of the *Pks1* mutants of seven *Metarhizium* species, and their respective wild-type strains (WT), under three abiotic stresses. Within the same abiotic stress treatment, values for the same species that are followed by different letters are significantly different (*P* < 0.05, Tukey’s test in a One-way ANOVA). All assays were repeated three times with three replicates per repeat.

	UV radiation		Heat stress		Cold stress	
	*WT*	*ΔPks1-#1*	*WT*	*ΔPks1-#1*	*WT*	*ΔPks1-#1*
*M*. *robertsii*	Ref 17^1^	Ref 17^1^	0.47±0.03^a^	0.71±0.13^b^	2.16±0.15^a^	2.42±0.33^a^
*M*. *anisopliae*	0.21±0.04^a^	0.47±0.01^b^	0.40±0.07^a^	0.49±0.11^a^	2.92±0.17^a^	2.96±0.21^a^
*M*. *brunneum*	0.27±0.03^a^	0.38±0.02^b^	1.16±0.14^a^	1.35±0.21^a^	2.17±0.12^a^	2.09±0.09^a^
*M*. *guizhouense*	0.33±0.03^a^	0.33±0.01^a^	0.69±0.04^a^	1.46±0.23^b^	3.02±0.11^a^	2.64±0.34^a^
*M*. *majus*	0.27±0.03^a^	0.16±0.03^b^	2.23±0.41^a^	2.44±0.58^a^	2.59±0.11^a^	1.44±0.1^b^
*M*. *acridum*	0.47±0.02^a^	0.52±0.1^a^	0.16±0.02^a^	0.09±0.01^a^	2.47±0.04^a^	2.42±0.24^a^
*M*. *album*	0.38±0.04^a^	0.36±0.09^a^	1.86±0.04^a^	5.10±0.49^b^	1.61±0.03^a^	3.30±0.20^b^

Note:

1: Data published in reference 17; deletion of the *Pks1* gene significantly reduced the tolerance of *M*. *robertsii* to UV radiation.

The numerical values in the table: the relative germination inhibition of a given stressor on each strain was calculated as (Gc-Gt)/ Gc, where Gc and Gt denote the GT_50_ (Time taken for 50% of conidia to germinate) of the stressed and unstressed conidia, respectively.

### *Pks2* is involved in pathogenicity

**Fig 7:**
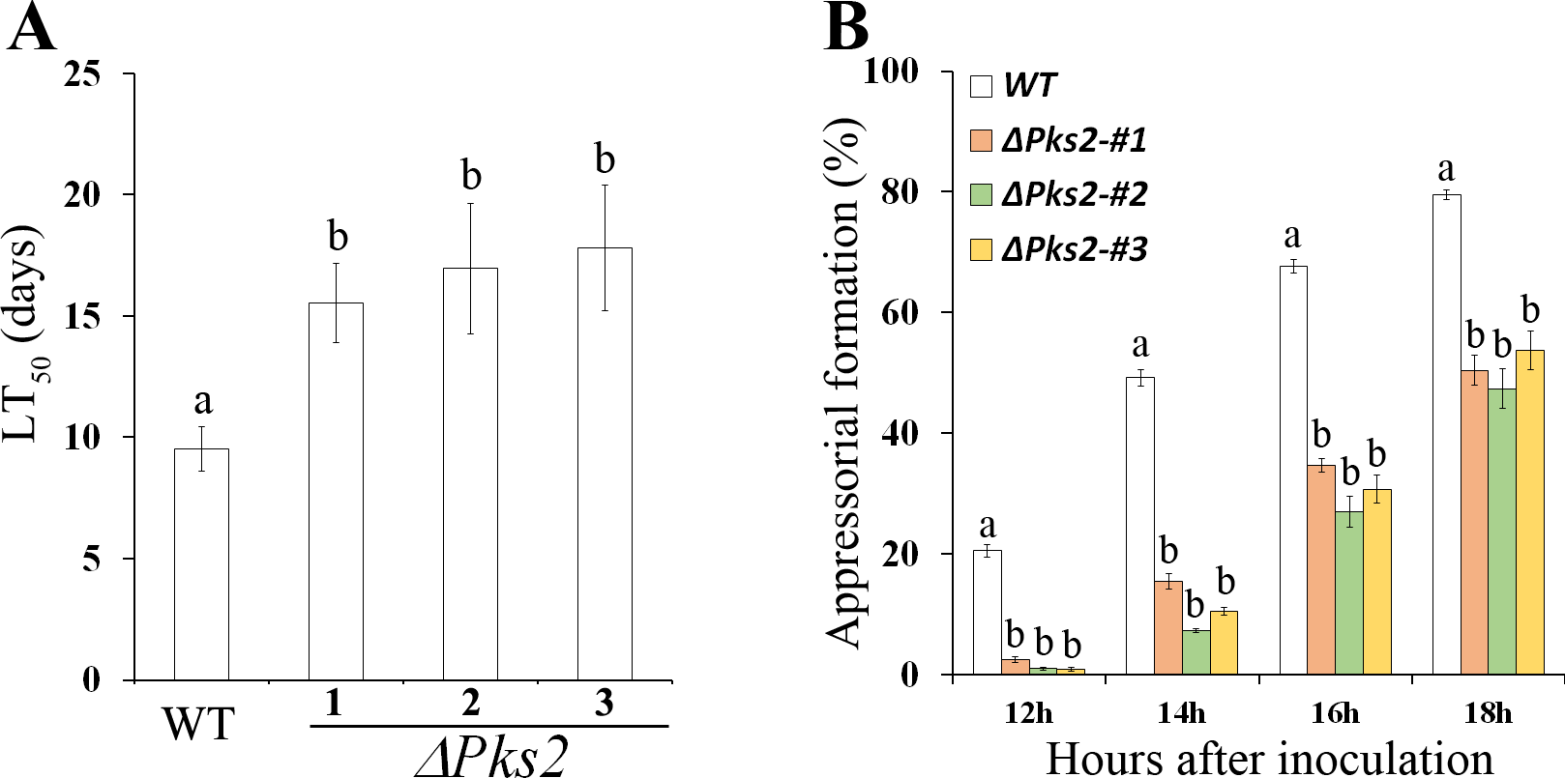
Pathogenicity of three independent isolates of the *Pks2* KO mutant in *M*. *robertsii* (*ΔPks2*) and the WT strain. **(A)** LT_50_ values of WT and three independent isolates of *ΔPks2*. LT_50_: the time taken to kill 50% of the insects. Insects were inoculated by topical application. Bioassays were repeated three times with 40 insects per repeat. Data are expressed as the mean ± SE. Values with different letters are significantly different (*P* < 0.05, Tukey’s test in a one-way ANOVA). (**B**) The percent of appressoria-forming germlings in the WT and in three independent isolates of *ΔPks2* (*ΔPks2-#1*, *ΔPks2-#2* and *ΔPks2-#3*) on a hydrophobic plastic surface. Appressorium formation assays were repeated three times with three replicates per repeat. Data are expressed as the mean ± SE. At each time point, values with different letters are significantly different (*P* < 0.05, Tukey’s test in a one-way ANOVA).

*M*. *robertsii* is a model organism for the study of entomopathogenicity [17]. We therefore knocked out *Pks2* in this species to investigate its involvement in pathogenicity. The conidial pigmentation of the *Pks2* KO mutant (*ΔPks2*) did not differ from that of the WT (S12 Fig). *ΔPks2* was not different from WT in tolerance to abiotic stresses including UV radiation, heat and cold stress (S5 Table). Compared to the WT, the LT_50_ (the time taken to kill 50% of insects) value of *ΔPks2* was significantly increased (*P* < 0.05, Tukey’s test in one-way ANOVA) (Fig 7A). In addition, compared to the WT, appressorial formation in *ΔPks2* was delayed on a hydrophobic surface (Fig 7B). However, the turgor pressure of the *ΔPks2* appressoria was the same as that of the WT (S13 Fig). Fluorescent staining with Calcofluor white Brightener 2B showed that the fluorescent intensity of the *ΔPks2* appressoria did not differ from that of the WT, suggesting that deletion of *Pks2* did not alter cell wall structure or the composition of the appressoria (S13 Fig).

Our results suggest that *Pks2* is an important factor in pathogenicity. The *Pks2* and *Arp2* genes in the *Pks2-gc* are lacking in the basal specialist *M*. *album*. We postulated that the absence of the complete *Pks2*-gc in *M*. *album* was related to development of host specificity. To test this hypothesis, we constructed a *M*. *album* strain that expressed the *Pks2* and *Arp2* of *M*. *robertsii* (S14 Fig). Bioassays showed that, similar to WT *M*. *album*, *M*. *album* expressing *Pks2* and *Arp2* was still unable to infect *G*. *mellonella* (Lepidoptera) or *Drosophila melanogaster* (Diptera), indicating that the *Pks2-gc* is not sufficient to broaden the host range of *M*. *album*.

### PKS1 and PKS2 synthesize different SMs

**Fig 8:**
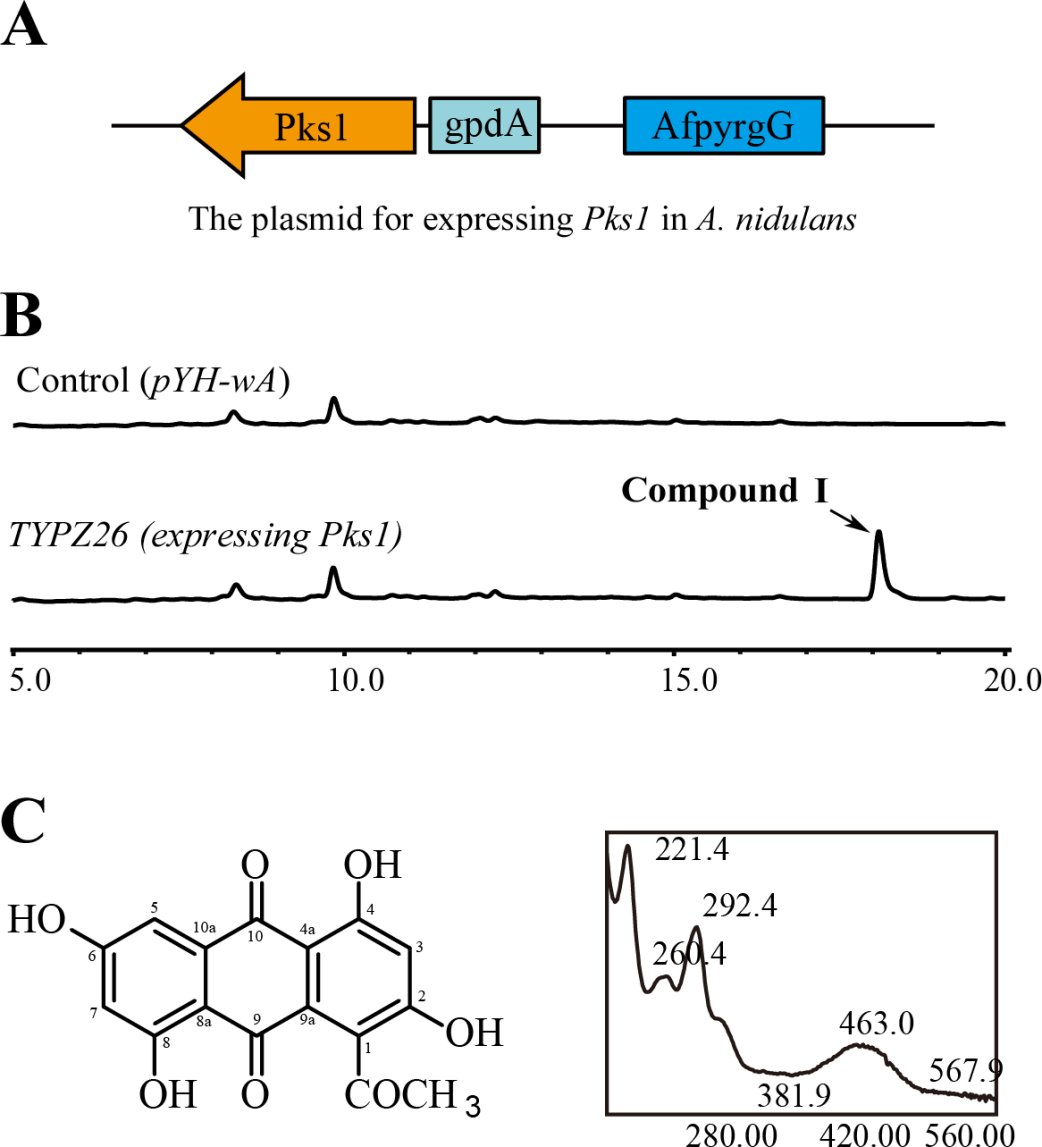
Heterologous expression of *M*. *robertsii Pks1* in *A*. *nidulans* and SM identification. (**A**) Vector of *Pks1* heterologous expression, driven by the constitutive promoter *gpdA* of the glycerol-3-phosphate dehydrogenase from *A*. *nidulans*. (**B**) HPLC analysis of crude extracts from the control strain that was transformed with the empty vector *pYH-wA* (control) or the TYPZ26 strain expressing the *Pks1* gene from *M*. *robertsii*. The target compound (Compound I) is indicated with an arrow. (**C**) Determination of the structure of Compound I. Left panel: chemical structure of Compound I based on NMR and LC-MS analysis (see also S6 Table, S16, S17 and S18 Figs). Right panel: UV-visible absorption of Compound I. Note: Compound I has high UV-visible absorption around 221.4, 260.4, 292.4, and 463.0 nm.

We were unable to identify the SMs synthesized by *M*. *robertsii* PKS1 and PKS2 just by comparing the SM profiles of the *Pks1* and *Pks2* KO mutants with WT. We thus introduced the *Pks1* and *Pks2* genes of *M*. *robertsii* into *A*. *nidulans*, under control of the constitutive promoter *gpdA* of the *A*. *nidulans* glycerol-3-phosphate dehydrogenase gene (Fig 8A), as this fungus and promoter have been used previously for heterologous expression of *Pks* genes [9, 24]. Successful insertion of *Pks1* and *Pks2* into the genome of *A*. *nidulans* was confirmed with PCR (S15 Fig). Compared to the control strain (with an empty expression vector inserted), HPLC (high-performance liquid chromatography) analysis did not identify any new peaks in the transformant expressing *Pks2*, but did identify a new peak at about 18 min in the transformant (designated as TYPZ26) expressing *Pks1* (Fig 8B). This peak indicated a possible compound (designated as Compound I) produced by PKS1. We fermented the transformant TYPZ26 on a large scale (in 10 L batches) to obtain sufficient Compound I for characterization. After semi-preparative reverse-phase HPLC separation, Compound I was purified. The molecular formula of Compound I was determined to be C_15_H_10_O_7,_ based on HR-ESI-MS (m/z 315.0503 [M+H]^+^) (Figs 8C and S16). Analysis of the ^1^H (S17 Fig) and ^13^C NMR spectroscopic data (S18 Fig, S6 Table) for compound I showed that its structure was consistent with that of 1-acetyl-2,4,6,8-tetrahydroxy-9,10-anthraquinone [25], indicating that compound I was an anthraquinone derivative. As we were unable to identify a PKS2-derived SM in *A*. *nidulans* strain expressing *Pks2*, we investigated whether PKS2 synthesized the same SM as PKS1. We did this by introducing into the *ΔPks1* mutant a *Pks2* gene driven by the constitutive promoter *Ptef* of the translation elongation factor gene *tef* in *Aureobasidium pullulans* [26]. qRT-PCR analysis showed that the expression of *Pks2* in three independent isolates of the resulting strain *ΔPks1׃׃Pks2*^*OE*^ was over 50-fold greater than the *ΔPks1* mutant (Student’s *t* test, *P* < 0.01) (S19 Fig). However, the three isolates of the *ΔPks1::Pks2*^*OE*^ strain produced the same red conidia as *ΔPks1* (S19 Fig), and HPLC analysis showed that they did not synthesize Compound I (S19 Fig), suggesting that *Pks2* does not complement *ΔPks1* and, therefore, that PKS1 and PKS2 synthesize different SMs.

## Discussion

We report here that the two *Pks* gene clusters (*Pks1-gc* and *Pks2-gc*) in *Metarhizium* species resulted from gene cluster duplication. Phylogenetic analyses of the *Pks* genes and gene duplication predictions showed that the ancestral gene cluster likely duplicated in an ancestral Hypocrealean fungus. The resulting two gene clusters have been retained in the *Metarhizium* fungi, but one has been lost in non-*Metarhizium* fungi, which have only one gene cluster similar to *Pks1-gc* and *Pks2-gc*. The basal specialist *M*. *album* lacks *Pks2* and *Arp2*, indicating that it has lost *Pks2-gc*.

In *Metarhizium* species, *Pks1-gc* and *Pks2-gc* contained three genes. The other hypocrealean species *T*. *reesei* also had three genes in the *Pks* gene cluster. In contrast, *A*. *fumigatus* and other species basal to hypocrealeans have six genes in their *Pks* gene clusters. This discrepancy may have arisen from gene loss after gene cluster duplication in the ancestral Hypocrealean fungus. Gene loss is an established evolutionary mechanism for the diversification of *Pks* gene clusters after gene duplication [3, 4].

Our phylogenetic and genomic synteny analyses indicated that *Pks1* and *Pks2* genes in the *Pks1-gc* and *Pks2-gc* like gene clusters of Hypocremycetidae and Eurotiomycetidae were more closely related than homologs of *Metarhizium*’s *Pks1* and *Pks2* outside the clusters. Therefore, the phylogeny of the *Pks* genes is incongruent with previously published species-level phylogenies [27]. A possible explanation is that the common ancestor of the Hypocremycetidae and Eurotiomycetidae had an ancestral gene cluster resembling *Pks1-gc* and *Pks2-gc*, which has been retained in some descendants (such as *Aspergillus* and *Metarhizium*), but broken up in others (such as *Neurospora crassa* and *Magnaporthe oryzae*). We identified a possible intermediate separation of the *Pks* gene cluster in *P*. *oxalicum*: the six physically linked genes found in *Aspergillus* species were divided into three groups of two genes in *P*. *oxalicum*. *Pks* genes within gene clusters may have been subject to similar selection pressures, whereas selection pressures on their unclustered homologs diverged, resulting in the incongruence between the *Pks* gene phylogeny and the fungal species-level phylogeny. Alternatively, the common ancestor of Hypocremycetidae and Eurotiomycetidae may have lacked the *Pks* gene cluster, and this cluster has formed independently in the Hypocremycetidae and Eurotiomycetidae, which had the gene cluster. This seems less likely, because the chance that an identical gene cluster would develop independently in in such distantly related fungi is low.

Our results indicate that the two *Pks* paralogs in *Metarhizium* have diversified in several different ways. The promoter regions of *Pks1* and *Pks2* diversified, which could be attributed to their having different gene expression patterns. Consistent with which, we found that different upstream signaling pathways regulated *Pks1* and *Pks2*. Exon-intron structure has also diversified, as indicated by the difference in intron number between *Pks1* and *Pks2*. Changes in exon-intron structure may also affect expression patterns and splicing [28].

*Pks2* did not complement the *Pks1* deletion mutant in *M*. *robertsii*, suggesting that these two paralogs synthesize different SMs. Therefore, mutations in the coding sequences of the two paralogs (*Pks1* and *Pks2*) in the *Metarhizium* genus resulted in neofunctionalization. *Pks1* was highly expressed during conidiation in all seven *Metarhizium* species we tested; *Pks2* was highly expressed during cuticle penetration in the six *Pks2*-containing *Metarhizium* species. In contrast to other *Metarhizium* species, the two *Pks* genes in *M*. *acridum* were both highly expressed during conidiation and cuticle penetration, which might explain the different biological features of *M*. *acridum*. Compared to other *Metarhizium* species, *M*. *acridum* shows higher tolerance to abiotic stress [29]. Although a couple of the *Metarhizium Pks1* gene domains are under positive selection, purifying selection dominates over most of their length. In contrast, *Metarhizium Pks2* genes are under positive selection, but it remains to be determined whether mutations retained by such selection diversified the biochemical functions of PKS2s.

Anthraquinone derivatives have been widely applied in industry. Many are used as fabric dyes and additives to mordant [30]. Anthraquinone derivatives are not highly toxic and have various pharmacologically-relevant effects, including anti-inflammatory, antiviral, antimalarial, antifungal, hypotensive and analgesic, antioxidant, and moderately antitumoral [31]. Anthraquinone derivatives have been found in the fungus *G*. *lavendula* [25]. Here we found that *M*. *robertsii*’s PKS1 synthesized an anthraquinone derivative. This anthraquinone derivative was successfully produced in *A*. *nidulans* on a large scale for future assays of its biological activity. Previous studies have shown that the homologs of *M*. *robertsii* PKS1 synthesize SMs other than anthraquinone including pentaketide in *Colletotrichum orbiculare* and *Pestalotiopsis fici* [9, 32], hexaketide in *Exophiala dermatitidis* [33], and heptaketide in *A*. *fumigatus* PksP/ALB1[34]. The *Pks1* gene cluster in *Metarhizium* species is potentially involved in the synthesis of a previously unreported pigment in fungi. Because disrupting *Pks1* in *Metarhizium* species resulted in red conidia, the pigment synthesized by the cluster *Pks1-gc* could be combined (or react) with the red pigment to form the characteristic green pigment in the WT strain.

The *Pks1*was highly expressed during conidiation in *M*. *album*, but this fungus produced nearly white conidia. This could be due to functional diversification of *Pks1* in *M*. *album*. Alternatively, *Pks1* may only contribute to pigmentation when the red pigment is produced by other genes that might be absent in *M*. *album*.

Fungal tolerance to abiotic stress is multifactorial [13], and conidial pigments act with other components to tolerate abiotic stresses [35]. The contributions of conidial pigments to abiotic stress tolerance vary among *Metarhizium* species [35]. This is supported by our functional characterization of the conidial pigmentation gene *Pks1* in seven *Metarhizium* species. In five *Metarhizium* species, *Pks1* was involved in the tolerance to at least one of the tested abiotic stressors (UV radiation, cold, and heat). However, in *M*. *acridum*, the deletion of *Pks1* had no impact on tolerance to the tested abiotic stresses, while knocking down *Pks1* increased germination rates in *M*. *majus* exposed to UV radiation and cold stress.

In summary, we reported that a gene cluster duplication and subsequent diversification resulted in two *Pks* gene clusters in the genus *Metarhizium*. The resulting two PKSs synthesize different SMs. *Pks1* is highly expressed during conidiation and contributes to conidial pigmentation that provides protection from UV radiation, heat and cold stress. UV radiation and heat stress are the major factors for controlling *Metarhizium*’s population in nature [13, 36]. The *Pks2* gene is a pathogenicity factor that facilitates infection of insects by *M*. *robertsii*. Efficient infection of insects is also important for the survival of *Metarhizium* in the environment because entomopathogenicity enables *Metarhizium* to escape competition from other microbes and build up population levels above the carrying capacity of the rhizosphere [12]. Therefore, duplication and subsequent diversification of a *Pks* gene cluster increased the adaptive flexibility of *Metarhizium* species.

## Materials and methods

### Fungal and bacterial strains

*Metarhizium robertsii* ARSEF2575, *M*. *album* ARSEF1941, *M*. *majus* ARSEF297, *M*. *guizhouense* ARSEF977, *M*. *brunneum* ARSEF3297, and *M*. *anisopliae* strain ARSEF549 were obtained from the Agricultural Research Service Collection of Entomopathogenic Fungi. *M*. *acridum* CQMa 102 was a gift from Prof. Yuxian Xia at the Chongqing University China. The deletion mutants of the gene *Fus3-*, *Slt2-*, and *Hog1-MAPK* and *Mr-OPY2* were previously reported [14, 22]. *Escherichia coli* strain DH5α was used for plasmid construction. *Agrobacterium tumefaciens* AGL-1 was used for *Metarhizium* transformation. The *A*. *nidulans* strain LO8030 was used for the heterologous expression of the *Pks* genes as previously described [9, 24]. *Saccharomyces cerevisiae* strain BJ5464-NpgA was used as the host for DNA assembly [37]. More information about the fungal strains, bacterial strains, and plasmids is given in S7 Table.

### Phylogenetic analysis

*M*. *robertsii* PKS1 or PKS2 were used as queries for BLASTP analysis in NCBI, and the protein sequence of the best hit (e-value cutoff 1e^-05^) in an Ascomycota species was retrieved for phylogenetic analyses. The domains of the obtained PKSs were determined using the Batch Search program provided by PFAM (http://pfam.xfam.org/). Based on these results, the domain sequences were manually extracted from the PKSs. For phylogenetic analyses of the domains, domain sequences were aligned using MUSCLE v3.7 with default parameters [18]. Protein alignments were manually refined and end-trimmed to eliminate poor alignments and divergent regions. Unambiguously aligned positions were used to construct a ML tree with MEGA 6.0 (gap treatment: use all sites; model of evolution: WAG+G; 100 bootstrap replications) [38]. We also constructed an NJ tree with default parameters (gap treatment:pairwise deletion; 1000 bootstrap replications) in MEGA 6.0. A Bayesian inference tree was constructed with MrBayes v3.2.5 as described [39]; the model of evolution was WAG + G. For each Bayesian analysis, four Metropolis-coupled chains were used. Each analysis ran for 5,000,000 generations, with sampling every 1000 generations (‘mcmc ngen = 5000000 samplefreq = 1000’). The analysis was considered finished when the average standard deviation of the split frequencies was 0.01 or less. The first 25% of all trees were removed as burn-in.

To evaluate the confidence of all topology hypotheses of the KS domain tree, site-wise log likelihoods of all alternative topologies were calculated with PhyML-3.1[40]. Then, the site-wise log likelihoods file was used as input to estimate the *P*-values for each alternative hypothesis using the Approximately Unbiased (au) test, the Bootstrap Probability (np, bp) test, the Posterior Probabilities (pp) test, the Kishino-Hasegawa (kh) test and the Shimodaira-Hasegawa (sh) test implemented in the program CONSEL [19].

The protein sequences of Abr2, Apr1 or Apr2 in the *Pks1-gc* or *Pks2-gc* gene clusters were obtained as described below (Microsynteny analysis of *Pks1* and *Pks2*), and their phylogenetic analyses were conducted as described for the PKS domains.

### Species tree construction

The tree of the 31 fungal species analyzed in Fig 1A was constructed using the best scoring single-copy genes as previously described [5]. Previously, 23 genes were used [5], but here only 20 orthologs (S8 Table) were successfully retrieved from the 31 fungal species with BLASTP (e value cutoff e^-05^) using *Saccharomyces cerevisiae* genes as queries. Therefore, the 20 single-copy genes were used to construct the species tree. The ortholog protein sequences were aligned using MUSCLE 3.7 [18], which was then manually refined and end-trimmed to eliminate poor alignments and divergent regions. The resulting alignments were concatenated (S2 Dataset) to construct an ML tree with MEGA 6.0 (gap treatment: use all sites; model of evolution: LG+G+I; 100 bootstrap replications; ML heuristic method: Nearest-Neighbor-Interchange), an NJ tree with default parameters (gap treatment: pairwise deletion; 1000 bootstrap replications) in MEGA 6.0. A BI tree was conducted with MrBayes v3.2.5 using the LG+G+I model. For each analysis, we ran four Metropolis-coupled chains for 5,000,000 generations, sampling every 1000 generations (‘mcmc ngen = 5000000 samplefreq = 1000’). The analyses finished with an average standard deviation of split frequencies of 0.01 or less. The first 25% of trees were removed as burn-in.

### Tree rearrangement, reconciliation, and gene duplication predication

To avoid overestimating duplications in the KS domain ML tree that had several nodes with weak sequence support, the tree was rearranged using NOTUNG v. 2.9 [20]. The standard parsimony weight parameters of NOTUNG were used: 1.5 for duplication and 1.0 for loss. We used 90 as the bootstrap cut-off value for weak branches.

The rearranged and raw ML trees were then reconciled with the species tree to predict gene duplication and loss using NOTUNG using duplication-loss (DL) or duplication-transfer-loss (DTL) model [20]. For each model, several parameter combinations were used.

### Microsynteny analysis of *Pks1* and *Pks2* genes

The microsynteny of the genomic regions flanking *Pks1* and *Pks2* genes was manually analyzed using BLASTP. We analyzed the 20 genes flanking each *Pks* gene to identify homologs (BLASTP, e-value cutoff 1e^-05^) to the four genes (*Abr2*, *Apr1*, *Apr2* and *EthD*) comprising the *Pks1-gc* or *Pks2-gc* in *M*. *robertsii* [17], and the five genes (*Abr1*, *Abr2*, *Apr1*, *Apr2* and *Ayg1*) comprising the *Pks* (*alb*) gene cluster in *A*. *fumigatus* [8].

### Selection pressure

Based on the coding sequences of *Pks1* and *Pks2* genes and their protein sequences from the seven *Metarhizium* species, the Ka/Ks ratio was calculated as previously described [28]. Briefly, protein sequences were aligned with MUSCLE v3.7 [18], which guided the alignment of the coding sequences with PAL2NAL [41]. Based on alignments of coding sequences and protein sequences, we calculated Ka, Ks, and the Ka/Ks using the MYN algorithm of the KaKs_Calculator v2.0 [42].

### Preparation of total RNA during conidiation and cuticle penetration

Fungal total RNA was extracted with TRIzol reagent (Life Technologies, USA). Non-conidiating and conidiating mycelia were prepared as previously described [17]. Briefly, 100 μl of conidial suspension (1×10^7^ conidia/ml) was evenly spread on a PDA plate (diameter = 90mm, BD, USA) and incubated at 26°C. Mycelia at 2 d and 5 d post incubation were collected as non-conidiating and conidiating mycelia, respectively.

Gene expression during saprophytic growth was compared to cuticle penetration. For saprophytic growth, conidia (1×10^6^ conidia/mL) were grown at 26°C for 36 h in SDY. For cuticle penetration, appressoria-forming germlings on the hindwings of *Locusta migrattoria manilensis* were prepared as previously described [14].

### qRT-PCR

qRT-PCR analysis was conducted as previously described [22]. Complementary DNAs (cDNAs) were synthesized with ReverTra AceqPCR RT Master Mix (Toyobo, Japan). Quantitative RT-PCR analysis was performed using Thunderbird SYBR qPCR Mix without ROX (Toyobo, Japan). *Act* and *tef* were used as internal standards [43]. The relative normalized gene transcription level was computed using the 2^-ΔΔCt^ method [44]. All qRT-PCR assays were repeated three times with three technical replicates per repeat. Primers used in this study are listed in S9 Table.

### Gene knockout and knockdown in *Metarhizium* species

Gene knockout based on homologous recombination was conducted as previously described [45]. Around 1kb of DNA fragment corresponding to the N-terminus of a PKS protein was deleted.

Gene knockdown using RNA interference was performed as previously described [46] with modifications. In brief, to construct a knockdown vector, the promoter region [645bp (base pair)] of the target gene was amplified with PCR using High-fidelity Taq DNA polymerase (Toyobo, Japan), and cloned into the plasmid pPK2-bar-GFP [14] to produce the plasmid pPK2-bar-GFP-Pro. To produce the genetic dsRNA, a 30 bp sense and anti-sense sequences (30bp) corresponding to the target gene were added to the forward and reverse primers to amplify the loop DNA fragment (150bp) with PCR. The PCR product was then cloned downstream of the promoter of the target gene in the plasmid pPK2-bar-GFP-Pro to produce the RNAi plasmid pPK2-bar-GFP-RNAi, which was then transformed into the WT *Metarhizium* species via *A*. *tumefaciens* AGL1. Transformants were selected based on herbicide resistance and the presence of GFP. Gene knockdown was confirmed with qRT-PCR. The loop DNA fragment (S9 Table) is part of the *GUS* (β-glucuronidase) gene, and has no similarities to the genomes of the *Metarhizium* species investigated in this study.

### Assays of tolerance to abiotic stresses

Assays of UV tolerance were conducted as previously described [17]. Briefly, conidia were exposed to a weighted 312 nm (280–320 nm) UV-B wavelength at 0.2 J cm^-2^ in a Bio-Sun^++^ chamber (Vilber Lourmat, Marne-la-Vallée, France). Irradiated conidia were incubated at 26°C, and conidial germination was observed every 2 h using an inverted microscope (Leica, Germany). Tolerance to heat and cold stress was assayed by measuring conidial germination in 1/2 SDY every 2 h at 37°C and 15°C, respectively. The control temperature was 26°C. The relative germination inhibition of a given stressor on each strain was calculated as (Gc-Gt)/ Gc [23], where Gc and Gt denote the GT_50_ of the stressed and unstressed conidia, respectively. All assays were repeated three times with three replicates per repeat.

### Bioassays

Bioassays were conducted by applying a conidial suspension (1× 10^7^ conidia/ml) topically to the last instars of *G*. *mellonella* larvae (Ruiqingbait Co., Shanghai, China) as described [47]. Insect mortality was recorded daily. Bioassays were repeated three times with 40 insects per repeat.

For appressorial assays, conidia were inoculated on the hydrophobic surfaces of a Petri dish (Corning, USA) as previously described [22]. Turgor pressure in appressoria was measured as previously described [48]. Fluorescent staining of appressoria using Calcofluor Brightener White 2B was performed as previously described [14].

### Heterologous expression of *Pks1* and *Pks2* in *A*. *nidulans*

To construct the *Pks1* heterologous expression vector, SOE (splicing by overlapping extension)-PCR and yeast-based assembly approaches were used [49]. First, the constitutive *gpdA* promoter was introduced into the plasmid pYH-WA-pyrG as described [24] to form pYH-wA-pyrG-gpdA. Second, two PCR fragments with overlapping regions (250 bp), corresponding to the genomic region of the coding sequences of *Pks1* or *Pks2*, were amplified from *M*. *robertsii* genomic DNA. The two fragments and the *Nhe*I-digested pYH-WA-pyrG-gpdA were purified and transformed into *S*. *cerevisiae* BJ5464-NpgA using an S. c. EasyComp Transformation Kit (Invitrogen, USA). PCR was used to screen for yeast colonies containing the target plasmids. Target plasmids were isolated using a Zymoprep (D2001) Kit (Zymo Research, USA), and confirmed with restriction enzyme digestion and sequencing. Target plasmids were linearized with *Swa*I and transformed into the WT *A*. *nidulans* strain LO8030 to create transformants expressing *Pks1* or *Pks2*. The insertion of the *Pks1* or *Pks2* gene into the genome of *A*. *nidulans* was confirmed with PCR using a Taq Mix kit (Tiagen Biotech, China).

### Analytical and semi-preparation methods for SMs

To profile SMs, *A*. *nidulans* strains were cultivated at 25°C in 20 mL liquid LMM (lactose minimal medium). After 4 days of still cultivation, materials were extracted with ethyl acetate /methanol/acetic acid (89:10:1). The organic phase was dried in a vacuum and the residue was dissolved in 5 mg/mL methanol (MeOH) for HPLC analysis. Analytical HPLC was conducted with a flow rate of 1 mL/min using a linear gradient of 20% to 100% MeOH (0–20 min), 100% MeOH (20–25 min), and 20% MeOH (25–30 min). Analytical HPLC was performed on a Waters HPLC system (Waters e2695, Waters 2998, Photodiode Array Detector) using an ODS column (C18, 250 × 4.6 mm, YMC Pak, 5 μm).

For fermentation and SM semi-preparation, *A*. *nidulans* was cultivated in 10 L of liquid LMM media at 25°C for 4 d. Liquid culture and mycelia were extracted together three times with methanol/ethyl acetate (10:90). The organic phase was dried under reduced pressure, and the residue was then resuspended in MeOH׃hexane (1׃1) to remove all lipid components by discarding the hexane phase; this treatment was performed three times. The MeOH phase was dried under reduced pressure. The resulting residue was re-solubilized with MeOH and applied to an ODS column, and then eluted with MeOH using a gradient solvent system that ranged from 35% to 100% MeOH (250ml per gradient solvent). The target compound (Compound I) was detected in the 60% and 65% fractions, which were then combined and dried under reduced pressure. The residues were then solubilized with MeOH for a semi-preparation HPLC that was performed using an ODS column [HPLC (YMC-Pack ODS-A, 10 × 250 mm, 5 μm, 3 mL/min)]. The target peak for Compound I was solubilized in DMSO-*d*_6_ for NMR and LC-MS analysis. We performed LC-MS on an Agilent Accurate-Mass-QTOF LC/MS 6520 (Agilent Technologies, USA). NMR spectra (^1^H, ^13^C) were recorded on a Bruker Avance-500 spectrometer using tetramethylsilane as an internal standard. Chemical shifts were recorded as δ values.

### Construction of strains overexpressing *Pks2* and *Arp1*

The genomic regions corresponding to the coding sequences of *Pks2* and *Arp1* in *M*. *robertsii* were cloned using PCR with high fidelity Taq DNA polymerase (Toyobo, Japan). The genomic clone of the *Pks2* gene was inserted downstream of the constitutive promoter *Ptef* in the plasmid pPK2-Sur-Ptef [14], to produce the plasmid pPK2-Sur-Ptef-Pks2 with the herbicide resistant *Sur* gene. The genomic clone of the *Arp1* gene was inserted downstream of the constitutive promoter *Ptef* in the plasmid pPK2-Bar-Ptef [14], to produce the plasmid pPK2-Bar-Ptef-Arp1 with the herbicide resistant *Bar* gene. pPK2-sur-Ptef-Pks2 was then transferred into *A*. *tumefaciens*, and transformed into either the *M*. *robertsii Pks1-*deletion mutant [17] or wild-type *M*. *album*. Overexpression of *Pks2* in *M*. *robertsii* was confirmed with qRT-PCR. The pPK2-Bar-Ptef-Arp1 plasmid was transformed into the *M*. *album* strain expressing the *Pks2* gene to produce a strain expressing *Pks2* and *Arp1* simultaneously. The expression of both *Pks2* and *Arp1* in *M*. *album* was confirmed with RT-PCR.

### Accession numbers

*M*. *acridum Pks1*: MG385100; *M*. *album Pks1*: MG385101.

## Supporting information

S1 Fig Manual annotation of the *Pks1* gene in *M*. *acridum* and *M*. *album*. (**A**) A schematic diagram showing the reannotation of the *Pks1* genes in *M*. *acridum* and *M*. *album*. Wrong annotation (the boxed area) of the genomic region (MAA_05385 in *M*. *acridum*; MAM_08215 in *M*. *album*) corresponding to EthD and PKS1 by NCBI is shown at the bottom; note: a gene containing EthD and PKS1 is annotated from this region. At the top is shown the new annotation of the genomic region corresponding to *EthD* and *Pks1*; note: two genes *(EthD* and *Pks1*) are annotated from this region. PS1, PS2, PS3 and PS4 brackets show the relative positions of the amplified mRNA fragments representing *EthD*, *Pks1*, the fragment spanning *EthD* and *Pks1*, and the region corresponding to N-terminus of the newly annotated *Pks1*, respectively. The “ATG” is the start codon of the newly annotated *Pks1* gene. (**B**) RT-PCR amplification of the PS1, PS2 and PS3 regions (shown in A) from *M*. *acridum* and *M*. *album*. Note: no PCR products were obtained from PS1. (**C**) RT-PCR amplification of the PS4 region (shown in A) from *M*. *acridum* (1) and *M*. *album* (2).
(PDF)

S2 Fig Phylogenetic analysis of domains in the PKSs analyzed in Fig 1. (**A**) AT domain; (**B**) PP-binding domain, (**C**) PS-DH domain, (**D**) TE domain. The Genbank accession number for each full-length amino acid sequence of each PKS is shown after the species name. The clade containing *Metarhizium* PKS1s is highlighted in pink, PKS2s highlighted in red. Numbers at nodes represent the bootstrap values of Maximum Likelihood (left), Neighbor-Joining (middle), and the Bayesian posterior probabilities (right). A hyphen (-) indicates no support value in the corresponding method. The scale bar corresponds to the estimated number of amino acid substitutions per site.
(PDF)

S3 Fig The clades (a to e) assigned for comparison of topologies of alternative (constrained) trees with the obtained tree. Shown is the obtained tree (Fig 1A). The clade *a* in pink is the PKS1 clade, and the clade c in red is the PKS2 clade. The results of the topology comparison are shown in S2 Table.
(PDF)

S4 Fig Species tree of the 31 fungal species analyzed in Fig 1. The species tree was constructed using the concatenated alignment (S2 Dataset) of the 20 best scoring single-copy genes (S8 Table). Numbers at nodes represent the bootstrap values of Maximum Likelihood (left), Neighbor-Joining (middle), and the Bayesian posterior probabilities (right). A hyphen (-) indicates no support value in the corresponding method.
(PDF)

S5 Fig Estimation of gene duplication and loss events of the *Pks* genes in the fungal species shown in Fig 1A by reconciling the rearranged *Pks* gene ML tree (Fig 2A) with the species tree (S4 Fig) using NOTUNG with a duplication-loss (DL) model (1.5 for a duplication, 0.0 for a conditional duplication and 1.0 for a loss.). Red circles indicate duplication events inferred by the reconciliation. Blue notes near the nodes denote the internal node species name. Gray branches (branch name with LOST): inferred loss events.
(PDF)

S6 Fig Estimation of gene duplication and loss events of the *Pks* genes in the fungal species shown in Fig 1A by reconciling the raw *Pks* gene’s ML tree (Fig 1A) with the species tree (S4 Fig) using NOTUNG with a duplication-loss (DL) model (1.5 for a duplication, 0.0 for a conditional duplication and 1.0 for a loss.). Red circles indicate duplication events inferred by the reconciliation. Blue notes near the nodes denote the internal node species name. Gray branches (branch name with LOST): inferred loss events.
(PDF)

S7 Fig Phylogenetic analysis of KS domains in the PKSs analyzed in Fig 1A and additional 18 PKSs in *M*. *robertsii*. The Genbank accession number for each full-length amino acid sequence of each PKS is shown after the species name. The clade containing *Metarhizium*’s PKS1s is highlighted in pink, PKS2s highlighted in red and additional *M*. *robertsii* PKSs in blue. Numbers at nodes represent the bootstrap values of Maximum Likelihood (left), Neighbor-Joining (middle), and the Bayesian posterior probabilities (right). A hyphen (-) indicates no support value in the corresponding method. The scale bar corresponds to the estimated number of amino acid substitutions per site.
(PDF)

S8 Fig Phylogenetic analyses of Arp1 (A), Arp2 (B) and Abr2 (C) in *Metarhizium* and other fungi that have gene clusters similar to *Pks1-gc* or *Pks2-gc*. Numbers at nodes represent bootstrap values of Maximum Likelihood (left), Neighbor-Joining (middle) and Bayesian posterior probabilities (right). Hyphen (-) indicates no support value in the corresponding method. The scale bar corresponds to the estimated number of amino acid substitutions per site.
(PDF)

S9 Fig Ka/Ks values of each *Pks1* and *Pks2* gene in *Metarhizium* species. (**A**) *Pks1*s. (**B**) *Pks2*s.
(PDF)

S10 Fig Construction of knockout (KO) or knockdown (KD) mutants of *Pks1* and *Pks2* genes in *Metarhizium*. (**A**) A schematic diagram of gene disruption based on homologous recombination showing a map of a disruption plasmid and its relative position in the fungal genome. Note: only around the region (~1.2kb) corresponding to the N-terminus of a *Pks* gene was deleted. (**B**) Confirmation of knocking out *Pks1* genes in *M*. *anisopliae*, *M*. *brunneum*, *M*. *guizhouense* and *M*. *acridum* and *Pks2* gene in *M*. *robertsii*. D1, D2 and D3 represent three independent isolates of a *Pks* KO mutant, and WT is the wild-type strain. Upper panel: PCR conducted with the primers Bar-up and the confirmation primer CF-2 (the relative position of all primers are shown in A). PCR products can be obtained only from the KO mutants. Lower panel: PCR was conducted with primers CF-1 and CF-2; PCR products can be obtained in the WT strain but not in the KO mutants. The DNA ladder (DL 10004) was purchased from Generay (Shanghai, China). (**C**) qRT-PCR confirmation of knocking down *Pks1* genes in *M*. *majus* (left panel) and *M*. *album* (right panel). The expression level in WT was set to 1. #1, 2, and 3: three independent isolates of a gene knocking down mutant. The qRT-PCR analyses were repeated three times with three replicates per repeat. Data are expressed as the mean ± SE. Values with different letters are significantly different (Student’s *t* test, *P* < 0.01).
(PDF)

S11 Fig Conidial pigmentation of two of three independent isolates of a *Pks1* gene’s KD (Knock down) or KO (knock out) mutant. One of the three isolates for each mutant is shown in Fig 6. Scale bars represent 1 mm in *M*. *album* and 10 mm in *M*. *anisopliae*, *M*. *brunneum*, *M*. *guizhouense*, *M*. *majus*, *M*. *acridum* and *M*. *album*.
(PDF)

S12 Fig Conidial pigmentation of WT and three independent isolates of the *Pks2* KO (knock out) mutant in *M*. *robertsii*. Scale bars represent 10 mm. Note: no difference in conidial pigments in seen between the three independent isolates of the mutant and the WT.
(PDF)

S13 Fig Assays of appressoria of WT (the wild-type strain of *M*. *robertsii*) and three independent isolates (*ΔPks2* #1, *ΔPks2* #2, and *ΔPks2* #3) of *M*. *robertsii*’s *Pks2* KO mutant *ΔPks2*. (**A**) Collapse rates of appressoria in the PEG8000 solution [80% (w/v)]. The assays were repeated three times with three replicates per repeat. Data are expressed as the mean ± SE. Values with different letters are significantly different (*P* < 0.05, Tukey’s test in One-way ANOVA). (**B**) Appressoria (against a hydrophobic plastic surface) stained with Calcofluor Brightener White 2B. AP: appressorium; CO: conidium. Left panel: Bright field microscopy; Right panel: Fluorescence microscopy. Note: no difference in fluorescent intensity between WT and *ΔPks2*. Images are representative of at least three independent experiments for each condition. Scale bars represent 10μm.
(PDF)

S14 Fig RT-PCR confirmation of expression of *M*. *robertsii*’s *Pks2* (Left panel) and *Arp1* (Right panel) in *M*. *album*. M: DNA ladder (Genray, Shanghai); 1: The wild-type *M*. *album*; 2, 3, 4: three independent *M*. *album* transformants expressing *M*. *robertsii*’s *Pks2* and *Arp1*.
(PDF)

S15 Fig Confirmation of the insertions of *M*. *robertsii*’s *Pks1* and *Pks2* into the genome of *A*. *nidulans* strain LO8030. PCR reactions were conducted with primers MAA_Pks1_RT_F/R for *Pks1* and MAA_Pks2_RT_F/R for *Pks2* (see S9 Table for information about the primers). T1 to T5 represents five independent transformants expressing *Pks1* or *Pks2*; CK: the positive control (the DNA template for PCR was *M*. *robertsii*’s genomic DNA). M: DNA Ladder (Tiangen Biotech, China).
(PDF)

S16 Fig LC-MS analysis of Compound I (shown in Fig 8) from the *A*. *nidulans* transformant expressing *Pks1*. This figure is supplemental to Fig 8C. Molecular weight of Compound I was detected by LC-MS analysis at m/z 315 [M+H]+, and 651 [M+Na]+.
(PDF)

S17 Fig ^1^H NMR spectrum of Compound I (shown in Fig 8) in DMSO-*d*_*6*_. (PDF)

S18 Fig ^13^C NMR spectrum of Compound I (shown in Fig 8) in DMSO-*d*_*6*_. (PDF)

S19 Fig *Pks2* cannot complement the *Pks1* KO mutant (*ΔPks1*) in *M*. *robertsii*. (**A**) qRT-PCR confirmation of overexpression of *Pks2* in *ΔPks1*. *ΔPks1*-*Pks2*^*OE*^: *ΔPks1* with *Pks2* overexpressed. 1, 2, 3: three independent isolates of *ΔPks1*-*Pks2*^*OE*^. The expression level in *ΔPks1* is set to 1. Values with different letters are significantly different (*P <* 0.05, Tukey’s test in One-way ANOVA). All assays were repeated three times with three replicates per repeat. (**B**) Colony morphology of *ΔPks1* and the three independent isolates (*ΔPks1*-*Pks2*^*OE*^*-*1, -2 and -3) of *ΔPks1*-*Pks2*^*OE*^. Note: all strains produce red conidia. Pictures were taken 18 days after inoculation of 5μl of conidial suspension (4×10^7^ conidia/ml) on a PDA plate. Scale bars represent 10mm. (**C**) HPLC detection of Compound I *ΔPks1* and the three independent isolates (*ΔPks1*-*Pks2*^*OE*^*-*1, -2 and -3) of *ΔPks1*-*Pks2*^*OE*^. Standard: the purified Compound I, indicated by arrow, from the *A*. *nidulans* transformant TYPZ26.1.
(PDF)

S1 Table Information about proteins and their domain structures used in the analyses conducted in Fig 1. (PDF)

S2 Table Statistics of comparison of topologies of the constrained trees (S3 Fig) with the obtained tree (Fig 1). (PDF)

S3 Table GT_50_ (Time taken for 50% of conidia to germinate) values of three independent isolates of a *Pks1* mutant in seven *Metarhizium* species and their respective wild-type strains (WT) under optimal conditions (grown at 26°C in 1/2 SDY). Within each row (species), values appended by different letters are significantly different (*P* < 0.05, Tukey’s test in One-way ANOVA). All assays were repeated three times with three replicates per repeat.
(PDF)

S4 Table Relative germination rates of two of three independent isolates (#2 and #3) of a *Pks1* mutant in seven *Metarhizium* species under three abiotic stresses. All assays were repeated three times with three replicates per repeat.
(PDF)

S5 Table Relative germination rates of three independent isolates of the *M*. *robertsii Pks2* KO mutant under three abiotic stresses. All assays were repeated three times with three replicates per repeat. Within each row (strains), values appended by different letters are significantly different (*P* < 0.05, Tukey’s test in One-way ANOVA).
(PDF)

S6 Table NMR spectroscopic data for Compound I (1-acetyl-2,4,6,8-tetrahydroxy-9,10-anthraquinone) in DMSO-*d*_*6*_. (PDF)

S7 Table Plasmids and fungal strains used in this study. (PDF)

S8 Table Genbank accession numbers of the 20 genes in the 31 fungal species shown in Fig 1A for constructing a species phylogenetic tree (shown in S4 Fig). (PDF)

S9 Table Primers used in this study. (PDF)

S1 Dataset The .nwk files for the CONSEL assays. (ZIP)

S2 Dataset The concatenated alignment of 20 genes for constructing species tree. (FASTA)

S3 Dataset NOTUNG assays using DL or DTL model with different parameter combinations. (PDF)

S4 Dataset Alignment of PKS domains. (PDF)

S5 Dataset Logos and sequences of overrepresented motifs in the promoters of the *Pks1* and *Pks2* genes in *Metarhizium*. (PDF)
