# Supplementary material for: Duplication of a Pks gene cluster and subsequent functional diversification facilitate environmental adaptation in Metarhizium species
Source: PLoS Genet. 2018 Jun 29;14(6):e1007472. doi: 10.1371/journal.pgen.1007472 (PMC6042797; doi:10.1371/journal.pgen.1007472)
Supplement: S1 Fig — (A) A schematic diagram showing the reannotation of the Pks1 genes in M. acridum and M. album. Wrong annotation (the boxed area) of the genomic region (MAA_05385 in M. acridum; MAM_08215 in M. album) corresponding to EthD and PKS1 by NCBI is shown at the bottom; note: a gene containing EthD and PKS1 is annotated from this region. At the top is shown the new annotation of the genomic region corresponding to EthD and Pks1; note: two genes (EthD and Pks1) are annotated from this region. PS1, PS2, PS3 and PS4 brackets show the relative positions of the amplified mRNA fragments representing EthD, Pks1, the fragment spanning EthD and Pks1, and the region corresponding to N-terminus of the newly annotated Pks1, respectively. The “ATG” is the start codon of the newly annotated Pks1 gene. (B) RT-PCR amplification of the PS1, PS2 and PS3 regions (shown in A) from M. acridum and M. album. Note: no PCR products were obtained from PS1. (C) RT-PCR amplification of the PS4 region (shown in A) from M. acridum (1) and M. album (2). (PDF) [file pgen.1007472.s001.pdf]

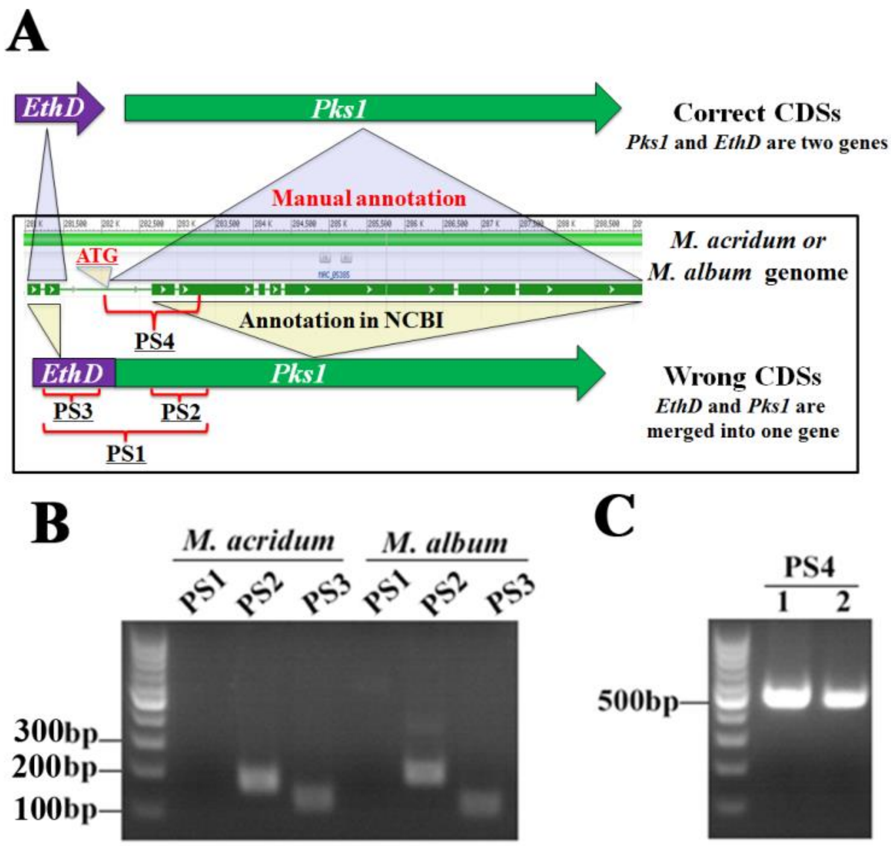

S1 Fig: Manual annotation of the *Pks1* gene in *M. acridum* and *M. album*. (**A**) A schematic diagram showing the reannotation of the *Pks1* genes in *M. acridum* and *M. album*. Wrong annotation (the boxed area) of the genomic region (MAA_05385 in *M. acridum*; MAM_08215 in *M. album*) corresponding to EthD and PKS1 by NCBI is shown at the bottom; note: a gene containing EthD and PKS1 is annotated from this region. At the top is shown the new annotation of the genomic region corresponding to *EthD* and *Pks1*; note: two genes *(EthD* and *Pks1*) are annotated from this region. PS1, PS2, PS3 and PS4 brackets show the relative positions of the amplified mRNA fragments representing *EthD*, *Pks1*, the fragment spanning *EthD* and *Pks1*, and the region corresponding to N-terminus of the newly annotated *Pks1*, respectively. The “ATG” is the start codon of the newly annotated *Pks1* gene. (**B**) RT-PCR amplification of the PS1, PS2 and PS3 regions (shown in A) from *M. acridum* and *M. album*. Note: no PCR products were obtained from PS1. (**C**) RT-PCR amplification of the PS4 region (shown in A) from *M. acridum* (1) and *M. album* (2).
