# Supplementary material for: Duplication of a Pks gene cluster and subsequent functional diversification facilitate environmental adaptation in Metarhizium species
Source: PLoS Genet. 2018 Jun 29;14(6):e1007472. doi: 10.1371/journal.pgen.1007472 (PMC6042797; doi:10.1371/journal.pgen.1007472)
Supplement: S2 Fig — (A) AT domain; (B) PP-binding domain, (C) PS-DH domain, (D) TE domain. The Genbank accession number for each full-length amino acid sequence of each PKS is shown after the species name. The clade containing Metarhizium PKS1s is highlighted in pink, PKS2s highlighted in red. Numbers at nodes represent the bootstrap values of Maximum Likelihood (left), Neighbor-Joining (middle), and the Bayesian posterior probabilities (right). A hyphen (-) indicates no support value in the corresponding method. The scale bar corresponds to the estimated number of amino acid substitutions per site. (PDF) [file pgen.1007472.s002.pdf]

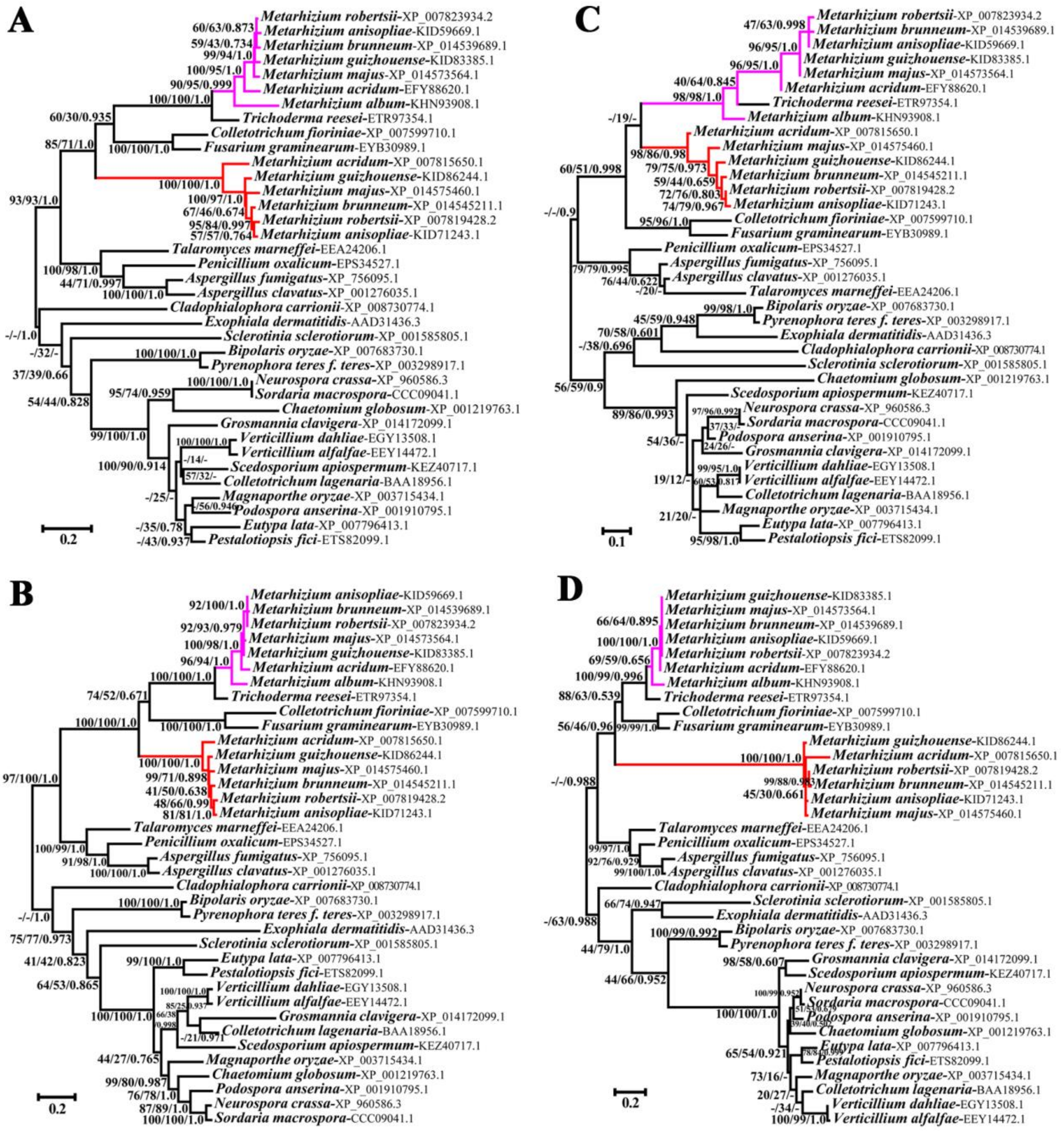

S2 Fig: Phylogenetic analysis of domains in the PKSs analyzed in Fig 1. (**A**) AT domain; (**B**) PP-binding domain, (**C**) PS-DH domain, (**D**) TE domain. The Genbank accession number for each full-length amino acid sequence of each PKS is shown after the species name. The clade containing *Metarhizium* PKS1s is highlighted in pink, PKS2s highlighted in red. Numbers at nodes represent the bootstrap values of Maximum Likelihood (left), Neighbor-Joining (middle), and the Bayesian posterior probabilities (right). A hyphen (-) indicates no support value in the corresponding method. The scale bar corresponds to the estimated number of amino acid substitutions per site.
