# Supplementary material for: Duplication of a Pks gene cluster and subsequent functional diversification facilitate environmental adaptation in Metarhizium species
Source: PLoS Genet. 2018 Jun 29;14(6):e1007472. doi: 10.1371/journal.pgen.1007472 (PMC6042797; doi:10.1371/journal.pgen.1007472)
Supplement: S3 Fig — Shown is the obtained tree (Fig 1A). The clade a in pink is the PKS1 clade, and the clade c in red is the PKS2 clade. The results of the topology comparison are shown in S2 Table. (PDF) [file pgen.1007472.s003.pdf]

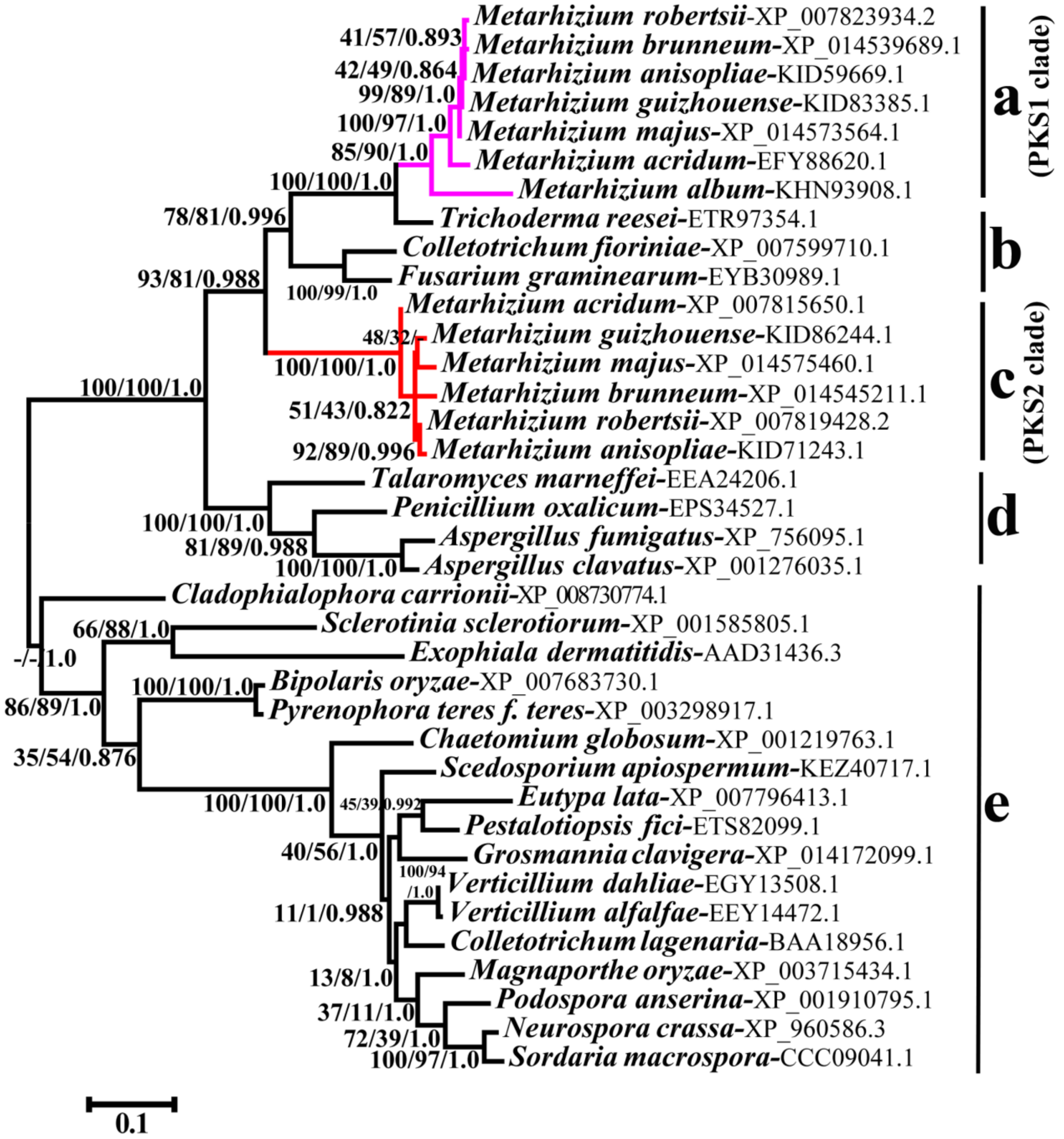

S3 Fig: The clades (a to e) assigned for comparison of topologies of alternative (constrained) trees with the obtained tree. Shown is the obtained tree (Fig 1A). The clade *a* in pink is the PKS1 clade, and the clade c in red is the PKS2 clade. The results of the topology comparison are shown in S2 Table.
