# Supplementary material for: Duplication of a Pks gene cluster and subsequent functional diversification facilitate environmental adaptation in Metarhizium species
Source: PLoS Genet. 2018 Jun 29;14(6):e1007472. doi: 10.1371/journal.pgen.1007472 (PMC6042797; doi:10.1371/journal.pgen.1007472)
Supplement: S4 Fig — The species tree was constructed using the concatenated alignment (S2 Dataset) of the 20 best scoring single-copy genes (S8 Table). Numbers at nodes represent the bootstrap values of Maximum Likelihood (left), Neighbor-Joining (middle), and the Bayesian posterior probabilities (right). A hyphen (-) indicates no support value in the corresponding method. (PDF) [file pgen.1007472.s004.pdf]

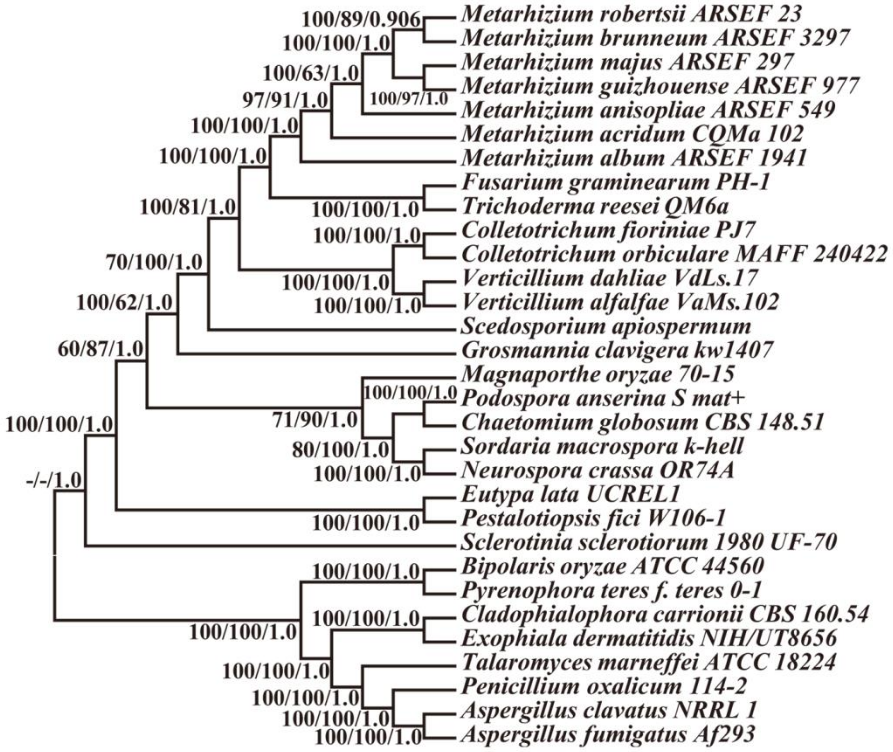

S4 Fig: Species tree of the 31 fungal species analyzed in Fig. 1. The species tree was constructed using the concatenated alignment (S2 Dataset) of the 20 best scoring single-copy genes (S7 Table). Numbers at nodes represent the bootstrap values of Maximum Likelihood (left), Neighbor-Joining (middle), and the Bayesian posterior probabilities (right). A hyphen (-) indicates no support value in the corresponding method.
