# Supplementary material for: Duplication of a Pks gene cluster and subsequent functional diversification facilitate environmental adaptation in Metarhizium species
Source: PLoS Genet. 2018 Jun 29;14(6):e1007472. doi: 10.1371/journal.pgen.1007472 (PMC6042797; doi:10.1371/journal.pgen.1007472)
Supplement: S6 Fig — Red circles indicate duplication events inferred by the reconciliation. Blue notes near the nodes denote the internal node species name. Gray branches (branch name with LOST): inferred loss events. (PDF) [file pgen.1007472.s006.pdf]

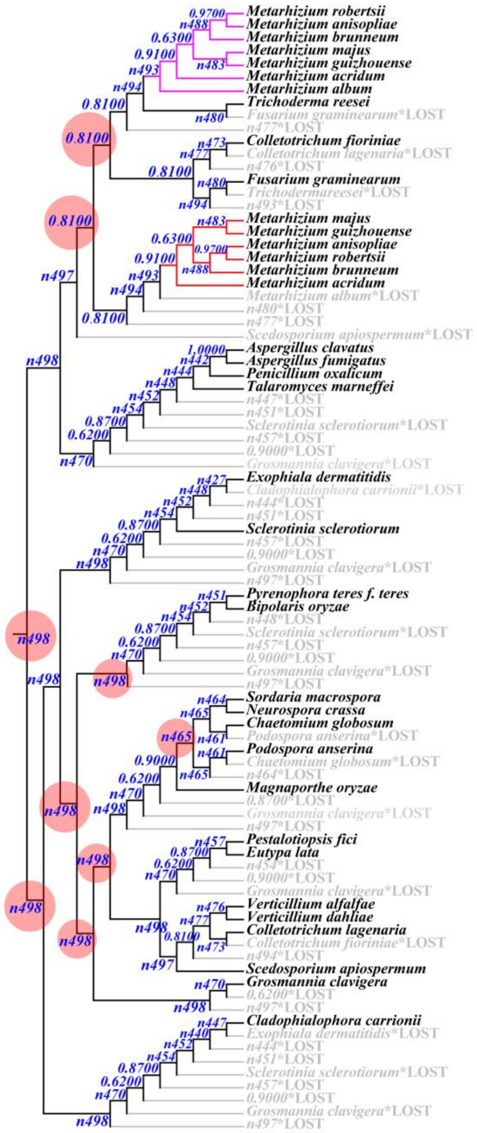

S6 Fig: Estimation of gene duplication and loss events of the *Pks* genes in the fungal species shown in Fig 1A by reconciling the raw *Pks* gene's ML tree (Fig 1A) with the species tree (S4 Fig) using NOTUNG with a duplication-loss (DL) model with default parameters (1.5 for duplication and 1.0 for loss), and six other parameter combinations (1.0 for duplication, 1.0 for loss; 2.0 for duplication, 1.0 for loss; 1.5 for duplication, 0.5 for loss; 1.5 for duplication, 1.5 for loss; 1.5 for duplication, 0.5 for loss; 1.5 for duplication, 1.5 for loss). The same result was produced from all assays, and the one with default parameters was presented here as a representative. Red circles indicate duplication events inferred by the reconciliation. Blue notes near the nodes denote the internal node species name. Gray branches (branch name with LOST): inferred loss events.
