# Supplementary material for: Duplication of a Pks gene cluster and subsequent functional diversification facilitate environmental adaptation in Metarhizium species
Source: PLoS Genet. 2018 Jun 29;14(6):e1007472. doi: 10.1371/journal.pgen.1007472 (PMC6042797; doi:10.1371/journal.pgen.1007472)
Supplement: S8 Fig — Numbers at nodes represent bootstrap values of Maximum Likelihood (left), Neighbor-Joining (middle) and Bayesian posterior probabilities (right). Hyphen (-) indicates no support value in the corresponding method. The scale bar corresponds to the estimated number of amino acid substitutions per site. (PDF) [file pgen.1007472.s008.pdf]

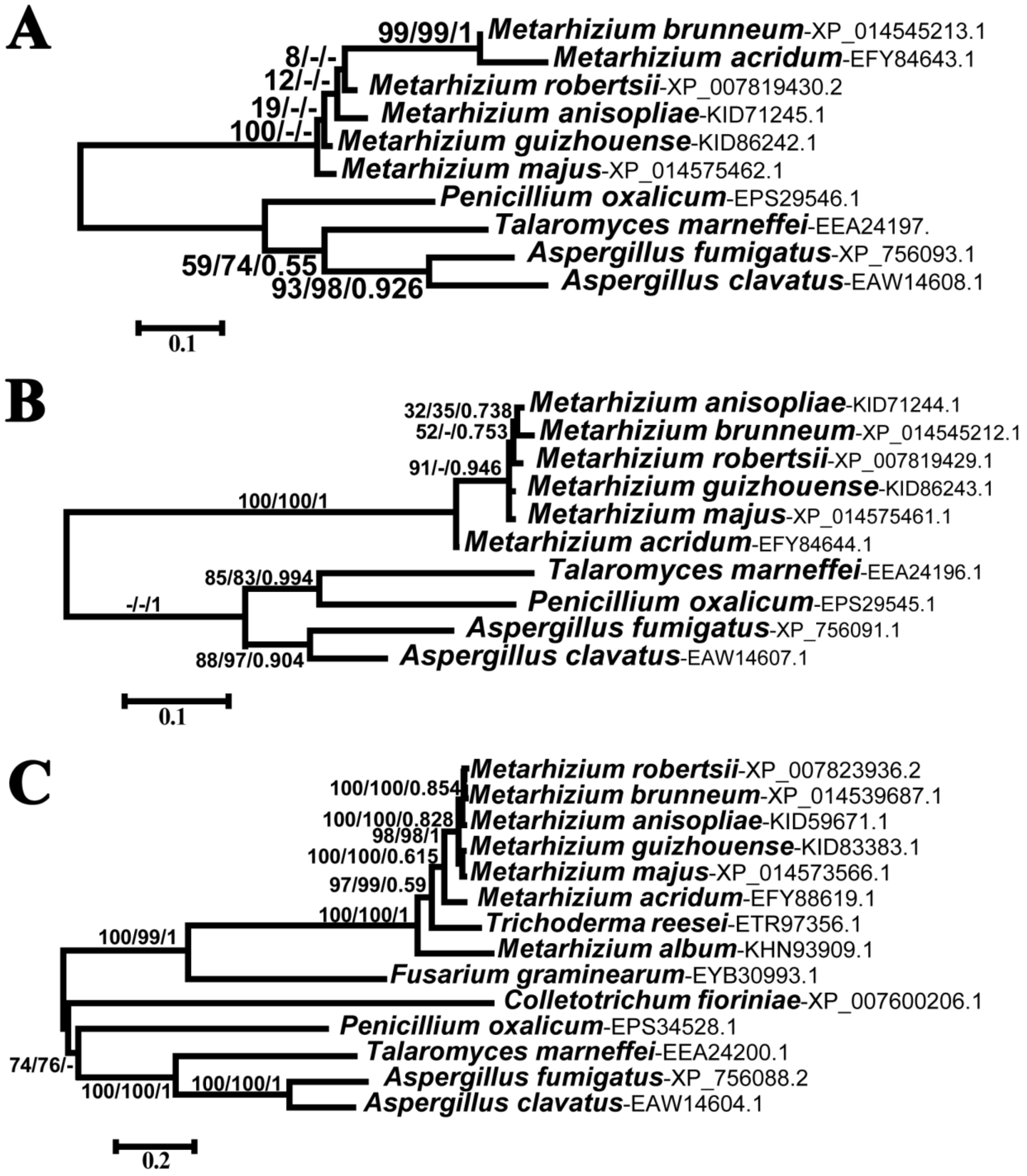

S8 Fig: Phylogenetic analyses of Arp1 (**A**), Arp2 (**B**) and Abr2 (**C**) in *Metarhizium* and other fungi that have gene clusters similar to *Pks1-gc* or *Pks2-gc*. Numbers at nodes represent bootstrap values of Maximum Likelihood (left), Neighbor-Joining (middle) and Bayesian posterior probabilities (right). Hyphen (-) indicates no support value in the corresponding method. The scale bar corresponds to the estimated number of amino acid substitutions per site.
