# Supplementary figures and images for: Duplication of a Pks gene cluster and subsequent functional diversification facilitate environmental adaptation in Metarhizium species
Source: PLoS Genet. 2018 Jun 29;14(6):e1007472. doi: 10.1371/journal.pgen.1007472 (PMC6042797; doi:10.1371/journal.pgen.1007472)

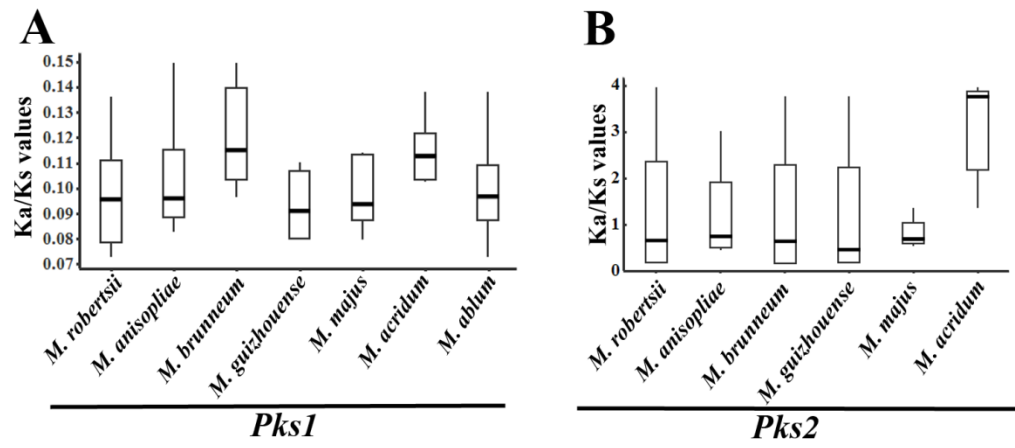

**S9 Fig:** Ka/Ks values of each *Pks1* and *Pks2* gene in *Metarhizium* species. **(A)** *Pks1*s.  
**(B)** *Pks2*s.

Supplement: S9 Fig — (A) Pks1s. (B) Pks2s. (PDF) [file pgen.1007472.s009.pdf]

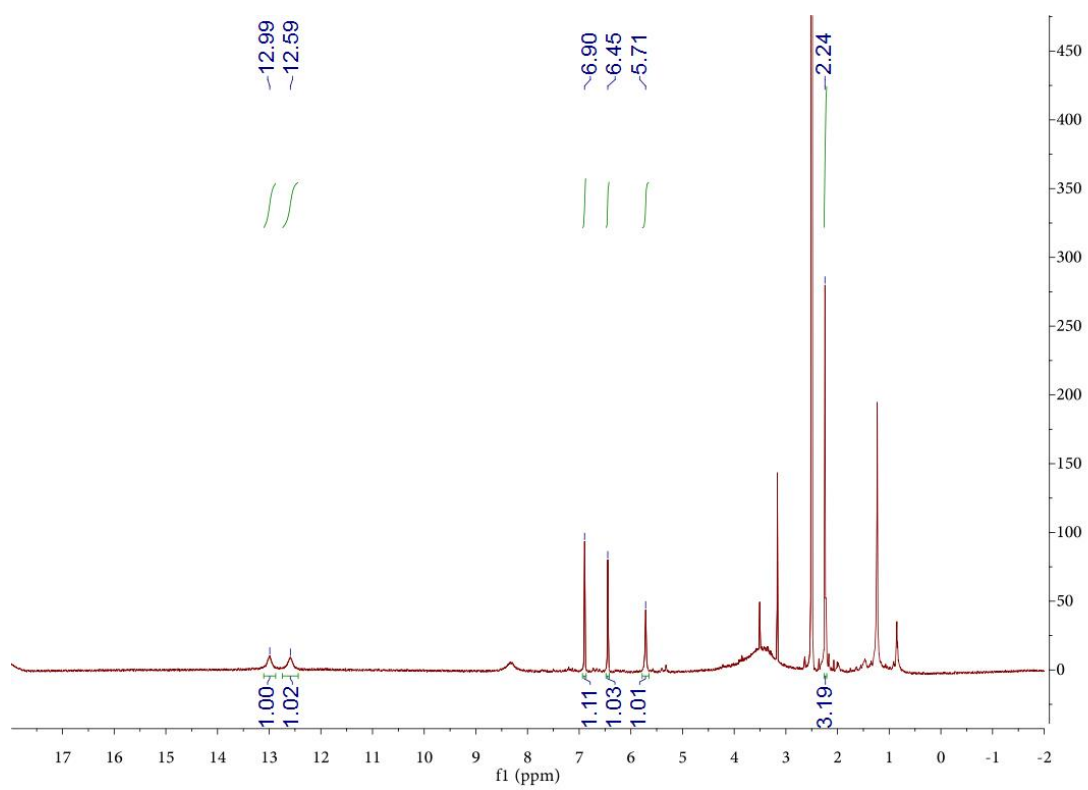

**S17 Fig:** <sup>1</sup>H NMR spectrum of Compound I (shown in Fig 8) in DMSO-*d*<sub>6</sub>.

Supplement: S17 Fig — (PDF) [file pgen.1007472.s017.pdf]

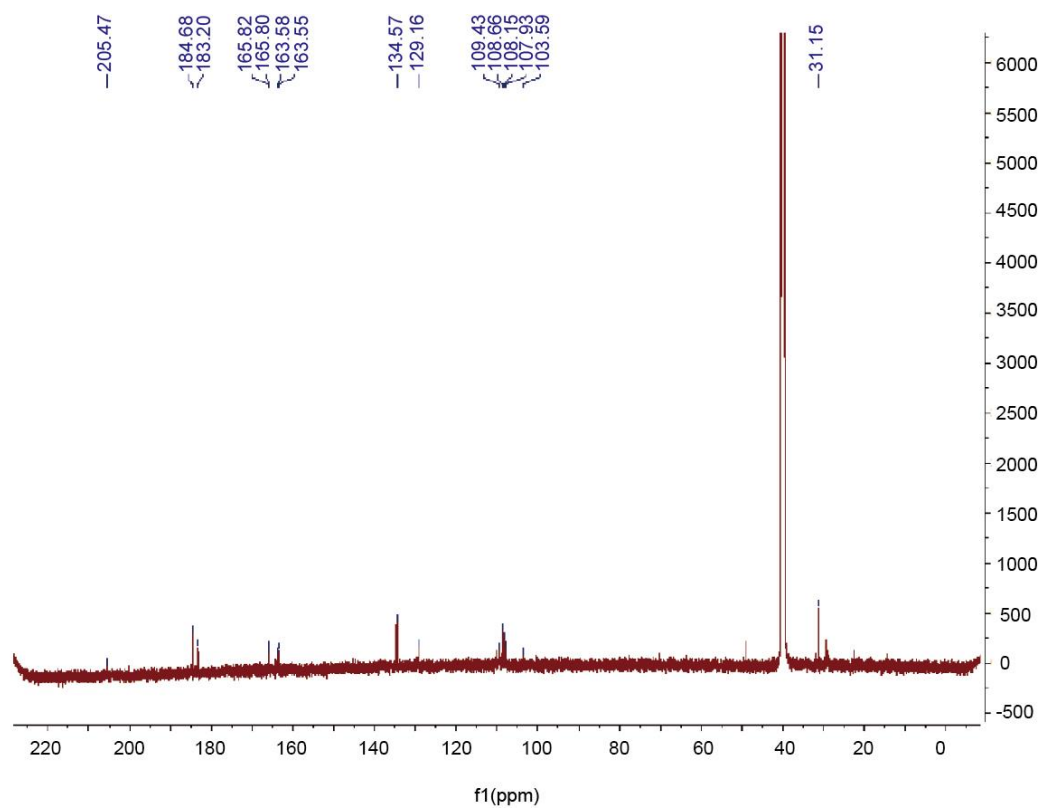

**S18 Fig:**  $^{13}\text{C}$  NMR spectrum of Compound I (shown in Fig 8) in  $\text{DMSO-}d_6$ .

Supplement: S18 Fig — (PDF) [file pgen.1007472.s018.pdf]
