# Supplementary material for: Duplication of a Pks gene cluster and subsequent functional diversification facilitate environmental adaptation in Metarhizium species
Source: PLoS Genet. 2018 Jun 29;14(6):e1007472. doi: 10.1371/journal.pgen.1007472 (PMC6042797; doi:10.1371/journal.pgen.1007472)
Supplement: S10 Fig — (A) A schematic diagram of gene disruption based on homologous recombination showing a map of a disruption plasmid and its relative position in the fungal genome. Note: only around the region (~1.2kb) corresponding to the N-terminus of a Pks gene was deleted. (B) Confirmation of knocking out Pks1 genes in M. anisopliae, M. brunneum, M. guizhouense and M. acridum and Pks2 gene in M. robertsii. D1, D2 and D3 represent three independent isolates of a Pks KO mutant, and WT is the wild-type strain. Upper panel: PCR conducted with the primers Bar-up and the confirmation primer CF-2 (the relative position of all primers are shown in A). PCR products can be obtained only from the KO mutants. Lower panel: PCR was conducted with primers CF-1 and CF-2; PCR products can be obtained in the WT strain but not in the KO mutants. The DNA ladder (DL 10004) was purchased from Generay (Shanghai, China). (C) qRT-PCR confirmation of knocking down Pks1 genes in M. majus (left panel) and M. album (right panel). The expression level in WT was set to 1. #1, 2, and 3: three independent isolates of a gene knocking down mutant. The qRT-PCR analyses were repeated three times with three replicates per repeat. Data are expressed as the mean ± SE. Values with different letters are significantly different (Student’s t test, P < 0.01). (PDF) [file pgen.1007472.s010.pdf]

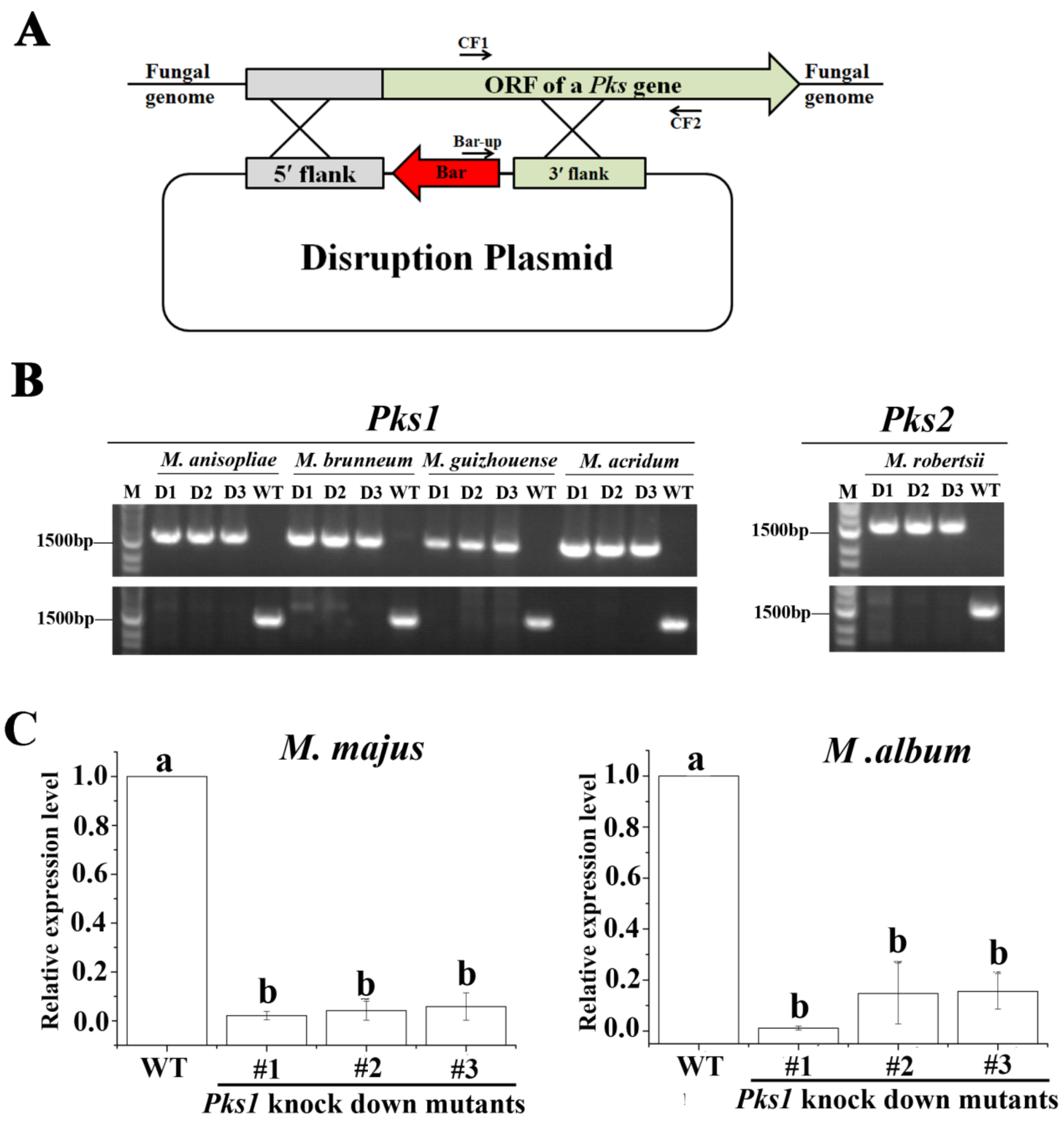

S10 Fig: Construction of knockout (KO) or knockdown (KD) mutants of *Pks1* and *Pks2* genes in *Metarhizium*. (**A**) A schematic diagram of gene disruption based on homologous recombination showing a map of a disruption plasmid and its relative position in the fungal genome. Note: only around the region (~1.2kb) corresponding to the N-terminus of a *Pks* gene was deleted. (**B**) Confirmation of knocking out *Pks1* genes in *M. anisopliae, M. brunneum, M. guizhouense* and *M. acridum* and *Pks2* gene in *M. robertsii*. D1, D2 and D3 represent three independent isolates of a *Pks* KO mutant, and WT is the wild-type strain. Upper panel: PCR conducted with the primers Bar-up and the confirmation primer CF-2 (the relative position of all primers are shown in A). PCR products can be obtained only from the KO mutants. Lower panel:

PCR was conducted with primers CF-1 and CF-2; PCR products can be obtained in the WT strain but not in the KO mutants. The DNA ladder (DL 10004) was purchased from Generay (Shanghai, China). (**C**) qRT-PCR confirmation of knocking down *Pks1* genes in *M. majus* (left panel) and *M. album* (right panel). The expression level in WT was set to 1. #1, 2, and 3: three independent isolates of a gene knocking down mutant. The qRT-PCR analyses were repeated three times with three replicates per repeat. Data are expressed as the mean ± SE. Values with different letters are significantly different (Student's *t* test, $P < 0.01$).
