# Supplementary material for: Duplication of a Pks gene cluster and subsequent functional diversification facilitate environmental adaptation in Metarhizium species
Source: PLoS Genet. 2018 Jun 29;14(6):e1007472. doi: 10.1371/journal.pgen.1007472 (PMC6042797; doi:10.1371/journal.pgen.1007472)
Supplement: S11 Fig — One of the three isolates for each mutant is shown in Fig 6. Scale bars represent 1 mm in M. album and 10 mm in M. anisopliae, M. brunneum, M. guizhouense, M. majus, M. acridum and M. album. (PDF) [file pgen.1007472.s011.pdf]

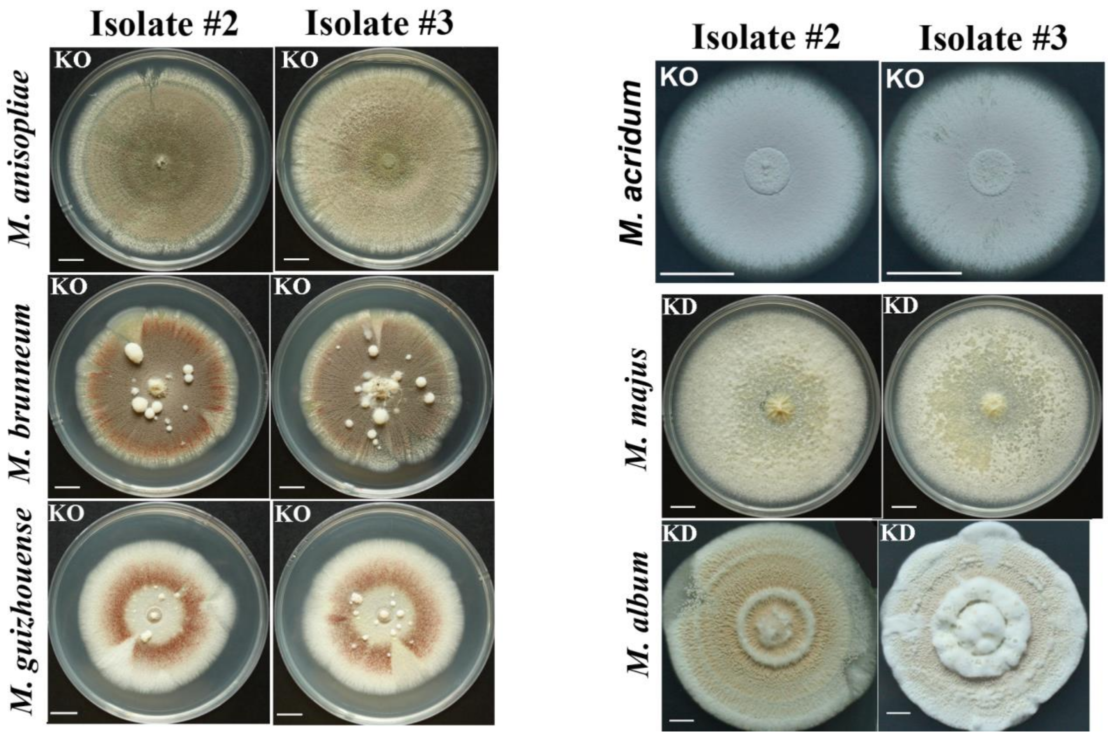


**S11 Fig**: Conidial pigmentation of two of three independent isolates of a *Pks1* gene's KD (Knock down) or KO (knock out) mutant. One of the three isolates for each mutant is shown in Fig. 6. Scale bars represent 1 mm in *M. album* and 10 mm in *M. anisopliae*, *M. brunneum*, *M. guizhouense*, *M. majus*, *M. acridum* and *M. album*.
