# Supplementary material for: Duplication of a Pks gene cluster and subsequent functional diversification facilitate environmental adaptation in Metarhizium species
Source: PLoS Genet. 2018 Jun 29;14(6):e1007472. doi: 10.1371/journal.pgen.1007472 (PMC6042797; doi:10.1371/journal.pgen.1007472)
Supplement: S12 Fig — Scale bars represent 10 mm. Note: no difference in conidial pigments in seen between the three independent isolates of the mutant and the WT. (PDF) [file pgen.1007472.s012.pdf]

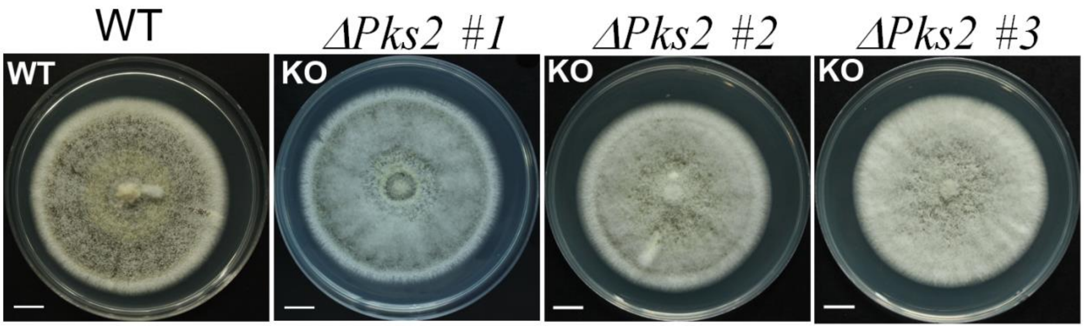

S12 Fig: Conidial pigmentation of WT and three independent isolates of the *Pks2* KO (knock out) mutant in *M. robertsii.* Scale bars represent 10 mm. Note: no difference in conidial pigments in seen between the three independent isolates of the mutant and the WT.
