# Supplementary material for: Duplication of a Pks gene cluster and subsequent functional diversification facilitate environmental adaptation in Metarhizium species
Source: PLoS Genet. 2018 Jun 29;14(6):e1007472. doi: 10.1371/journal.pgen.1007472 (PMC6042797; doi:10.1371/journal.pgen.1007472)
Supplement: S13 Fig — (A) Collapse rates of appressoria in the PEG8000 solution [80% (w/v)]. The assays were repeated three times with three replicates per repeat. Data are expressed as the mean ± SE. Values with different letters are significantly different (P < 0.05, Tukey’s test in One-way ANOVA). (B) Appressoria (against a hydrophobic plastic surface) stained with Calcofluor Brightener White 2B. AP: appressorium; CO: conidium. Left panel: Bright field microscopy; Right panel: Fluorescence microscopy. Note: no difference in fluorescent intensity between WT and ΔPks2. Images are representative of at least three independent experiments for each condition. Scale bars represent 10μm. (PDF) [file pgen.1007472.s013.pdf]

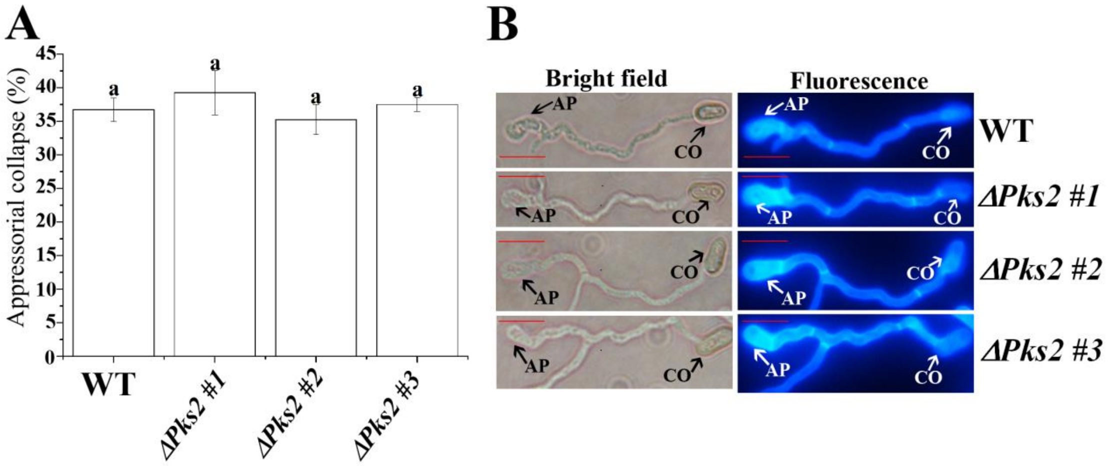

S13 Fig: Assays of appressoria of WT (the wild-type strain of *M. robertsii*) and three independent isolates (*ΔPks2* #1, *ΔPks2* #2, and *ΔPks2* #3) of *M. robertsii*'s *Pks2* KO mutant *ΔPks2*. (**A**) Collapse rates of appressoria in the PEG8000 solution [80% (w/v)]. The assays were repeated three times with three replicates per repeat. Data are expressed as the mean ± SE. Values with different letters are significantly different ($P < 0.05$, Tukey's test in One-way ANOVA). (**B**) Appressoria (against a hydrophobic plastic surface) stained with Calcofluor Brightener White 2B. AP: appressorium; CO: conidium. Left panel: Bright field microscopy; Right panel: Fluorescence microscopy. Note: no difference in fluorescent intensity between WT and *ΔPks2*. Images are representative of at least three independent experiments for each condition. Scale bars represent 10μm.
