# Supplementary material for: Duplication of a Pks gene cluster and subsequent functional diversification facilitate environmental adaptation in Metarhizium species
Source: PLoS Genet. 2018 Jun 29;14(6):e1007472. doi: 10.1371/journal.pgen.1007472 (PMC6042797; doi:10.1371/journal.pgen.1007472)
Supplement: S14 Fig — M: DNA ladder (Genray, Shanghai); 1: The wild-type M. album; 2, 3, 4: three independent M. album transformants expressing M. robertsii’s Pks2 and Arp1. (PDF) [file pgen.1007472.s014.pdf]

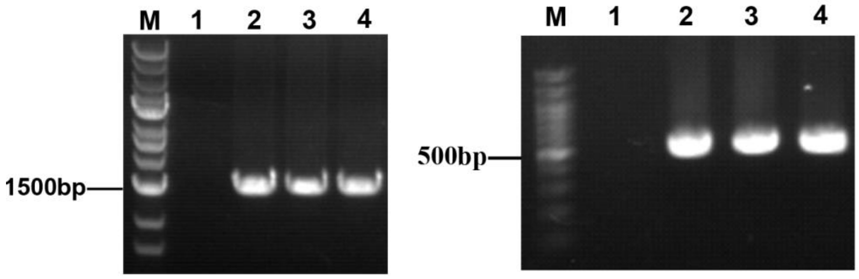

S14 Fig: RT-PCR confirmation of expression of *M. robertsii*'s *Pks2* (Left panel) and *Arp1* (Right panel) in *M. album*. M: DNA ladder (Genray, Shanghai); 1: the wild-type *M. album*; 2, 3, 4: three independent *M. album* transformants expressing *M. robertsii*'s *Pks2* and *Arp1*.
