# Supplementary material for: Duplication of a Pks gene cluster and subsequent functional diversification facilitate environmental adaptation in Metarhizium species
Source: PLoS Genet. 2018 Jun 29;14(6):e1007472. doi: 10.1371/journal.pgen.1007472 (PMC6042797; doi:10.1371/journal.pgen.1007472)
Supplement: S15 Fig — PCR reactions were conducted with primers MAA_Pks1_RT_F/R for Pks1 and MAA_Pks2_RT_F/R for Pks2 (see S9 Table for information about the primers). T1 to T5 represents five independent transformants expressing Pks1 or Pks2; CK: the positive control (the DNA template for PCR was M. robertsii’s genomic DNA). M: DNA Ladder (Tiangen Biotech, China). (PDF) [file pgen.1007472.s015.pdf]

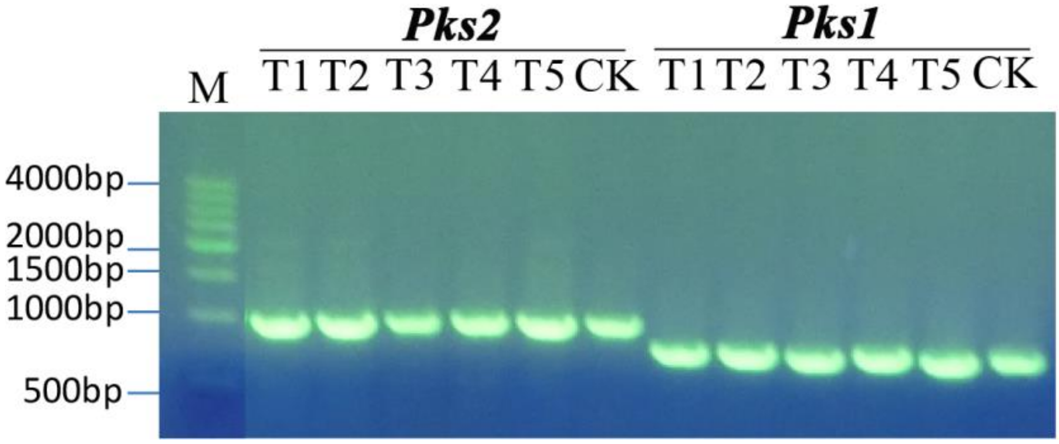

S15 Fig: Confirmation of the insertions of *M. robertsii*'s *Pks1* and *Pks2* into the genome of *A. nidulans* strain LO8030. PCR reactions were conducted with primers MAA_Pks1_RT_F/R for *Pks1* and MAA_Pks2_RT_F/R for *Pks2* (see S8 Table for information about the primers). T1 to T5 represents five independent transformants expressing *Pks1* or *Pks2*; CK: the positive control (the DNA template for PCR was *M. robertsii*'s genomic DNA). M: DNA Ladder (Tiangen Biotech, China).
