# Supplementary material for: Duplication of a Pks gene cluster and subsequent functional diversification facilitate environmental adaptation in Metarhizium species
Source: PLoS Genet. 2018 Jun 29;14(6):e1007472. doi: 10.1371/journal.pgen.1007472 (PMC6042797; doi:10.1371/journal.pgen.1007472)
Supplement: S16 Fig — This figure is supplemental to Fig 8C. Molecular weight of Compound I was detected by LC-MS analysis at m/z 315 [M+H]+, and 651 [M+Na]+. (PDF) [file pgen.1007472.s016.pdf]

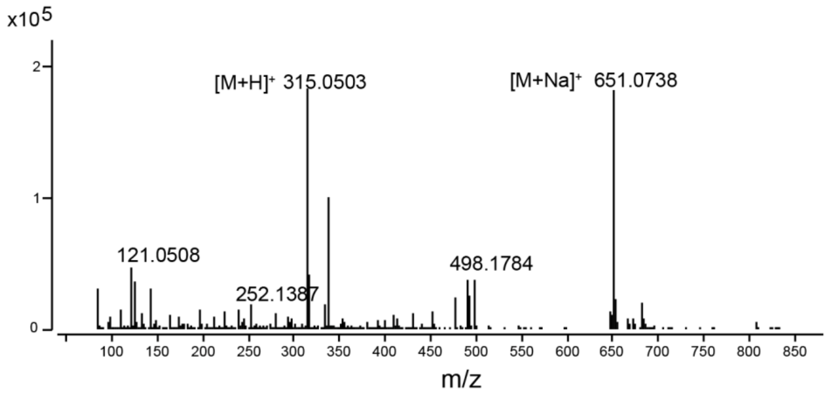

S16 Fig: LC-MS analysis of Compound I (shown in Fig 8) from the *A. nidulans* transformant expressing *Pks1*. This figure is supplemental to Fig. 8C. Molecular weight of Compound I was detected by LC-MS analysis at m/z 315 [M+H]+, and 651 [M+Na]+
