# Supplementary material for: Duplication of a Pks gene cluster and subsequent functional diversification facilitate environmental adaptation in Metarhizium species
Source: PLoS Genet. 2018 Jun 29;14(6):e1007472. doi: 10.1371/journal.pgen.1007472 (PMC6042797; doi:10.1371/journal.pgen.1007472)
Supplement: S19 Fig — (A) qRT-PCR confirmation of overexpression of Pks2 in ΔPks1. ΔPks1-Pks2OE: ΔPks1 with Pks2 overexpressed. 1, 2, 3: three independent isolates of ΔPks1-Pks2OE. The expression level in ΔPks1 is set to 1. Values with different letters are significantly different (P < 0.05, Tukey’s test in One-way ANOVA). All assays were repeated three times with three replicates per repeat. (B) Colony morphology of ΔPks1 and the three independent isolates (ΔPks1-Pks2OE-1, -2 and -3) of ΔPks1-Pks2OE. Note: all strains produce red conidia. Pictures were taken 18 days after inoculation of 5μl of conidial suspension (4×10^7^ conidia/ml) on a PDA plate. Scale bars represent 10mm. (C) HPLC detection of Compound I ΔPks1 and the three independent isolates (ΔPks1-Pks2OE-1, -2 and -3) of ΔPks1-Pks2OE. Standard: the purified Compound I, indicated by arrow, from the A. nidulans transformant TYPZ26.1. (PDF) [file pgen.1007472.s019.pdf]

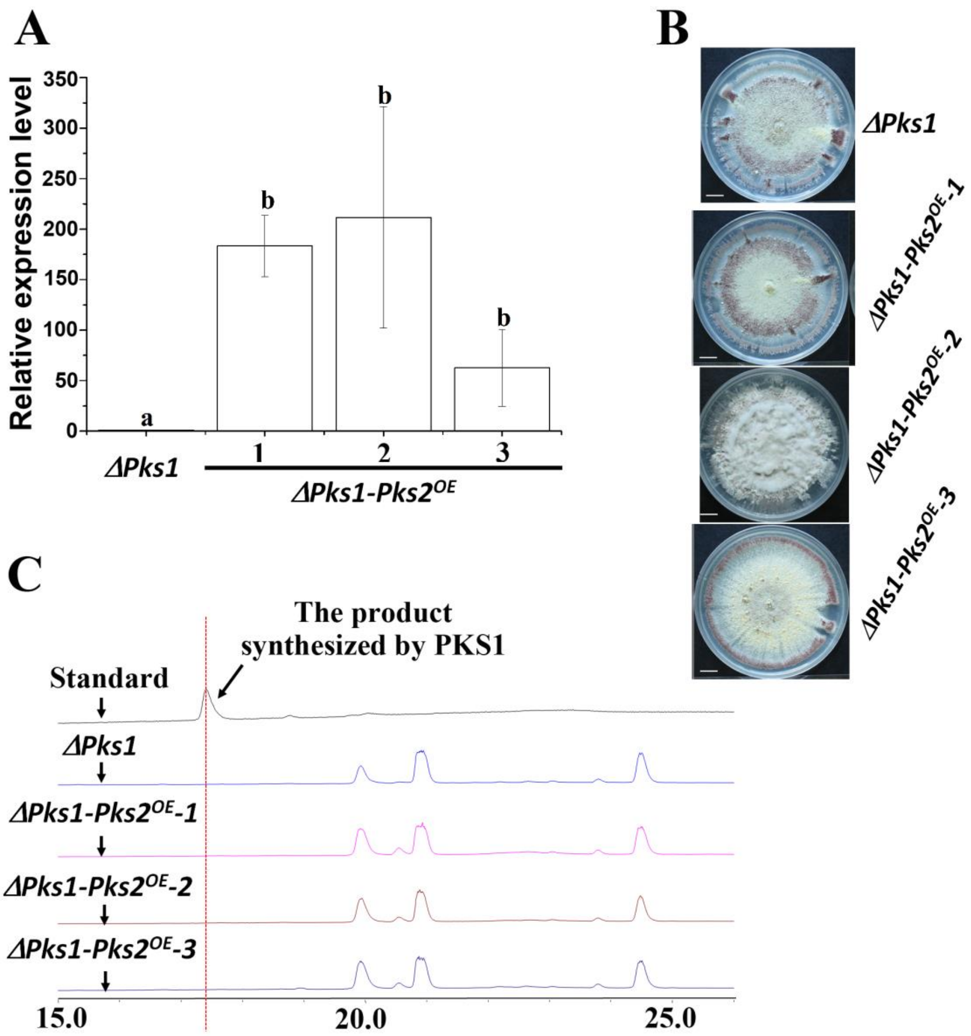

S19 Fig: *Pks2* cannot complement the *Pks1* KO mutant (*ΔPks1*) in *M. robertsii*. (**A**) qRT-PCR confirmation of overexpression of *Pks2* in *ΔPks1*. *ΔPks1-Pks2^{OE}*: *ΔPks1* with *Pks2* overexpressed. 1, 2, 3: three independent isolates of *ΔPks1-Pks2^{OE}*. The expression level in *ΔPks1* is set to 1. Values with different letters are significantly different ($P < 0.05$, Tukey's test in One-way ANOVA). All assays were repeated three times with three replicates per repeat. (**B**) Colony morphology of *ΔPks1* and the three independent isolates (*ΔPks1-Pks2^{OE}*-1, -2 and -3) of *ΔPks1-Pks2^{OE}*. Note: all strains produce red conidia. Pictures were taken 18 days after inoculation of 5μl of conidial suspension (4×10^7 conidia/ml) on a PDA plate. Scale bars represent 10mm. (**C**) HPLC detection of Compound I *ΔPks1* and the three independent isolates (*ΔPks1-Pks2^{OE}*-1, -2 and -3) of *ΔPks1-Pks2^{OE}*. Standard: the purified Compound I, indicated by arrow, from the *A. nidulans* transformant TYPZ26.1.
