# Supplementary material for: Duplication of a Pks gene cluster and subsequent functional diversification facilitate environmental adaptation in Metarhizium species
Source: PLoS Genet. 2018 Jun 29;14(6):e1007472. doi: 10.1371/journal.pgen.1007472 (PMC6042797; doi:10.1371/journal.pgen.1007472)
Supplement: S1 Table — (PDF) [file pgen.1007472.s020.pdf]

S1 Table: Information about proteins and their domain structures used in the analyses conducted in Fig 1.

Species	Protein	Accession #	Domain structure
Metarhizium robertsii	PKS1	XP_007823934.2	PF16073,PF00109,PF02801,PF00698,PF14765,PF00550,PF00975
Metarhizium robertsii	PKS2	XP_007819428.2	PF16073,PF00109,PF02801,PF00698,PF14765,PF00550,PF00975
Metarhizium anisopliae	PKS1	KID59669.1	PF16073,PF00109,PF02801,PF00698,PF14765,PF00550,PF00975
Metarhizium anisopliae	PKS2	KID71243.1	PF16073,PF00109,PF02801,PF00698,PF14765,PF00550,PF00975
Metarhizium brunneum	PKS1	XP_014539689.1	PF16073,PF00109,PF02801,PF00698,PF14765,PF00550,PF00975
Metarhizium brunneum	PKS2	XP_014545211.1	PF16073,PF00109,PF02801,PF00698,PF14765,PF00550,PF00975
Metarhizium guizhouense	PKS1	KID83385.1	PF16073,PF00109,PF02801,PF00698,PF14765,PF00550,PF00975
Metarhizium guizhouense	PKS2	KID86244.1	PF16073,PF00109,PF02801,PF00698,PF14765,PF00550,PF00975
Metarhizium majus	PKS1	XP_014573564.1	PF16073,PF00109,PF02801,PF00698,PF14765,PF00550,PF00975
Metarhizium majus	PKS2	XP_014575460.1	PF16073,PF00109,PF02801,PF00698,PF14765,PF00550,PF00975
Metarhizium acridum	PKS1	MG385100	PF16073,PF00109,PF02801,PF00698,PF14765,PF00550,PF00975
Metarhizium acridum	PKS2	XP_007815650.1	PF00109,PF02801,PF00698,PF14765,PF00550,PF00975
Metarhizium album	PKS1	MG385101	PF16073,PF00109,PF02801,PF00698,PF14765,PF00550,PF00975
Trichoderma reesei	Homolog of PKS1 and PKS2	ETR97354.1	PF16073,PF00109,PF02801,PF00698,PF14765,PF00550,PF00975
Fusarium graminearum	Homolog of PKS1 and PKS2	EYB30989.1	PF16073,PF00109,PF02801, PF16197,PF00698,PF14765,PF00550,PF00975
Colletotrichum fioriniae	Homolog of PKS1 and PKS2	XP_007599710.1	PF16073,PF00109,PF02801,PF00698,PF14765,PF00550,PF00975
Talaromyces marneffei	Homolog of PKS1 and PKS2	EEA24206.1	PF16073,PF00109,PF02801,PF00698,PF14765,PF00550,PF00975
Penicillium oxalicum	Homolog of PKS1 and PKS2	EPS34527.1	PF16073,PF00109,PF02801,PF00698,PF14765,PF00550,PF00975
Aspergillus fumigatus	Homolog of PKS1 and PKS2	XP_756095.1	PF16073,PF00109,PF02801,PF00698,PF14765,PF00550,PF00975
Aspergillus clavatus	Homolog of PKS1 and PKS2	XP_001276035.1	PF16073,PF00109,PF02801,PF00698,PF14765,PF00550,PF00975
Cladophialophora carrionii	Homolog of PKS1 and PKS2	XP_008730774.1	PF16073,PF00109,PF02801,PF00698,PF14765,PF00550,PF00975
Bipolaris oryzae	Homolog of PKS1 and PKS2	XP_007683730.1	PF16073,PF00109,PF02801, PF16197,PF00698,PF14765,PF00550,PF00975
Pyrenophora teres f.teres	Homolog of PKS1 and PKS2	XP_003298917.1	PF16073,PF00109,PF02801, PF16197,PF00698,PF14765,PF00550,PF00975
Sclerotinia sclerotiorum	Homolog of PKS1 and PKS2	XP_001585805.1	PF16073,PF00109,PF02801,PF00698,PF14765,PF00550,PF00975
Chaetomium globosum	Homolog of PKS1 and PKS2	XP_001219763.1	PF16073,PF00109,PF02801,PF00698,PF14765,PF00550,PF00975
Neurospora crassa	Homolog of PKS1 and PKS2	XP_960586.3	PF16073,PF00109,PF02801,PF00698,PF14765,PF00550,PF00975
Sordaria macrospora	Homolog of PKS1 and PKS2	CCC09041.1	PF16073,PF00109,PF02801,PF00698,PF14765,PF00550,PF00975
Podospora anserine	Homolog of PKS1 and PKS2	XP_001910795.1	PF16073,PF00109,PF02801,PF00698,PF14765,PF00550,PF00975
Magnaporthe oryzae	Homolog of PKS1 and PKS2	XP_003715434.1	PF16073,PF00109,PF02801,PF00698,PF14765,PF00550,PF00975
Grosmannia clavigera	Homolog of PKS1 and PKS2	XP_014172099.1	PF16073,PF00109,PF02801,PF00698,PF14765,PF00550,PF00975
Eutypa lata	Homolog of PKS1 and PKS2	XP-007796413.1	PF00109,PF02801,PF00698,PF14765,PF00550,PF00975
Scedosporium apiospermum	Homolog of PKS1 and PKS2	KEZ40717.1	PF16073,PF00109,PF02801,PF00698,PF14765,PF00550,PF00975
Verticillium dahliae	Homolog of PKS1 and PKS2	EGY13508.1	PF16073,PF00109,PF02801,PF00698,PF14765,PF00550,PF00975
Verticillium alfalfae	Homolog of PKS1 and PKS2	EEY14472.1	PF16073,PF00109,PF02801,PF00698,PF14765,PF00550,PF00975
Pestalotiopsis fici	Homolog of PKS1 and PKS2	ETS82099.1	PF16073,PF00109,PF02801,PF00698,PF14765,PF00550,PF00975
Exophiala dermatitidis	Homolog of PKS1 and PKS2	AAD31436.3	PF16073,PF00109,PF02801,PF00698,PF14765,PF00550,PF00975
Colletotrichum lagenaria	Homolog of PKS1 and PKS2	BAA18956.1	PF16073,PF00109,PF02801,PF00698,PF14765,PF00550,PF00975

Notes: PF16073 (SAT: starter unit acyltransferase), PF00109 (KS-N: N-terminus of β-ketoacyl synthase), PF02801 (KS-C: N-terminus of β-ketoacyl synthase), PF16197 (KA-C: ketoacyl-synthetase C-terminal extension), PF00698 (AT: acyltransferase), PF14765 (PS-DH: polyketide synthase dehydratase), PF00550 (PP-binding: Phosphopantetheine attachment site), PF00975 (TE: thioesterase).
