# Supplementary material for: Duplication of a Pks gene cluster and subsequent functional diversification facilitate environmental adaptation in Metarhizium species
Source: PLoS Genet. 2018 Jun 29;14(6):e1007472. doi: 10.1371/journal.pgen.1007472 (PMC6042797; doi:10.1371/journal.pgen.1007472)
Supplement: S2 Table — (PDF) [file pgen.1007472.s021.pdf]

S2 Table: Statistics of comparison of topologies of the constrained trees (S3 Fig) with the obtained tree (Fig 1)

Scenarios	au	np	bp	pp	kh	sh	wkh	wsh
**((((a,b),c),d),e)***	**1.000**	**1.000**	**1.000**	**1.000**	**0.999**	**1.000**	**0.999**	**1.000**
(((a,b),(c,d)),e)	**1e-08**	5e-07	0	2e-48	9e-05	9e-05	9e-05	4e-04
((((a,c),b),d),e)	**0.001**	3e-04	2e-04	3e-35	0.001	0.001	0.001	0.002
((((a,d),b),c),e)	**4e-04**	1e-04	1e-04	2e-38	0.001	0.001	0.001	0.001

Note: The tree was constructed with the amino acid sequences of KS domains in PKSs. Clades (a to e) are shown in S3 Fig. The value of site-wise likelihood of every constrained topology was calculated by PhyML. Hypothesis testing was implemented with CONSEL. All tests consistently showed the alternative (constrained) trees are statistically ($P < 0.05$) rejected. *: the obtained tree.
