# Supplementary material for: Duplication of a Pks gene cluster and subsequent functional diversification facilitate environmental adaptation in Metarhizium species
Source: PLoS Genet. 2018 Jun 29;14(6):e1007472. doi: 10.1371/journal.pgen.1007472 (PMC6042797; doi:10.1371/journal.pgen.1007472)
Supplement: S3 Table — Within each row (species), values appended by different letters are significantly different (P < 0.05, Tukey’s test in One-way ANOVA). All assays were repeated three times with three replicates per repeat. (PDF) [file pgen.1007472.s022.pdf]

S3 Table: GT_{50} (Time taken for 50% of conidia to germinate) values of three independent isolates of a *Pks1* mutant in seven *Metarhizium* species and their respective wild-type strains (WT) under optimal conditions (grown at 26°C in 1/2 SDY). Within each row (species), values appended by different letters are significantly different ($P < 0.05$, Tukey's test in One-way ANOVA). All assays were repeated three times with three replicates per repeat.

	GT_{50} (hours)			
Species	*WT*	Δ*Pks1-#1*	Δ*Pks1-#2*	Δ*Pks1-#3*
M. robertsii	6.74±0.12[a]	5.00±0.22[b]	5.23±0.14[b]	5.58±0.10[b]
M. anisopliae	7.95±0.12[a]	8.21±0.14[a]	8.62±0.31[a]	8.03±0.51[a]
M. brunneum	7.36±0.18[a]	7.56±0.22[a]	8.20±0.10[a]	8.26±0.06[a]
M. guizhouense	8.98±0.32[a]	9.53±0.27[a]	9.32±0.23[a]	9.30±0.24[a]
M. majus	12.61±0.33[a]	8.14±0.43[b]	7.71±1.0[b]	8.51±0.65[b]
M. acridum	13.7±0.13[a]	14.93±0.29[b]	14.81±0.18[b]	14.85±0.18[b]
M. album	9.08±0.16[a]	7.47±0.34[b]	7.71±0.17[b]	8.13±0.07[b]
