# Supplementary material for: Duplication of a Pks gene cluster and subsequent functional diversification facilitate environmental adaptation in Metarhizium species
Source: PLoS Genet. 2018 Jun 29;14(6):e1007472. doi: 10.1371/journal.pgen.1007472 (PMC6042797; doi:10.1371/journal.pgen.1007472)
Supplement: S4 Table — All assays were repeated three times with three replicates per repeat. (PDF) [file pgen.1007472.s023.pdf]

S4 Table: Relative germination rates of two of three independent isolates (#2 and #3) of a *Pks1* mutant in seven *Metarhizium* species under three abiotic stresses. All assays were repeated three times with three replicates per repeat.

	UV radiation		Heat stress		Cold stress	
	ΔPks1-#2	*ΔPks1-#3*	*ΔPks1-#2*	*ΔPks1-#3*	*ΔPks1-#2*	*ΔPks1-#3*
M. robertsii	Ref17[1]	Ref17[1]	0.66±0.004	0.61±0.007	2.60±0.07	2.60±0.06
M. anisopliae	0.35±0.05	0.40±0.02	0.52±0.11	0.64±0.13	3.17±0.02	3.21±0.007
M. brunneum	0.57±0.02	0.44±0.02	1.40±0.05	1.23±0.19	1.77±0.37	1.78±0.18
M. guizhouense	0.43±0.02	0.48±0.01	1.72±0.16	1.78±0.17	2.96±0.17	2.98±0.18
M. majus	0.13±0.02	0.14±0.14	2.27±1.39	2.83±1.26	1.75±0.07	1.72±0.08
M. acridum	0.54±0.04	0.59±0.003	0.13±0.01	0.11±0.02	2.41±0.07	2.46±0.07
M. album	0.31±0.03	0.27±0.004	4.59±0.2	4.37±0.11	3.09±0.21	2.79±0.02

Note:

1: data have been published in reference 17 in the main text.

2: The numerical values: the relative germination inhibition of a given stressor on each strain was calculated as (Gc-Gt)/ Gc, where Gc and Gt denote the GT_{50} (Time taken for 50% of conidia to germinate) of the stressed and unstressed conidia, respectively.

3: The data about the WT and *ΔPks1-#1* are presented in Table 1.
