# Supplementary material for: Duplication of a Pks gene cluster and subsequent functional diversification facilitate environmental adaptation in Metarhizium species
Source: PLoS Genet. 2018 Jun 29;14(6):e1007472. doi: 10.1371/journal.pgen.1007472 (PMC6042797; doi:10.1371/journal.pgen.1007472)
Supplement: S5 Table — All assays were repeated three times with three replicates per repeat. Within each row (strains), values appended by different letters are significantly different (P < 0.05, Tukey’s test in One-way ANOVA). (PDF) [file pgen.1007472.s024.pdf]

S5 Table: Relative germination rates of three independent isolates of the *M. robertsii Pks2* KO mutant under three abiotic stresses. All assays were repeated three times with three replicates per repeat. Within each row (strains), values appended by different letters are significantly different ($P < 0.05$, Tukey's test in One-way ANOVA).

	Strains			
	WT	*ΔPks2-#1*	*ΔPks2-#2*	*ΔPks2-#3*
UV radiation	0.26±0.05[a]	0.25±0.06[a]	0.28±0.03[a]	0.32±0.03[a]
Heat stress	0.47±0.03[a]	0.47±0.12[a]	0.45±0.08[a]	0.48±0.10[a]
Cold stress	2.16±0.15[a]	2.62±0.14[a]	2.89±0.34[a]	2.47±0.27[a]

Note:

The numerical values: the relative germination inhibition of a given stressor on each strain was calculated as (Gc-Gt)/ Gc, where Gc and Gt denote the GT_{50} (Time taken for 50% of conidia to germinate) of the stressed and unstressed conidia, respectively.
