# Supplementary material for: Duplication of a Pks gene cluster and subsequent functional diversification facilitate environmental adaptation in Metarhizium species
Source: PLoS Genet. 2018 Jun 29;14(6):e1007472. doi: 10.1371/journal.pgen.1007472 (PMC6042797; doi:10.1371/journal.pgen.1007472)
Supplement: S6 Table — (PDF) [file pgen.1007472.s025.pdf]

S6 Table: NMR spectroscopic data for Compound I (1-acetyl-2,4,6,8-tetrahydroxy-9,10-anthraquinone) in DMSO-d_6

Pos	Compound I	
	δ_H (J in Hz)	δ_C, mult.
1		134.6
2		165.8
3	5.71, s	108.1
4		165.8
4a		103.5
5	6.90, s	108.7
6		163.5
7	6.45, s	108.2
8		163.5
8a		134.6
9		184.7
9a		129.3
10		183.2
10a		109.4
1-Ac		205.5
	2.24, s	31.1
4-OH	13.0, br s	
8-OH	12.6, br s	
