# Supplementary material for: Duplication of a Pks gene cluster and subsequent functional diversification facilitate environmental adaptation in Metarhizium species
Source: PLoS Genet. 2018 Jun 29;14(6):e1007472. doi: 10.1371/journal.pgen.1007472 (PMC6042797; doi:10.1371/journal.pgen.1007472)
Supplement: S7 Table — (PDF) [file pgen.1007472.s026.pdf]

S7 Table: Plasmids and fungal strains used in this study.

Plasmids/Strains	Description	Reference
**Plasmids**		
Ppk2-OSCAR-GFP	Plasmid for construction of gene disruption plasmid	Xu et al., 2014
pA-Bar	Plasmid for construction of gene disruption plasmid	Xu et al., 2014
pPK2-Ptef	For expression of *Pks1* of *M. robertsii* in *M. album*	Guo et al., 2017
pYH-WA-pyrG	*URA3, WA flanking, AfpyrG, Amp*	Yin et al., 2013
pYH-WA-pyrG-gpdA	*URA3, WA flanking, Afribo, Amp, gpdA*	This study
pYPZ56.1	*gpdA(p)::Pks1* in pYH-WA-pyrG-gpdA	This study
***Metarhizium* strains**		
Metarhizium robertsii	ARSEF2575, a generalist	He et al., 2014
Metarhizium anisopliae	ARSEF549, a generalist	He et al., 2014
Metarhizium brunneum	ARSEF3297, a generalist	He et al., 2014
Metarhizium guizhouense	ARSEF977, a species with an intermediate host range	He et al., 2014
Metarhizium majus	ARSEF297, a species with an intermediate host range	He et al., 2014
Metarhizium acridum	ARSEF324, a specialist	He et al., 2014
Metarhizium album	ARSEF1941, a specialist	He et al., 2014
ΔMero-Hog1	The deletion mutant of *Hog1-MAPK* in *M. robertsii*	Chen et al., 2016
ΔMero-Fus3	The deletion mutant of *Fus3-MAPK* in *M. robertsii*	Chen et al., 2016
ΔMero-Slt2	The deletion mutant of *Slt2-MAPK* in *M. robertsii*	Chen et al., 2016
ΔMr-OPY2	The deletion mutant of *Mr-OPY2* in *M. robertsii*	Guo et al., 2017
***Aspergillus nidulans* background strains**		
LO8030	*pyroA4, riboB2, pyrG89, nkuA::argB, sterigmatocystin cluster (AN7804-AN7825)Δ, emericellamide cluster (AN2545-AN2549)Δ, asperfuranone cluster (AN1039-AN1029)Δ, monodictyphenone cluster (AN10023-AN10021) Δ, terrequinone cluster (AN8512-8520)Δ, austinol cluster part 1 (AN8379-AN8384)Δ, austinol cluster part 2 (AN9246-9259)Δ, F9775 cluster (AN7906-7915)Δ, asperthecin cluster (AN6000-AN6002)Δ*	(Chiang *et al*., 2016)
TYPZ26.1	*gpdA:: Pks1::AfpyrG* in LO8030	This study
