# Supplementary material for: Duplication of a Pks gene cluster and subsequent functional diversification facilitate environmental adaptation in Metarhizium species
Source: PLoS Genet. 2018 Jun 29;14(6):e1007472. doi: 10.1371/journal.pgen.1007472 (PMC6042797; doi:10.1371/journal.pgen.1007472)
Supplement: S8 Table — (PDF) [file pgen.1007472.s027.pdf]

S8 Table: Genbank accession numbers of the 20 genes in the 31 fungal species shown in Fig. 1A for constructing a species phylogenetic tree (shown in S4 Fig).

Species	*Cct8p*	*Elp3p*	*Erb1p*	*Gdh2p*	*Hsh49p*	*Icp55p*	*Ils1p*
Metarhizium robertsii	XP_007822063.1	XP_007821021.1	XP_007816448.1	XP_007818669.2	XP_007824065.1	XP_007818259.1	XP_007818881.1
Metarhizium anisopliae	KID67533.1	KID60893.1	KID59995.1	KID62296.1	KID65694.1	KID64927.1	KID69430.1
Metarhizium brunneum	XP_014549116.1	XP_014544685.1	XP_014543849.1	XP_014547521.1	XP_014542099.1	XP_014547075.1	XP_014545775.1
Metarhizium majus	KIE02969.1	KID97773.1	KID99460.1	KIE01579.1	KID99324.1	KIE03738.1	KIE02509.1
Metarhizium guizhouense	KID92455.1	KID88272.1	KID86369.1	KID92608.1	KID92893.1	KID93054.1	KID88763.1
Metarhizium acridum	XP_007810047.1	XP_007808318.1	XP_007815421.1	XP_007807988.1	XP_007812258.1	XP_007809077.1	XP_007815088.1
Metarhizium album	KHN99445.1	KHN97240.1	KHN98601.1	KHO01177.1	KHO01537.1	KHN99095.1	KHN99270.1
Colletotrichum fioriniae	EXF78020.1	EXF77681.1	EXF84209.1	EXF80548.1	EXF85243.1	EXF74986.1	EXF84702.1
Penicillium oxalicum	EPS27502.1	EPS34317.1	EPS31825.1	EPS31216.1	EPS26069.1	EPS26069.1	EPS25287.1
Cladophialophora carrionii	XP_008722627.1	XP_008721689.1	XP_008722236.1	XP_008731725.1	XP_008727797.1	XP_008727797.1	XP_008724927.1
Bipolaris oryzae	XP_007691475.1	XP_007684404.1	XP_007685934.1	XP_007685468.1	XP_007690870.1	XP_007683359.1	XP_007690027.1
Podospora anserina	XP_001908077.1	XP_001911227.1	XP_001908629.1	XP_007685468.1	XP_001911901.1	XP_001911901.1	XP_001909927.1
Eutypa lata	EMR61440.1	EMR63632.1	EMR68950.1	EMR69848.1	EMR71274.1	EMR66373.1	EMR64187.1
Pestalotiopsis fici	XP_007839431.1	XP_007837762.1	XP_007827689.1	XP_007832371.1	XP_007834982.1	XP_007836604.1	XP_007835797.1
Scedosporium apiospermum	XP_016642474.1	XP_016646421.1	XP_016644122.1	XP_016645106.1	XP_016639467.1	XP_016639467.1	XP_016643197.1
Colletotrichum orbiculare	ENH77904.1	ENH79595.1	ENH78230.1	ENH86491.1	ENH78714.1	ENH84068.1	ENH87515.1
Sclerotinia sclerotiorum	XP_001589935.1	XP_001592077.1	APA14678.1	APA08274.1	APA15410.1	XP_001596060.1	APA15569.1
Grosmannia clavigera	XP_014169118.1	XP_014170080.1	XP_014169301.1	XP_014172124.1	XP_014169262.1	XP_014171002.1	XP_014168829.1
Magnaporthe oryzae	XP_003718996.1	XP_003710346.1	XP_003709965.1	XP_003712804.1	XP_003719883.1	XP_003718443.1	XP_003716759.1
Sordaria macrospora	XP_003348967.1	XP_003348083.1	XP_003345367.1	XP_003348872.1	XP_003343751.1	XP_003349164.1	XP_003352371.1
Neurospora crassa	XP_958612.1	XP_961595.1	XP_964498.1	XP_956780.2	XP_011394630.1	XP_957892.2	XP_956014.1
Chaetomium globosum	XP_001229690.1	XP_001225084.1	XP_001220815.1	XP_001229575.1	XP_001224820.1	XP_001227014.1	XP_001225907.1
Verticillium dahliae	XP_009658230.1	XP_009652650.1	XP_009648857.1	XP_009653395.1	XP_009658474.1	XP_009652102.1	XP_009651278.1
Verticillium alfalfae	XP_003001140.1	XP_003000323.1	XP_003004962.1	XP_003003088.1	XP_003008488.1	XP_003001910.1	XP_003008697.1
Fusarium graminearum	XP_011326408.1	XP_011317913.1	XP_011318759.1	XP_011321519.1	XP_011327098.1	XP_011328452.1	XP_011325690.1
Trichoderma reesei	XP_006961598.1	XP_006967575.1	XP_006968690.1	XP_006963679.1	XP_006961444.1	XP_006967171.1	XP_006968110.1
Pyrenophora teres f. teres	EFQ85291.1	EFQ86708.1	EFQ87362.1	EFQ94916.1	EFQ87532.1	EFQ90078.1	EFQ90883.1
Exophiala dermatitidis	XP_009158357.1	XP_009152994.1	XP_009155097.1	XP_009160537.1	XP_009157164.1	XP_009158625.1	XP_009153963.1
Talaromyces marneffei	XP_002149209.1	XP_002148005.1	XP_002144223.1	XP_002144568.1	XP_002153252.1	XP_002144715.1	XP_002146145.1
Aspergillus clavatus	XP_001271853.1	XP_001273950.1	XP_001269336.1	XP_001275231.1	XP_001270028.1	XP_001268051.1	XP_001268885.1
Aspergillus fumigatus	XP_751852.1	XP_754018.1	XP_752287.1	XP_749729.1	XP_747559.1	XP_750807.2	XP_752741.1

S8 Table—continued.

Species	*Ilv2p*	*Kog1p*	*Mcm7p*	*Msh3p*	*Nhx1p*	*Pol1p*	*Pwp2p*
Metarhizium robertsii	XP_007816491.1	XP_007823548.1	XP_007821848.1	XP_007821854.1	XP_007819653.1	XP_007819778.1	XP_007823520.1
Metarhizium anisopliae	KID63066.1	KID67314.1	KID61946.1	KID68622.1	KID69615.1	KID69743.1	KID63814.1
Metarhizium brunneum	XP_014543805.1	XP_014541864.1	XP_014549333.1	XP_014549327.1	XP_014542447.1	XP_014542315.1	XP_014546680.1
Metarhizium majus	KID99502.1	KIE00282.1	KID99949.1	KID99955.1	KIE04015.1	KIE00358.1	KIE01762.1
Metarhizium guizhouense	KID86412.1	KID89497.1	KID91227.1	KID91221.1	KID89277.1	KID89166.1	KID87112.1
Metarhizium acridum	XP_007815194.1	XP_007807209.1	XP_007807415.1	XP_007807409.1	XP_007808877.1	XP_007814675.1	XP_007806813.1
Metarhizium album	KHN96382.1	KHN98809.1	KHN96339.1	KHN96333.1	KHO00707.1	KHO00834.1	KHN94619.1
Colletotrichum fioriniae	EXF74222.1	EXF81496.1	EXF75499.1	EXF75505.1	EXF79757.1	EXF82237.1	EXF76455.1
Penicillium oxalicum	EPS29798.1	EPS30447.1	EPS28829.1	EPS25882.1	EPS34358.1	EPS28898.1	EPS34493.1
Cladophialophora carrionii	XP_008724859.1	XP_008728362.1	XP_008724210.1	XP_008723156.1	XP_008727319.1	XP_008729389.1	XP_008726632.1
Bipolaris oryzae	XP_007686096.1	XP_007685643.1	XP_007688178.1	XP_007690385.1	XP_007684471.1	XP_007691119.1	XP_007686458.1
Podospora anserina	XP_001909102.1	XP_001906917.1	XP_001912892.1	XP_001908540.1	XP_001907928.1	XP_001908464.1	XP_001911998.1
Eutypa lata	EMR68719.1	EMR65652.1	EMR70165.1	EMR64149.1	EMR62303.1	EMR62541.1	EMR72470.1
Pestalotiopsis fici	XP_007827618.1	XP_007840655.1	XP_007827870.1	XP_007827789.1	XP_007838548.1	XP_007838538.1	XP_007834828.1
Scedosporium apiospermum	XP_016646615.1	XP_016639033.1	XP_016646218.1	XP_016646225.1	XP_016641374.1	XP_016646660.1	XP_016639443.1
Colletotrichum orbiculare	ENH79298.1	ENH86859.1	ENH88613.1	ENH88619.1	ENH80979.1	ENH81168.1	ENH88232.1
Sclerotinia sclerotiorum	XP_001592310.1	APA15738.1	APA06545.1	XP_001596685.1	APA10698.1	XP_001590014.1	APA13628.1
Grosmannia clavigera	XP_014171215.1	XP_014171821.1	XP_014169390.1	XP_014169283.1	XP_014169965.1	XP_014172589.1	XP_014173315.1
Magnaporthe oryzae	XP_003709592.1	XP_003720858.1	XP_003709949.1	XP_003709956.1	XP_003717132.1	XP_003717174.1	XP_003711369.1
Sordaria macrospora	XP_003348791.1	XP_003352471.1	XP_003347479.1	XP_003347472.1	XP_003348865.1	XP_003350431.1	XP_003352205.1
Neurospora crassa	XP_011394285.1	XP_965761.2	XP_963878.1	XP_963874.1	XP_956772.2	XP_961558.1	XP_961183.1
Chaetomium globosum	XP_001224230.1	XP_001222352.1	XP_001220297.1	XP_001220291.1	XP_001229540.1	XP_001221663.1	XP_001224902.1
Verticillium dahliae	XP_009650933.1	XP_009656124.1	XP_009648980.1	XP_009648971.1	XP_009653617.1	XP_009657651.1	XP_009648654.1
Verticillium alfalfae	XP_003009694.1	XP_003007560.1	XP_003005090.1	XP_003006012.1	XP_003003300.1	XP_003002438.1	XP_003006291.1
Fusarium graminearum	XP_011316846.1	XP_011320030.1	XP_011326807.1	XP_011326814.1	XP_011324058.1	XP_011323957.1	XP_011327144.1
Trichoderma reesei	XP_006961310.1	XP_006966540.1	XP_006963538.1	XP_006963537.1	XP_006967977.1	XP_006961367.1	XP_006969047.1
Pyrenophora teres f. teres	EFQ94642.1	EFQ92871.1	EFQ89239.1	EFQ87396.1	EFQ91068.1	EFQ93598.1	EFQ93355.1
Exophiala dermatitidis	XP_009154296.1	XP_009156855.1	XP_009152662.1	XP_009152652.1	XP_009157046.1	XP_009160480.1	XP_009154633.1
Talaromyces marneffei	XP_002143669.1	XP_002151673.1	XP_002146351.1	XP_002148394.1	XP_002147997.1	XP_002151991.1	XP_002147701.1
Aspergillus clavatus	XP_001271036.1	XP_001272957.1	XP_001275315.1	XP_001273287.1	XP_001273957.1	XP_001275367.1	XP_001276221.1
Aspergillus fumigatus	XP_754588.1	XP_749386.1	XP_755347.1	XP_749062.1	XP_754011.1	XP_755406.1	XP_748034.1

S8 Table—continued.

Species	*Rvb1p*	*Rvb2p*	*Ssl2p*	*Tif5p*	*Uba1p*	*Vma4p*
Metarhizium robertsii	XP_007825051.1	XP_007823866.1	XP_007822137.1	XP_007819774.1	XP_011410983.1	XP_007823571.1
Metarhizium anisopliae	KID63292.1	KID63392.1	KID68371.1	KID69739.1	KID67938.1	KID70062.1
Metarhizium brunneum	XP_014545129.1	XP_014545129.1	XP_014549042.1	XP_014542319.1	XP_014548615.1	XP_014541840.1
Metarhizium majus	KID96742.1	KID98626.1	KIE02895.1	KID98828.1	KID97099.1	KIE00258.1
Metarhizium guizhouense	KID91603.1	KID91499.1	KID90937.1	KID89170.1	KID86483.1	KID89507.1
Metarhizium acridum	XP_007813570.1	XP_007815922.1	XP_007806957.1	XP_007814679.1	XP_007810318.1	XP_007807198.1
Metarhizium album	KHO01946.1	KHO02041.1	KHO02008.1	KHO00830.1	KHN98973.1	KHN98823.1
Colletotrichum fioriniae	EXF83265.1	EXF86629.1	EXF85087.1	EXF82254.1	EXF75988.1	EXF85042.1
Penicillium oxalicum	EPS31755.1	EPS29619.1	EPS34518.1	EPS29411.1	EPS32734.1	EPS28405.1
Cladophialophora carrionii	XP_008722767.1	XP_008726041.1	XP_008726565.1	XP_008730980.1	XP_008729956.1	XP_008727953.1
Bipolaris oryzae	XP_007683050.1	XP_007690289.1	XP_007688697.1	XP_007692264.1	XP_007691633.1	XP_007686907.1
Podospora anserina	XP_001909908.1	XP_001910063.1	XP_001908365.1	XP_001907948.1	CDP26879.1	XP_001907388.1
Eutypa lata	EMR65983.1	EMR66649.1	EMR71163.1	EMR70767.1	EMR61381.1	EMR66927.1
Pestalotiopsis fici	XP_007838216.1	XP_007841693.1	XP_007832126.1	XP_007838617.1	XP_007831159.1	XP_007837312.1
Scedosporium apiospermum	XP_016644566.1	XP_016643586.1	XP_016639254.1	XP_016645028.1	XP_016640825.1	XP_016639121.1
Colletotrichum orbiculare	ENH87722.1	ENH87848.1	ENH85168.1	ENH81153.1	ENH88049.1	ENH86989.1
Sclerotinia sclerotiorum	XP_001593913.1	XP_001597906.1	XP_001591079.1	XP_001585325.1	APA12814.1	XP_001594159.1
Grosmannia clavigera	XP_014173578.1	XP_014168183.1	XP_014168336.1	XP_014173332.1	XP_014173053.1	XP_014168598.1
Magnaporthe oryzae	XP_003719906.1	XP_003716817.1	XP_003717094.1	XP_003713892.1	XP_003714354.1	XP_003720979.1
Sordaria macrospora	XP_003346757.1	XP_003346757.1	XP_003350492.1	XP_003344256.1	XP_003348751.1	XP_003347016.1
Neurospora crassa	XP_955769.1	XP_963328.1	XP_957329.1	XP_957252.3	XP_956143.2	XP_011393174.1
Chaetomium globosum	XP_001225843.1	XP_001228173.1	XP_001221811.1	XP_001219780.1	XP_001224114.1	XP_001222569.1
Verticillium dahliae	XP_009654206.1	XP_009655011.1	XP_009652129.1	XP_009653581.1	XP_009650094.1	XP_009656091.1
Verticillium alfalfae	XP_003004243.1	XP_003004082.1	XP_003006693.1	XP_003003264.1	XP_003009129.1	XP_003007598.1
Fusarium graminearum	XP_011323653.1	XP_011324910.1	XP_011327021.1	XP_011323961.1	XP_011328016.1	XP_011320157.1
Trichoderma reesei	XP_006961081.1	XP_006960986.1	XP_006963440.1	XP_006961826.1	XP_006962650.1	XP_006966623.1
Pyrenophora teres f. teres	EFQ95589.1	EFQ96159.1	EFQ84865.1	EFQ86285.1	EFQ85131.1	EFQ90571.1
Exophiala dermatitidis	XP_009158557.1	XP_009155307.1	XP_009155640.1	XP_009161496.1	XP_009153236.1	XP_009157240.1
Talaromyces marneffei	XP_002151212.1	XP_002149076.1	XP_002147757.1	XP_002146590.1	XP_002145371.1	XP_002143486.1
Aspergillus clavatus	XP_001271944.1	XP_001269889.1	XP_001276200.1	XP_001267824.1	XP_001275860.1	XP_001270329.1
Aspergillus fumigatus	XP_751754.1	XP_749991.1	XP_748011.1	XP_755233.1	XP_755911.1	XP_747868.1
