# Supplementary material for: Duplication of a Pks gene cluster and subsequent functional diversification facilitate environmental adaptation in Metarhizium species
Source: PLoS Genet. 2018 Jun 29;14(6):e1007472. doi: 10.1371/journal.pgen.1007472 (PMC6042797; doi:10.1371/journal.pgen.1007472)
Supplement: S9 Table — (PDF) [file pgen.1007472.s028.pdf]

S9 Table: Primers used in this study.

Primer	Sequence	Usage
Bar-up	CGCCTGGACGACTAAACC	Confirmation of the gene disruptions
Bar-down	TCAGCCTGCCGGTACCGC	
ΔMAA-Pks2-5-1	GGGGACAGCTTTCTTGTACAAAGTGGAAGTTGTGACTTCGGGTAG	Disruption of *Pks2 in M. robertsii*
ΔMAA-Pks2-5-2	GGGGACTGCTTTTTTGTACAAACTTGTGTGGACAGTATGCTATC	
ΔMAA-Pks2-3-1	GGGGACAACTTTGTATAGAAAAGTTGTTAAGACGTCGACATGAAC	
ΔMAA-Pks2-3-2	GGGGACAACTTTGTATAATAAAGTTGTTCCGTGCTGTATCCATC	
ΔMAA-Pks2-CF-1	AATCACACGTTCCGCAC	Confirmation of *Pks2* disruption *in M. robertsii*
ΔMAA-Pks2-CF-2	GTGCTCTTGTCGTGAAC	
ΔMAN-Pks1-5-1	GGGGACAGCTTTCTTGTACAAAGTGGAAGAATGAGGACCGTCTTG	Disruption of *Pks1* in *M. anisopliae*
ΔMAN-Pks1-5-2	GGGGACTGCTTTTTTGTACAAACTTGTGCTTACCAAGTGGTCAC	
ΔMAN-Pks1-3-1	GGGGACAACTTTGTATAGAAAAGTTGTTAGGTTCTGCTCATGATG	
ΔMAN-Pks1-3-2	GGGGACAACTTTGTATAATAAAGTTGTGCACGTCCAAGAAGTTG	
ΔMAN-Pks1-CF-1	CGCACGAAAGTCTTCAC	Confirmation of the *Pks1*disruption in *M. anisopliae*
ΔMAN-Pks1-CF-2	ATGTTGGCCTTGACAAG	
ΔMBR-Pks1-5-1	GGGGACAGCTTTCTTGTACAAAGTGGAAATTTCCTCTGCCATGTG	Disruption of *Pks1* in *M. brunneum*
ΔMBR-Pks1-5-2	GGGGACTGCTTTTTTGTACAAACTTGTCTTGAGCACAGACATTG	
ΔMBR-Pks1-3-1	GGGGACAACTTTGTATAGAAAAGTTGTTACGAACGCAACGTTCAC	
ΔMBR-Pks1-3-2	GGGGACAACTTTGTATAATAAAGTTGTGTTGCGCATCAAAGATG	
ΔMBR-Pks1-CF-1	TGATCAAGGTTCTGCTC	Confirmation of *Pks1*disruption in *M. brunneum*
ΔMBR-Pks1-CF-2	CATGCTTCAAAGTCGTG	
ΔMGU-Pks1-5-1	GGGGACAGCTTTCTTGTACAAAGTGGAATTATCGTCGCGCAACAC	Disruption of *Pks1* in *M. guizhouense*
ΔMGU-Pks1-5-2	GGGGACTGCTTTTTTGTACAAACTTGTGTACATTGAGCCATGAG	
ΔMGU-Pks1-3-1	GGGGACAACTTTGTATAGAAAAGTTGTTAGGTTCTGCTCATGATG	
ΔMGU-Pks1-3-2	GGGGACAACTTTGTATAATAAAGTTGTCGAAGCCTTCGTCTTTG	
ΔMGU-Pks1-CF-1	TGCTTCGCTCCATCAAC	Confirmation of *Pks1*disruption in *M. guizhouense*
ΔMGU-Pks1-CF-2	CATCTTTGTTGCGCATC	
MAJ-Pks1-RNAi-5	gggaattcCGTGCTACAGCGGATGCCGGTCCAAAGTCCgatacgctggcctgccc	Cloning the genomic clone for knocking down *Pks1* in *M. majus*
MAJ-Pks1-RNAi-3	ggtctagaCGTGCTACAGCGGATGCCGGTCCAAAGTCCtgggaaagcgcgttaca	
MAJ-Pks1-Promoter-F	ccgaattcAGAGCACTCAAATCATAG	Cloning the promoter for knocking down *Pks1* in *M. majus*
MAJ-Pks1-Promoter-R	ccgaattcGTTGATCCGAAGGTTGC	
ΔMAC-Pks1-5-1	GGGGACAGCTTTCTTGTACAAAGTGGAAGGGCATACTCTAATTTG	Disruption of *Pks1* in *M. acridum*
ΔMAC-Pks1-5-2	GGGGACTGCTTTTTTGTACAAACTTGTTCTGTCTTCTGGTTATG	
ΔMAC-Pks1-3-1	GGGGACAACTTTGTATAGAAAAGTTGTTATCATTAGACCTGCGAG	
ΔMAC-Pks1-3-2	GGGGACAACTTTGTATAATAAAGTTGTATGTAGCTGACTTGGTG	
ΔMAC-Pks1-CF-1	ATTTCCAGCTCCTCTAG	Confirmation of *Pks1* disruption in *M. acridum*
ΔMAC-Pks1-CF-2	GTGAAGACTTTCGTGAG	
MAM-Pks1-RNAi-5	gggaattcGCTTTGTTCAGTCACCAAGCCTCGGCCAATgatacgctggcctgccc	Cloning the promoter for knocking down *Pks1* in *M. album*
MAM-Pks1-RNAi-3	ggtctagaGCTTTGTTCAGTCACCAAGCCTCGGCCAATtgggaaagcgcgttaca	
MAM-Pks1-Promoter-F	ccgaattcGTAAGCAATCTGTCTTATC	Cloning the promoter for knocking down *Pks1* in *M. album*
MAM-Pks1-Promoter-R	ccgaattcGTTGATGCAGGCAGGCGTC	
MAA-Gpd-RT-5	GTCGTCATCTCTGCTCCCTC	*gpd* gene in *M. robertsii*
MAA-Gpd-RT-3	CAATGGTGAACTTGTCGTGG	
MAA-Tef-RT-5	AGGCTGACTGCGCTATTCTC	*tef* gene in *M. robertsii*
MAA-Tef-RT-3	ACTTGGTGGTGTCCATCTTG	
MAA-Pks1-RT-5	AACTACTTTGGAGACGGCCA	qRT-PCR for *Pks1* in *M. robertsii*

MAA-Pks1-RT-3	ACCAAGAGGCCAACTTTGAG	
MAA-Pks2-RT-5	GCACTGTCGAGAACCTGTCA	qRT-PCR for *Pks2* in *M. robertsii*
MAA-Pks2-RT-3	CACCTCGTCCCAGTTGATTT	
MAN-Pks1-RT-5	ACGATCAAGCAGTCTGATAC	qRT-PCR for *Pks1* in *M. anisopliae*
MAN-Pks1-RT-3	ACTAGAAAAGGCAGGGAATG	
MAN-Pks2-RT-5	TGGCCTGTTTCATCTACAAA	qRT-PCR for *Pks2* in *M. anisopliae*
MAA-Pks2-RT-3	CCAGCCACTATTACATCTCC	
MBR-Tef-RT-5	TATTCTCATTATCGCTGCCG	*tef* in *M. brunneum*
MBR-Tef-RT-3	CTCCTTGATGATTTCCTGGT	
MBR-Pks1-RT-5	ACGATCAAGCAGTCTGATAC	qRT-PCR for *Pks1* in *M. brunneum*
MBR-Pks1-RT-3	TCGAGCAACCAGATTTTGTA	
MBR-Pks2-RT-5	AAAGTTTGCACAAGACATGG	qRT-PCR for *Pks2* in *M. brunneum*
MBR-Pks2-RT-3	TTGCTGTGATAGTTTCGGAA	
MGU-Gpd-RT-5	AAGAAGGTCATCATCTCTGC	*gpd* in *M. guizhouense*
MGU-Gpd-RT-3	CTCGACAATGGTGAACTTGT	
MGU-Pks1-RT-5	ACGATCAAGCAGTCTGATAC	qRT-PCR for *Pks1* in *M. guizhouense*
MGU-Pks1-RT-3	ACTAGAAAAGGCAGGGAATG	
MGU-Pks2-RT-5	AATTTGCACAAGACATGGAC	qRT-PCR for *Pks2* in *M. guizhouense*
MGU-Pks2-RT-3	TGGGAAGGTGTATTTTCTCG	
MAJ-Gpd-RT-5	CAGCTTTACTCTTGCGAATG	*gpd* in *M. majus*
MAJ-Gpd-RT-3	AGAACTTGACCTTCTTGCC	
MAJ-Pks1-RT-5	ACGATCAAGCAGTCTGATAC	qRT-PCR for *Pks1* in *M. majus*
MAJ-Pks1-RT-3	TCGAGCAACCAGATTTTGTA	
MAJ-Pks2-RT-5	TTTTGGCGAGAAAATACAGC	qRT-PCR for *Pks2* in *M. majus*
MAJ-Pks2-RT-3	GTGACAGTTTTGGAATTGGG	
MAC-Gpd-RT-5	CCAGCTTTTAGTTTTGCGAA	*gpd* in *M. acridum*
MAC-Gpd-RT-3	TTGATGTCGCCCTTGAAAAT	*tef* in *M. acridum*
MAC-Tef-RT-5	GGGTAAGGAAGACAAGACTC	
MAC-Tef-RT-3	TGTAGGCTCCAAAACATACC	
MAC-Pks1-RT-5	CAAGAGGCATATCCAAC	qRT-PCR for *Pks1* and the fragment PS2 in Fig. S1 in *M. acridum*
MAC-Pks1-RT-3	GTCCGAGCAACCAAGAG	
MAC-Pks2-RT-5	GAAATAATGGAAGCTGTCGC	qRT-PCR for *Pks2* in *M. acridum*
MAC-Pks2-RT-3	ACACCCACCCAATACTTTAC	
MAM-Gpd-RT-5	AAGAAGGTCATCATCTCTGC	*gpd* in *M. album*
MAM-Gpd-RT-3	CCTCAACAATGGTGAACTTG	
MAM-Pks1-RT-5	CCAAATACCCACGTCTC	qRT-PCR for *Pks1* and the fragment PS2 in Fig. S1 in *M. album*
MAM-Pks1-RT-3	AGAGGACGAGCAAGAAG	
MAC-Pks1-RT-6-F	CCAGCTGTACGATCCTG	RT-PCR analysis of the fragment PS3 in *M. acrdum* in Fig. S1
MAC-Pks1-RT-6-R	CACCTTCTCCAAGAATC	
MAM-Pks1-RT-6-F	CTGATTATGACTGCATC	RT-PCR analysis of the fragment PS3 in *M. album* in Fig. S1
MAM-Pks1-RT-6-R	GCAAACTTCTGGTGGTC	
PS1-5	MAC-Pks1-RT-6-F/ MAM-Pks1-RT-6-F	RT-PCR analysis of the fragment PS1 in Fig. S1.
PS1-3	MAC-Pks1-RT-3/MAM-Pks1-RT-3	
MAC-Pks1-Re-F	ATGGAACACGTGACGATC	RT-PCR analysis of the fragment PS4 in *M. acridum* in Fig. S1.
MAC-Pks1-Re-R	GCAACCAAGAGGCCAAC	
MAM-Pks1-Re-F	ATGGAGCACGTGATAATC	RT-PCR analysis of the fragment PS4 in *M. album* in Fig. S1.
MAM-Pks1-Re-R	TTGAGTGACACAAGGAC	
MAA_Arp1-5	GGATCCATGAACCAATCTTTAACCC	Cloning the genomic clone of M. robertsii *Arp1* for constructing expression plasmid
MAA_Arp1-3	GATATCCTACGAATGTTTCTCCGAC	

MAA-Pks2-OE-5	GGATTTAAATATGGCAACGCAGGATGCGCCTAT	Cloning the genomic clone of *M. robertsii Pks2* for constructing overexpressing vector
MAA-Pks2-OE-3	GGATTTAAATCTAGCTGGGACCAAGCAAAAATTC	
MAA-Pks2-SEQ-F	AGTCCAGCAACGGACAG	Confirming the insertion of *M. robertsii Pks2* into the genomes of *M. album* and *ΔPks2*
Trpc-seq-5	TTCTTGTTGACATGGAG	
MAA_Pks1_F1	CTTCAGTATATTCATCTTCCCATCCAAGAACCTTTAATCGATGAACCACGTGACGATCAAGCAG	Amplification of *M. robertsii*'s *Pks1* for heterologous expression in *A. nidulans*
MAA_Pks1_R1	CTCGACTTGCGAAGCTATGGC	
MAA_Pks1_F2	GATGCTGGCCATCCGAAGCAG	
MAA_Pks1_R2	CACAACATATTTCGTCAGACACAGAATAACTCTCGCTAGCGCGTTGAGAGACCATAAACAATG	
MAA_Pks1_RT_F	CTGGTCAGCGATTGGCTCTAG	Confirmation of the insertion of *Pks1* into the genome of *A. nidulans*
MAA_Pks1_RT_R	GTCGTGATCTGCAATGGCATC	
MAA_Pks2_F1	CTTCAGTATATTCATCTTCCCATCCAAGAACCTTTAATCGCCGTCAAGAGAAGAGCTGTACTG	Amplification of *M. robertsii*'s *Pks2* for heterologous expression in *A. nidulans*
MAA_Pks2_R1	GCACCACAATGGTAACCAAGG	
MAA_Pks2_F2	CTTGCTGCGCTGTATCAGGC	
MAA_Pks2_R2	CACAACATATTTCGTCAGACACAGAATAACTCTCGCTAGGACTGGCTGCTCGATTGGACC	
MAA_Pks2_RT_F	GCATATCGCCCAAGGAGGCAG	Confirmation of the insertion of *Pks2* into the genome of *A. nidulans*
MAA_Pks2_RT_R	CGAAGGTGCGCAGGACAGAAC	
The loop DNA	GATACGCTGGCCTGCCCAACCTTTCGGTATAAAGACTTCGCGCTGATACCAGACGTTGCCCGCATAATTACGAATATCTGCATCGGCGAACTGATCGTTAAAACTGCCTGGCACAGCAATTGCCCGGCTTTCTTGTAACGCGCTTTCCCA	The DNA fragment for the loop in a dsRNA for RNAi
