# Supplementary material for: Duplication of a Pks gene cluster and subsequent functional diversification facilitate environmental adaptation in Metarhizium species
Source: PLoS Genet. 2018 Jun 29;14(6):e1007472. doi: 10.1371/journal.pgen.1007472 (PMC6042797; doi:10.1371/journal.pgen.1007472)
Supplement: S3 Dataset — (PDF) [file pgen.1007472.s031.pdf]

Supplementary dataset 3: NOTUNG assays using DL or DTL model with different parameter combinations

Figures showing the results about estimation of gene duplication, loss and transfer events of the *Pks* genes in the fungal species shown in Fig 1A by reconciling the raw *Pks* gene's ML tree (Fig 1A) (**A**) or the rearranged ML tree of the *Pks* genes (Fig 2A) (**B**), with the species tree (Fig S4) using NOTUNG with a duplication-transfer-loss (DTL) model with default parameters (1.5 for duplication, 3.0 for transfer, 1.0 for loss), and five other parameter combinations (1.0 for duplication, 3.0 for transfer, 1.0 for loss; 2.0 for duplication, 3.0 for transfer, 1.0 for loss; 1.5 for duplication, 2.5 for transfer, 1.0 for loss; 1.5 for duplication, 3.0 for transfer, 0.5 for loss; 1.5 for duplication, 3.0 for transfer, 1.5 for loss). The figure showing the result about estimation with each parameter combination is presented below. In each figure, red circles indicate duplication events. Cyan clades indicate transfer events. Gray clades indicate loss events. Numbers in black at nodes represent the bootstrap values of Maximum Likelihood. Blue notes near the nodes denote the internal node species name.

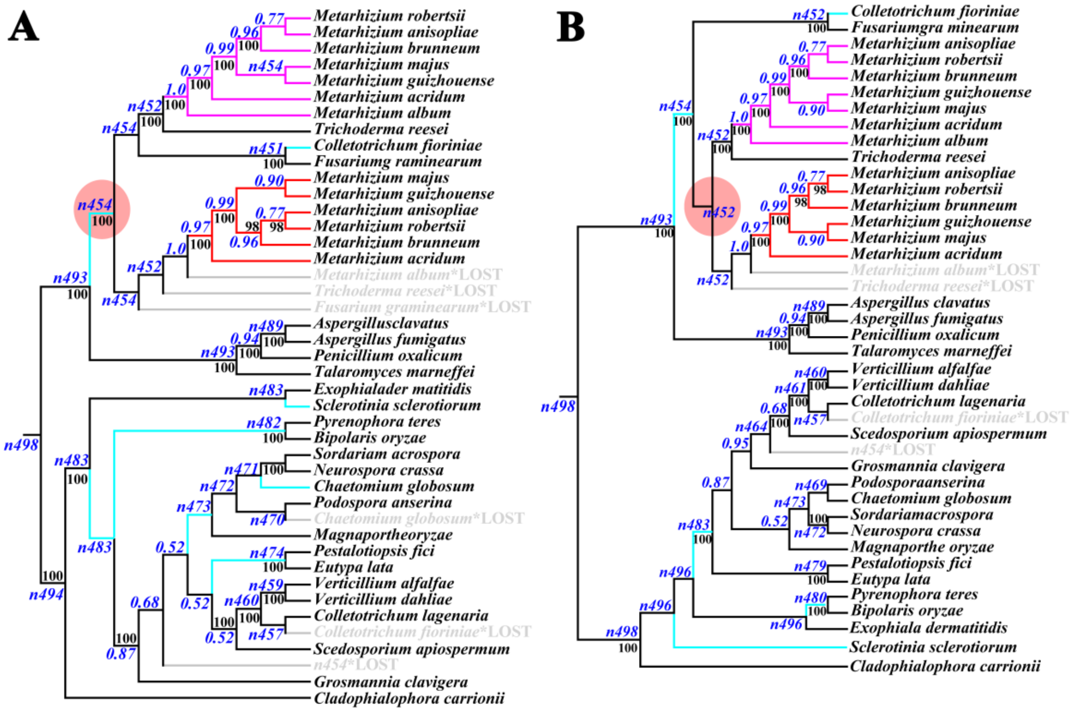

Figure SD6-1: Reconciliation of the raw *Pks* gene's ML tree (Fig 1A) (**A**) or the rearranged ML tree of the *Pks* genes (Fig 2A) (**B**), with the species tree (Fig S4) using NOTUNG with a duplication-transfer-loss (DTL) model with the default parameters (1.5 for duplication, 3.0 for transfer, 1.0 for loss)

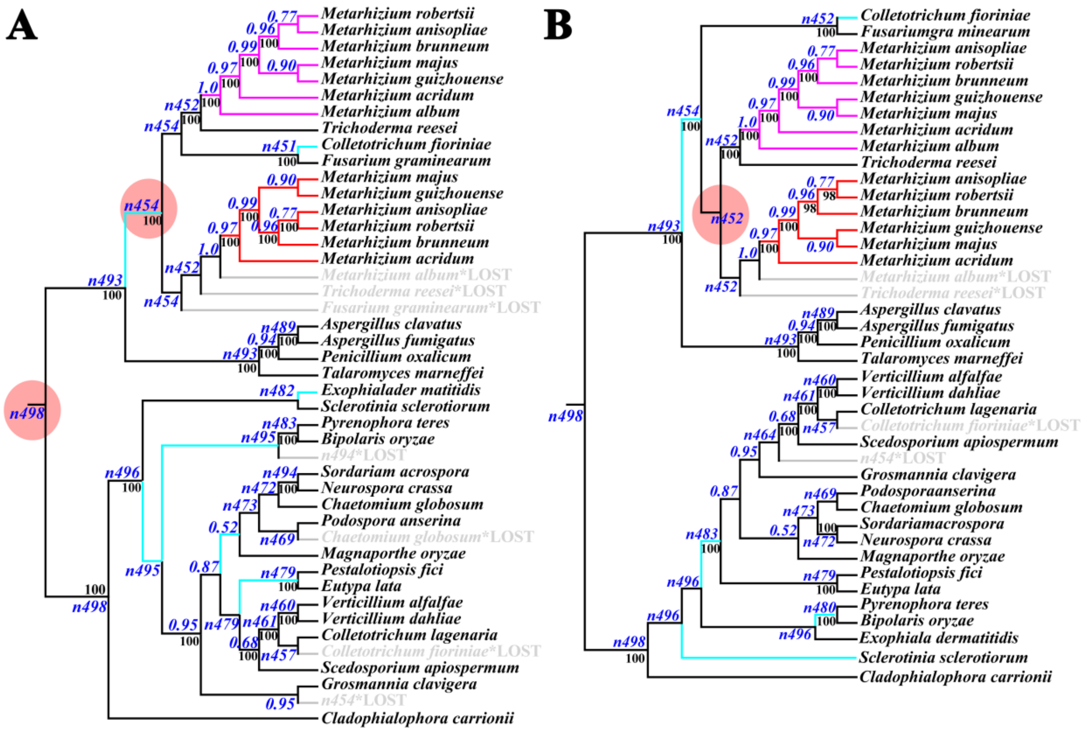

Figure SD6-2: Reconciliation of the raw *Pks* gene's ML tree (Fig 1A) (**A**) or the rearranged ML tree of the *Pks* genes (Fig 2A) (**B**), with the species tree (Fig S4) using NOTUNG with a duplication-transfer-loss (DTL) model with the defined parameters (1.0 for duplication, 3.0 for transfer, 1.0 for loss).

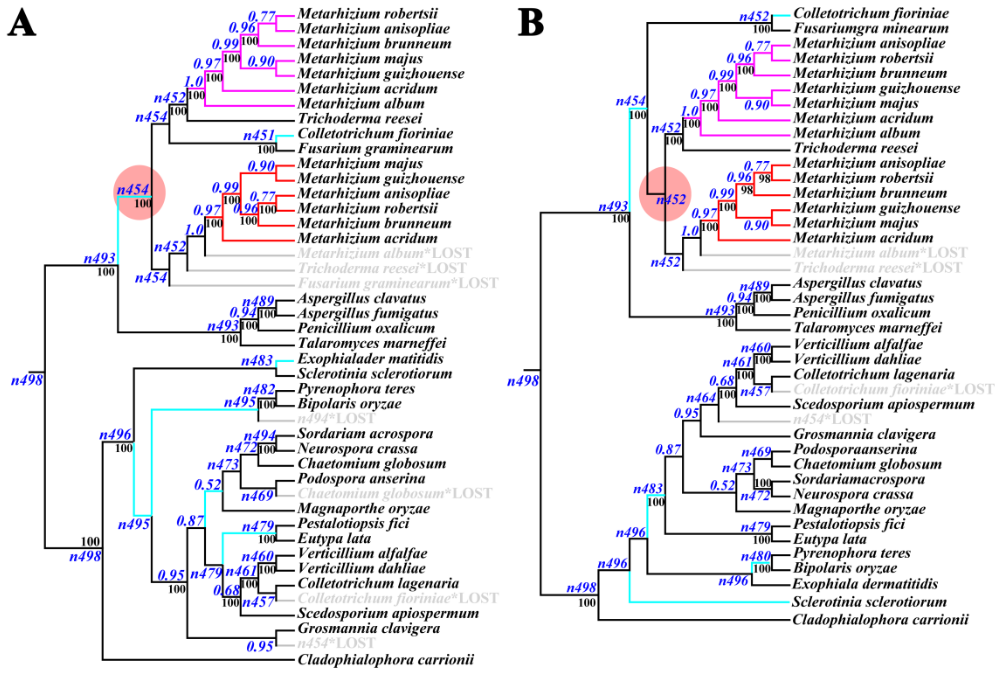

Figure SD6-3: Reconciliation of the raw *Pks* gene's ML tree (Fig 1A) (**A**) or the rearranged ML tree of the *Pks* genes (Fig 2A) (**B**), with the species tree (Fig S4) using NOTUNG with a duplication-transfer-loss (DTL) model with the defined parameters (1.5 for duplication, 2.5 for transfer, 1.0 for loss).

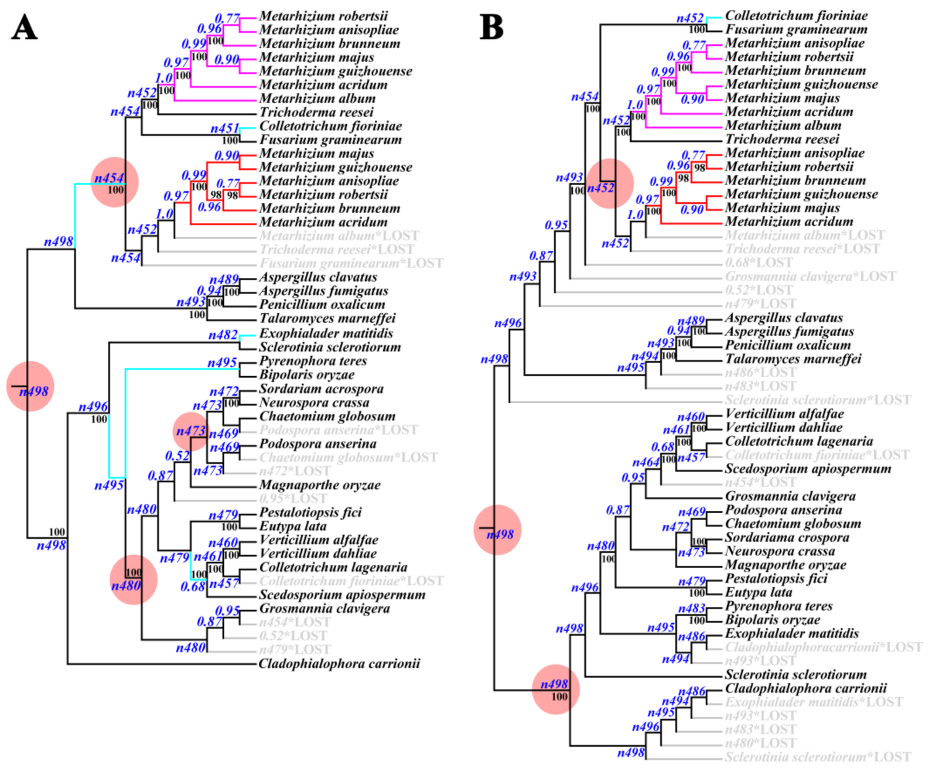

Figure SD6-4: Reconciliation of the raw *Pks* gene's ML tree (Fig 1A) (**A**) or the rearranged ML tree of the *Pks* genes (Fig 2A) (**B**), with the species tree (Fig S4) using NOTUNG with a duplication-transfer-loss (DTL) model with the defined parameters (1.5 for duplication, 3.0 for transfer, 0.5 for loss).

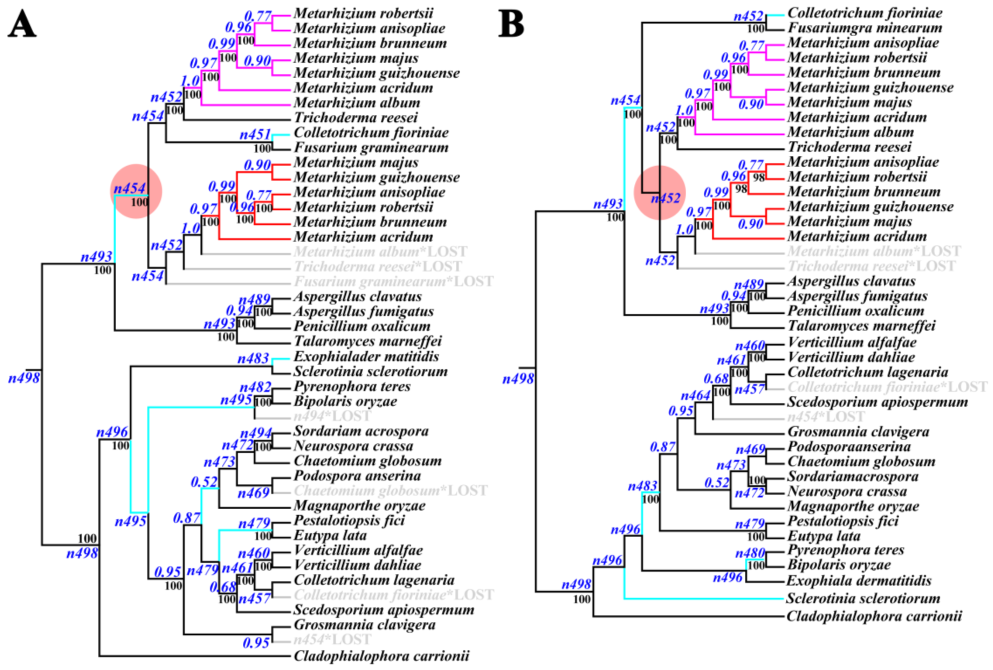

Figure SD6-5: Reconciliation of the raw *Pks* gene's ML tree (Fig 1A) (**A**) or the rearranged ML tree of the *Pks* genes (Fig 2A) (**B**), with the species tree (Fig S4) using NOTUNG with a duplication-transfer-loss (DTL) model with the defined parameters (1.5 for duplication, 3.0 for transfer, 1.5 for loss).

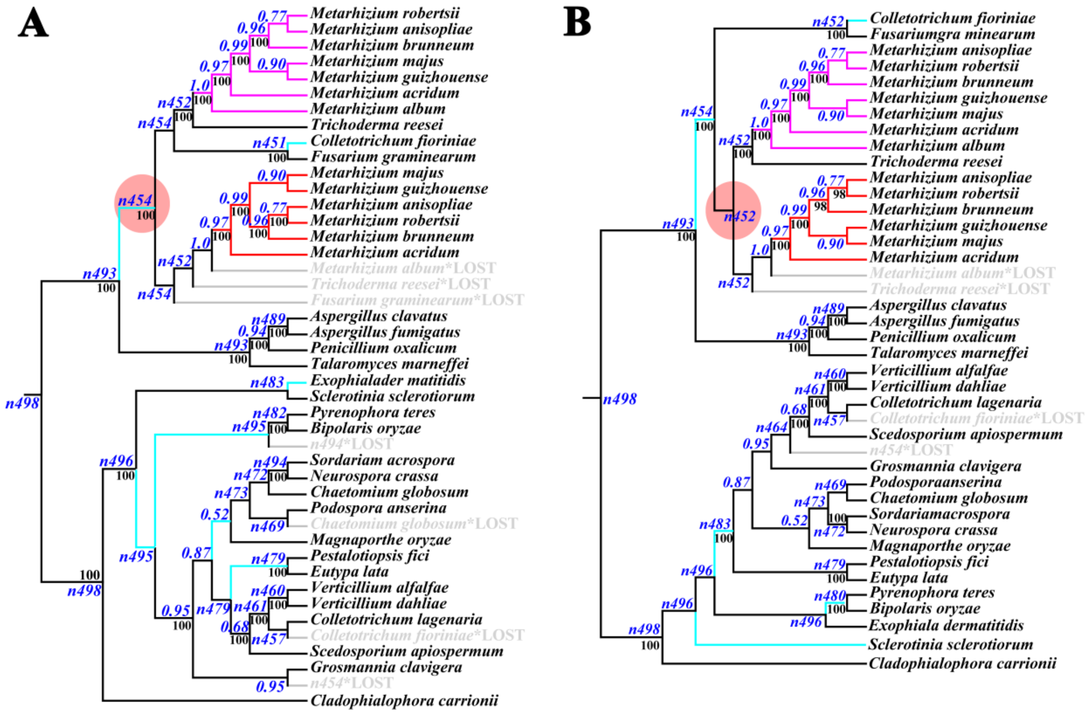

Figure SD6-6: Reconciliation of the raw *Pks* gene's ML tree (Fig 1A) (**A**) or the rearranged ML tree of the *Pks* genes (Fig 2A) (**B**), with the species tree (Fig S4) using NOTUNG with a duplication-transfer-loss (DTL) model with the defined parameters (2.0 for duplication, 3.0 for transfer, 1.0 for loss).
