# Supplementary material for: Duplication of a Pks gene cluster and subsequent functional diversification facilitate environmental adaptation in Metarhizium species
Source: PLoS Genet. 2018 Jun 29;14(6):e1007472. doi: 10.1371/journal.pgen.1007472 (PMC6042797; doi:10.1371/journal.pgen.1007472)
Supplement: S4 Dataset — (PDF) [file pgen.1007472.s032.pdf]

Supplementary dataset 4

Alignment of KS domain

**PKS1-*Metarhizium robertsii***	SKIAIIGMSGRFPESADVEAFWDLLHQGLDVHRPVPPDRFNGELYYDVTG
**PKS1-*Metarhizium anisopliae***	SKIAIIGMSGRFPESADVEAFWDLLHQGLDVHRPVPPDRFNGELYYDVTG
**PKS1-*Metarhizium brunneum***	SKIAIIGMSGRFPESADVEAFWDLLHQGLDVHRPVPPDRFNGELYYDVTG
**PKS1-*Metarhizium guizhouense***	SKIAIIGMSGRFPESADVEAFWDLLHQGLDVHRPVPPDRYNGELYYDVTG
**PKS1-*Metarhizium majus***	SKIAIIGMSGRFPESADVEAFWDLLHQGLDVHRPVPPDRYNGELYYDVTG
**PKS1-*Metarhizium acridum***	SKIAIIGMSGRFPESADVEAFWDLLYQGLDVHRPVPPDRFNGESYYDVTG
**PKS1-*Metarhizium album***	SKIAIIGMSGRFPEAADVEAFWAVLYKGLDVHRPVPKDRYDGELYYDPTG
**PKS-*Colletotrichum fioriniae***	SKIAIVAMSGRFPDAANLDEFWDVLYQGRDVHRQIPEDRFDAVSHYDATG
**PKS-*Trichoderma reesei***	SKIAIVGMSGRFPESADVEAFWDLLYQGLDVHRRVPADRYNAELYYDPTG
**PKS-*Fusarium graminearum***	SKIAIVAMSGRFPDAADLGEFWDLLYKGRDVHRQIPEDRFNAELHYDATG
**PKS2-*Metarhizium robertsii***	SKIAIIGMSGRFPEAADLDSFWSLLEQGVDAYRPVPPDRFDAHAHHDETG
**PKS2-*Metarhizium anisopliae***	SKIAIIGMSGRFPEAADLDSFWSLLEQGVDAYRPVPPDRFDAHAHHDETG
**PKS2-*Metarhizium brunneum***	SKIAIIGMSGRFPEAADLDSFWSLLEQGVDAYRPVPPDRFDAHAHHDETG
**PKS2-*Metarhizium guizhouense***	-KIAIIGMSGRFPEAADLDSFWSLLEQGVDAYRPVPPDRFDAHAHHDETG
**PKS2-*Metarhizium majus***	SKIAIIGMSGRFPEAADLDSFWSLLEQGVDAYRQVPPDRFDAHAHHDETG
**PKS2-*Metarhizium acridum***	SKIAIIGMSGRFPEAADLDSFWSLLVQGVDAYRPVAPDRFDAHAHYDETG
**PKS-*Talaromyces marneffei***	SKIAIIGLSGRFPDAPSPEHFWNLLYQGLDVHREVPPDRWNWKTHVDLTG
**PKS-*Penicillium oxalicum***	SKIAIIGLSGRFPEAQDTEAFWDLLYKGLDVHREVPPDRWDVEAHVDMEG
**PKS-*Aspergillus fumigatus***	SKIAIIGMSGRFPEADGIEAFWDLLYKGLDVHKKVPPERWDVDAHVDLTG
**PKS-*Aspergillus clavatus***	SKIAIIGMSGRFPEADGVEAFWDLLYKGLDVHKTVPPDRWDVDAHVDLTG

<u>**Conserved motif**</u>

DPAQRLXL

**PKS1-*Metarhizium robertsii***	KRKNTCKVMHGCWINDPGLFDAKFFNISPKEAEQSDPGQRLALATAYEAL
**PKS1-*Metarhizium anisopliae***	KRKNTCKVMHGCWINDPGLFDAKFFNISPKEAEQSDPGQRLALATAYEAL
**PKS1-*Metarhizium brunneum***	KRKNTCKVMHGCWINDPGLFDAKFFNISPKEAEQSDPGQRLALATAYEAL
**PKS1-*Metarhizium guizhouense***	KRKNTCKVMHGCWINDPGLFDAKFFNISPKEAEQSDPGQRLALATAYEAL
**PKS1-*Metarhizium majus***	KRKNTCKVMHGCWINEPGLFDAKFFNISPKEAEQSDPGQRLALATAYEAL
**PKS1-*Metarhizium acridum***	KRKNTCKVMHGCWINEPGLFDAKFFNISPKEAEQSDPGQRLALATAYEAL
**PKS1-*Metarhizium album***	KKKNTCKVMHGCWINEPGLFDAKFFNISPKEAEQSDPGQRLALTTAYEAL
**PKS-*Colletotrichum fioriniae***	RRKNTSKVMNGCWIEEPGLFDAKFFNISPKEAEQSDPGQRLALETAYEAL
**PKS-*Trichoderma reesei***	KRKNTSKIMHGCWINEPGLFDAKFFNISPKEAEQSDPGQRLALATAYEAL
**PKS-*Fusarium graminearum***	RRKNTSKVMNGCFIKEPGLFDARFFNMSPKEAEQSDPGQRMALETAYEAL
**PKS2-*Metarhizium robertsii***	RRKNTSKVLGGCWINQPGLFDPKFFSISPKEAEQSDPAQRLALQTAYEAL
**PKS2-*Metarhizium anisopliae***	RRKNTSKVLGGCWINQPGLFDPKFFSISPKEAEQSDPAQRLALQTAYEAL
**PKS2-*Metarhizium brunneum***	RRKNTSKVLGGCWINQPGLFDAKFFSISPKEAEQSDPAQRLALQTAYEAL
**PKS2-*Metarhizium guizhouense***	RRKNTSRVLGGCWINQPGLFDPKFFSISPKEAEQSDPAQRLALQTAYEAL
**PKS2-*Metarhizium majus***	RRKNTSRVLGGCWINQPGLFDAKFFSISPKEAEQSDPAQRLALQTAYEAL
**PKS2-*Metarhizium acridum***	RRKNTSKVLGGCWINQPGLFDPKFFSISPKEAEQSDPAQRLALQTAYEAL
**PKS-*Talaromyces marneffei***	KKRNTSQVPYGCWIPEPGLFDPRFFNMSPREALQADPGQRLALVTAYEAL
**PKS-*Penicillium oxalicum***	NKRNTSKVQYGCWIREPGLFDPRFFNMSPREALQADPAQRLALLTAYEAF
**PKS-*Aspergillus fumigatus***	TKRNTSKVPYGCWINEPGLFDARFFNMSPREALQADPAQRLALLSAYEAL
**PKS-*Aspergillus clavatus***	TKRNTSKVPFGCWINEPGMFDARFFNMSPREALQADPAQRLALLTAYEAL

PKS1-*Metarhizium robertsii*	EAAGVVADRTPSTQRDRVGVFYGMTSDDYREVS---CGQNVDTY------
PKS1-*Metarhizium anisopliae*	EAAGVVADRTPSTQRDRVGVFYGMTSDDYREVS---CGQNVDTY------
PKS1-*Metarhizium brunneum*	EAAGVVADRTPSTQRDRVGVFYGMTSDDYREVS---CGQNVDTY------
PKS1-*Metarhizium guizhouense*	EAAGVVADRTPSTQRDRVGVFYGMTSDDYREVS---CGQNVDTY------
PKS1-*Metarhizium majus*	EAAGVVADRTPSTQRDRVGVFYGMTSDDYREVS---CGQNVDTY------
PKS1-*Metarhizium acridum*	ESAGVVADRTPSTQRDRVGVFYGMTSDDYREVS---CGQNVDTY------
PKS1-*Metarhizium album*	ESAGVVAGRTPSTQRDRVGVFYGMTSDDYREVRASVCKPQCKPFPYTKAN
PKS-*Colletotrichum fioriniae*	EMAGIVPDRTPSTQRDRVGVFYGMTSDDWREVN---SGQNVDTY------
PKS-*Trichoderma reesei*	EMAGIVADRTPSTQRDRVGVFYGMTSDDYREVS---CGQNVDTY------
PKS-*Fusarium graminearum*	EMAGIVPDRTPSTQRDRVGVFYGMTSDDWREVN---SGQNVDTY------
PKS2-*Metarhizium robertsii*	EMAGVVPDRTQSTQRDRVGVFYGMVSDDWREIN---SGQNIDTY------
PKS2-*Metarhizium anisopliae*	EMAGVVPDRTQSTQRDRVGVFYGMVSDDWREIN---SGQNIDTY------
PKS2-*Metarhizium brunneum*	EMAGVVPDRTQSTQRDRVGVFYGMVSDDWREIN---SGQNIDTY------
PKS2-*Metarhizium guizhouense*	EMAGVVPDRTQSTQRDRVGVFYGMVSDDWREIN---SGQNIDTY------
PKS2-*Metarhizium majus*	EMAGVVPDRTQSTQRDRVGVFYGMVSDDWREIN---SGQNIDTY------
PKS2-*Metarhizium acridum*	EMAGMVPDRTQSTQRERVGVFYGM--------------------------
PKS-*Talaromyces marneffei*	EMAGFVPDSTPSTQKDRVGIFYGMTSDDYREIN---SGQDIDTY------
PKS-*Penicillium oxalicum*	EMAGFIPDSTPSTQKNRVGVFYGMTSDDYREIN---SGQDIDTY------
PKS-*Aspergillus fumigatus*	EMAGFVPNSSPSTQRDRVGIFMGMTSDDYREIN---SGQDIDTY------
PKS-*Aspergillus clavatus*	EMAGFIPNSTPSTQRDRVGIFYGMTSDDYREIN---SGQDIDTY------

**Conserved motif**	GPS DTACSSSL
PKS1-*Metarhizium robertsii*	FIPGGNRAFTPGKINYFFKYCGPSVSVDTACSSSLAAIHLACNSIWRNEC
PKS1-*Metarhizium anisopliae*	FIPGGNRAFTPGKINYFFKYCGPSVSVDTACSSSLAAIHLACNSIWRNEC
PKS1-*Metarhizium brunneum*	FIPGGNRAFTPGKINYFFKYCGPSVSVDTACSSSLAAIHLACNSIWRNEC
PKS1-*Metarhizium guizhouense*	FIPGGNRAFTPGKINYFFKYCGPSVSVDTACSSSLAAIHLACNSIWRNEC
PKS1-*Metarhizium majus*	FIPGGNRAFTPGKINYFFKYCGPSVSVDTACSSSLAAIHLACNSIWRNEC
PKS1-*Metarhizium acridum*	FIPGGNRAFTPGKINYFFKYCGPSVSVDTACSSSLAAIHLACNSIWLNEC
PKS1-*Metarhizium album*	LTTGGNRAFTPGKINYFFKYCGPSVSVDTACSSSLAAIHLACNSIWRNEC
PKS-*Colletotrichum fioriniae*	FIPGGNRAFTPGRLNYFFKFSGPSASIDTACSSSLTAIHMACNSLWRNDC
PKS-*Trichoderma reesei*	FIPGGNRAFTPGKINYFFKYCGPSVSVDTACSSSLAAIHLACNSIWRNEC
PKS-*Fusarium graminearum*	FIPGGNRAFTPGRLNYFFKFSGPSASVDTACSSSLAALHLACNSLWRNDC
PKS2-*Metarhizium robertsii*	FIPGGIRAFTPGRINYHFKFSGPSITVDTACSSSLAAIHVACNSLWRGDC
PKS2-*Metarhizium anisopliae*	FIPGGIRAFTPGRINYHFKFSGPSITVDTACSSSLAAIHVACNSLWRGDC
PKS2-*Metarhizium brunneum*	FIPGGIRAFTPGRINYHFKFSGPSITVDTACSSSLAAIHVACNSLWRGDC
PKS2-*Metarhizium guizhouense*	FIPGGIRAFTPGRINYHFKFSGPSITVDTACSSSLAAIHVACNSLWRGDC
PKS2-*Metarhizium majus*	FIPGGIRAFTPGRINYHFKFSGPSITVDTACSSSLAAIHVACNSLWRGDC
PKS2-*Metarhizium acridum*	FTQGGIRAFTPGRINYHFKFSGPSITVDTACSSSLAAIHVACNSLWRGDC
PKS-*Talaromyces marneffei*	FIPGGNRAFTPGRINYHFKFSGPSVSVDTACSSSLSAIHMACNALWKNDC
PKS-*Penicillium oxalicum*	FIPGGNRAFTPGRINYYFKFSGPSVSVDTACSSSLAAIHVACNSLWRNEC
PKS-*Aspergillus fumigatus*	FIPGGNRAFTPGRINYYFKFSGPSVSVDTACSSSLAAIHLACNAIWRNDC
PKS-*Aspergillus clavatus*	FIPGGNRAFTPGRINYYFKFSGPSVSVDTACSSSLAAIHLACNAIWRNDC

Conserved motif	GYCRAD
PKS1-*Metarhizium robertsii*	DTAIAGGTNVMSNPDSFVGLDRGYFLSRTGNCHTFDDDADGYCRADAVGT
PKS1-*Metarhizium anisopliae*	DTAIAGGTNVMSNPDSFVGLDRGYFLSRTGNCHTFDDEADGYCRADAVGT
PKS1-*Metarhizium brunneum*	DTAIAGGTNVMSNPDSFVGLDRGYFLSRTGNCHTFDDEADGYCRADAVGT
PKS1-*Metarhizium guizhouense*	DTAIAGGTNVMSNPDSFVGLDRGYFLSRTGNCHTFDDEADGYCRADAVGT
PKS1-*Metarhizium majus*	DTAIAGGTNVMSNPDSFVGLDRGYFLSRTGNCHTFDDEADGYCRADAVGT
PKS1-*Metarhizium acridum*	DTAIAGGTNVMTNPDSFVGLDRGYFLSRTGNCHTFDDEADGYCRADAVGT
PKS1-*Metarhizium album*	DTAVAGGTNVMSNPDSFVGLDRGHFLSRKGNCNNFDDEADGYCRADAVAT
PKS-*Colletotrichum fioriniae*	DTAIAGGTNVMTNPDNFAGLDRGHFLSRTGNCNTFDDGADGYCRADGVGT
PKS-*Trichoderma reesei*	DTAVAGGTNVMSNPDSFAGLDRGHFLSRTGNCNTFDDAADGYCRADAVGT
PKS-*Fusarium graminearum*	DTAIAGGTNVMTNPDNFAGLDRGHFLSRTGNCNTFDDGADGYCRADGVGT
PKS2-*Metarhizium robertsii*	DTAVAGGVNVLTNPDIFAGLDRGHFLSTTGNCKTFDDDADGYCRADGVGT
PKS2-*Metarhizium anisopliae*	DTAVAGGVNVLTNPDIFAGLDRGHFLSTTGNCKTFDDDADGYCRADGVGT
PKS2-*Metarhizium brunneum*	DTAVAGGVNVLTNPDIFAGLDRGHFLSTTGNCKTFDDDADGYCRADGVGT
PKS2-*Metarhizium guizhouense*	DTAVAGGVNVLTNPDIFAGLDRGHFLSTTGNCKTFDDDADGYCRADGVGT
PKS2-*Metarhizium majus*	DTAVAGGVNVLTNPDIFAGLDRGHFLSTTGNCKTFDDDADGYCRADGVGT
PKS2-*Metarhizium acridum*	DTAVAGGVNVLTNPDIFAGLDRGHFLSTTGNCKTFDDDADGYCRADGVGT
PKS-*Talaromyces marneffei*	DTAIAGGTNVLTNPDNHAGLDRGHFLSRTGNCNTFDDAADGYCRADGVGT
PKS-*Penicillium oxalicum*	DSAVAGGVNILTNPDNHAGLDRGHFLSRTGNCTTFDDGADGYCRADGIGS
PKS-*Aspergillus fumigatus*	DTAISGGVNLLTNPDNHAGLDRGHFLSRTGNCNTFDDGADGYCRADGVGT
PKS-*Aspergillus clavatus*	DSAITGGVNLLTNPDNHAGLDRGHFLSRTGNCNTFDDGADGYCRADGVGS

PKS1-*Metarhizium robertsii*	VILKRLEDAIADHDPILGVISGALTNHSADAVSITRPHSGAQEEIFSKLL
PKS1-*Metarhizium anisopliae*	VILKRLEDAIADHDPILGVISGALTNHSADAVSITRPHSGAQEEIFSKLL
PKS1-*Metarhizium brunneum*	VILKRLEDAIADHDPILGVISGALTNHSADAVSITRPHSGAQEEIFSKLL
PKS1-*Metarhizium guizhouense*	VILKRLEDAIADHDPILGVISGALTNHSADAVSITRPHSGAQEEIFSKLL
PKS1-*Metarhizium majus*	VILKRLEDAIADHDPILGVISGALTNHSADAVSITRPHSGAQEEIFSKLL
PKS1-*Metarhizium acridum*	VILKRLEDAIADHDPILGIISGAYTNHSADSVSITRPHSGAQEEIFSKLL
PKS1-*Metarhizium album*	VVLKRLEDAMADHDPILGVISGAHTNHSAESVSITRPHSGAQEEIFSKLL
PKS-*Colletotrichum fioriniae*	IVLKRLEDAEADNDPILGVIAGAYTNHSAEAVSITRPHAGAQEYIFSKLL
PKS-*Trichoderma reesei*	VVLKRLEDAVADHDPILGVILGAHTNHSAESVSITRPHCGAQEEIFSKIL
PKS-*Fusarium graminearum*	IILKRLEDAEADNDPILGVILGAYTNHSAEAVSITRPHAGAQEYIFSKLL
PKS2-*Metarhizium robertsii*	VILKRLEDAVMDKDPILAVLNSAYTNHSAEAVSITRPHAGAQELIFSKLL
PKS2-*Metarhizium anisopliae*	VILKRLEDAVMDKDPILAILNSAYTNHSAEAVSITRPHAGAQELIFSKLL
PKS2-*Metarhizium brunneum*	VVLKRLEDAVMDKDPILAVLNSAYTNHSAEAVSITRPHAGAQERIFSKLL
PKS2-*Metarhizium guizhouense*	VILKRLEDAVMDKDPILAVLNSAYTNHSAEAVSITRPHAGAQELIFSKLL
PKS2-*Metarhizium majus*	VILKRLEDAVMDKDPILAVLNSAYTNHSAEAVSITRPHAGAQELIFSKLL
PKS2-*Metarhizium acridum*	VILKRLEDAIMDKDPILAVLNSAYTNHSAEAVSITRPHAGAQELIFSKLL
PKS-*Talaromyces marneffei*	VVLKRLEDAIADNDPIQAVIAGAYTNHSAEAVSMTRPHSGAQAFIFDKLL
PKS-*Penicillium oxalicum*	VVLKRLEDAQADNDPIYGVIAGAYTNHSAEAVSITRPHAGAQAFIFDKLL
PKS-*Aspergillus fumigatus*	IVLKRLEDAEADNDPILGVINAAYTNHSAEAVSITRPHVGAQAFIFNKLL
PKS-*Aspergillus clavatus*	IVLKRLEDAEADNDPILGVINAAYTNHSAEAVSITRPHVGAQAFIFNKLL

Conserved motif	EXHGTGTXXGDA
PKS1-*Metarhizium robertsii*	TESGVHPHQVSYIEMHGTGTQAGDATEMTSVLNCFAPSTSPR-RLPHESL
PKS1-*Metarhizium anisopliae*	TESGVHPHQVSYIEMHGTGTQAGDATEMTSVLNCFAPSTSPR-RLPHESL
PKS1-*Metarhizium brunneum*	TESGVHPHQVSYIEMHGTGTQAGDATEMTSVLNCFAPSTSTR-RLPHESL
PKS1-*Metarhizium guizhouense*	TESGVHPHQVSYIEMHGTGTQAGDATEMTSVLNCFAPSTSPR-RLPHESL
PKS1-*Metarhizium majus*	TESGVHPHQVSYIEMHGTGTQAGDATEMTSVLNCFAPSTSPR-RLPHESL
PKS1-*Metarhizium acridum*	TESGVHPHQVSYIEMHGTGTQAGDATEMTSVLNCFAPSTHPR-RLPHESL
PKS1-*Metarhizium album*	CESGVHPHQVSYVEMHGTGTQAGDATEMTSVLNCFAPSTGPR-RLPHESL
PKS-*Colletotrichum fioriniae*	RESGIDPYDVSYVEMHGTGTQAGDATEMSSVLKTFAPTSGRGSRSPDQSL
PKS-*Trichoderma reesei*	TESGVHPHHVSYIEMHGTGTQAGDATEMASVLKVFAPSPTAG-RLPHESL
PKS-*Fusarium graminearum*	RESGTDPYNVSYIEMHGTGTQAGDATEMTSVLKTFAPTSGFGGRLPHQNL
PKS2-*Metarhizium robertsii*	RETGIHPHDVSYIEMHGTGTQAGDATEMSSVLRTFAPDTG---RLSSQTL
PKS2-*Metarhizium anisopliae*	RETGIHPHDVSYIEMHGTGTQAGDATEMSSVLRTFAPDTR---RLSSQTL
PKS2-*Metarhizium brunneum*	RETGINPHDVSYIEMHGTGTQAGDATEMSSVLRTFAPDSG---RLSNQTL
PKS2-*Metarhizium guizhouense*	RETGIHPHDVSYIEMHGTGTQAGDATEMSSVLRTFASDTS---RLSNQTL
PKS2-*Metarhizium majus*	RETGIHPHDVGYIEMHGTGTQAGDATEMSSVLRTFAPGTG---RLSSQPL
PKS2-*Metarhizium acridum*	RETGIHPHDVSYIEMHGTGTQAGDATEMSSVLRTFAPDTG---RLSNQTL
PKS-*Talaromyces marneffei*	NEAGVHPNDVSYIEMHGTGTQAGDAVEMTSVLDTFAPDHG---RKPEQTL
PKS-*Penicillium oxalicum*	NENDYDPKEVSYIEMHGTGTQAGDAVEMTSVLDVFAPDYR---RGPSQSL
PKS-*Aspergillus fumigatus*	NDTNTNPHEIGYVEMHGTGTQAGDAVEMQSVLDVFAPDYR---RGPANSL
PKS-*Aspergillus clavatus*	NDTNTNPHEVSYVEMHGTGTQAGDAVEMQSVLDVFAPDCR---RGPANSL

Conserved motif	GSXKXNXGHXE
PKS1-*Metarhizium robertsii*	HLGSTKANVGHSESASGVSALIKVLLMMEKNIIPPHCGI
PKS1-*Metarhizium anisopliae*	HLGSTKANVGHSESASGVSALIKVLLMMEKNIIPPHCGI
PKS1-*Metarhizium brunneum*	HLGSTKANVGHSESASGVSALIKVLLMMEKNIIPPHCGI
PKS1-*Metarhizium guizhouense*	HLGSTKANVGHSESASGVSALIKVLLMMEKNIIPPHCGI
PKS1-*Metarhizium majus*	HLGSTKANVGHSESASGVSALIKVLLMMEKNIIPPHCGI
PKS1-*Metarhizium acridum*	HLGSTKANVGHAESASGVSALIKVLLMMEKNIIPPHCGI
PKS1-*Metarhizium album*	HLGSAKANVGHSESASGVTSLIKVLLMMEKNMIPPHCGI
PKS-*Colletotrichum fioriniae*	HLGSAKANVGHAESASGVVALIKTLLMLQNSTIPPHCGI
PKS-*Trichoderma reesei*	YLGSTKANVGHSESASGVTALIKVLLMMQKSLIPPHCGI
PKS-*Fusarium graminearum*	HLGSVKANVGHGESASGIIALIKTLLMMEKNMIPPHCGI
PKS2-*Metarhizium robertsii*	HLGSAKSNVGHGEAASGVTSLIKVLLMMKHNTIPPHCGI
PKS2-*Metarhizium anisopliae*	HLGSAKSNVGHGEAASGVTSLIKVLLMMKHNTIPPHCGI
PKS2-*Metarhizium brunneum*	HLGSAKSNVGHGESASGVTSLIKVLLMMKHNMIPPHCGI
PKS2-*Metarhizium guizhouense*	HLGSAKSNVGHGESASGVTSLIKVLLMMKHNTIPPHCGI
PKS2-*Metarhizium majus*	HLGSAKSNIGHGESASGVTSLIKVLLMMKHNTIPPHCGI
PKS2-*Metarhizium acridum*	HLGSAKSNVGHGESASGVTSLIKVLLMMKHNTIPPHCGI
PKS-*Talaromyces marneffei*	HIGSAKSNIGHGESASGVTALIKVLMMMRKSTIPPHCGI
PKS-*Penicillium oxalicum*	HLGSAKSNIGHGESASGVCALLKVLMMMRKNTIPPHCGI
PKS-*Aspergillus fumigatus*	YLGSAKSNIGHGESASGVTSLVKVLLMLKQNMIPPHCGI
PKS-*Aspergillus clavatus*	HLGSAKSNVGHGESASGVTSLVKVLLMMKQNMIPPHCGI

Alignment of AT domain

**PKS1-*Metarhizium robertsii***	FVFSGQGSQYSAMGQHLLH-FTIFRDEVHAYDILAQRHGFPSIMPLIDGS
**PKS1-*Metarhizium anisopliae***	FVFSGQGSQYSSMGQHLLH-FTIFRDEVHAYDILAQRHGFPSIMPLIDGS
**PKS1-*Metarhizium brunneum***	FVFSGQGSQYSAMGQHLLH-FTIFRDEVHAYDILAQRHGFPSIMPLIDGS
**PKS1-*Metarhizium guizhouense***	FVFSGQGSQYSAMGQHLLH-FTIFRDEVHAYDILAQRHGFPSIMPLIDGS
**PKS1-*Metarhizium majus***	FVFSGQGSQYSAMGQHLLH-FTIFRDEVHAYDILARRHGFPSIMPLIDGS
**PKS1-*Metarhizium acridum***	FVFSGQGSQYSAIGRHLLH-FTIFRDEVNSYDILAQRHGFPSIMPLIDGS
**PKS1-*Metarhizium album***	FVFSGQGSQYGAMGKHLLH-YTIFRGEIDSYDILAQRHGFPSIMPLVDGS
**PKS-*Trichoderma reesei***	FAFSGQGSQYSAMGQHLLQ-FASFRDEIDSYDRLAQRHGFPSILPLIDGS
**PKS-*Colletotrichum fioriniae***	FVFSGQGAQYSGMGKEYFNSFSLFRSEIQSYDRIGQSQGFPSILPLITGE
**PKS-*Fusarium graminearum***	FVFSGQGAQYRGMGKEYFTSFTAFRSEIMSYDSIAQAQGFPSILPLIRGE
**PKS2-*Metarhizium robertsii***	FVFSGQGAQYAGMGRHLFHNNHTFRTQILACNQICLSHGFPSILEIFKQD
**PKS2-*Metarhizium anisopliae***	FVFSGQGAQYAGMGRHLFQNNHTFRTQILACNQICLSQGFPSILEIFKQD
**PKS2-*Metarhizium brunneum***	FVFSGQGAQYAGMGRHLFQNNHTFRTQILACNQICLSQGFPSILEIFKQD
**PKS2-*Metarhizium guizhouense***	FVFSGQGAQYAGMGRHLFQNNHTFRTQILACNRICLSQGFPSILEIFTQD
**PKS2-*Metarhizium majus***	FVFSGQGAQYAGMGRHLFQNNHAFRTQILACNQICRSQGFPSILEIFEQD
**PKS2-*Metarhizium acridum***	FVFSGQGAQYAGMGRHLFQNNETFRTQVLACNQICLSQGFPSILDIFNQG
**PKS-*Talaromyces marneffei***	FIFTGQGAQYAGMGKQFFESFSQFRSDILRFNGIAQSQGFPSFLPLIDGS
**PKS-*Penicillium oxalicum***	FVFTGQGAQYTGMGKQLYEDCATFRSTIHRLDCIAQSQGFPSILPLIDGS
**PKS-*Aspergillus fumigatus***	FVFTGQGAQYSGMGKQLYEDCATFRSIIHRFDCIARSQGFPSIIHLIDGS
**PKS-*Aspergillus clavatus***	FIFTGQGAQYAGMGKDLFHHNKQFRDNVEHLDRIALSHGFPSIMPLIDGS

**PKS1-*Metarhizium robertsii***	VDIEDLEPLVVQLGTVCVQMALASLWMALGMRPAYVVGHSLGHYAALKVA
**PKS1-*Metarhizium anisopliae***	VDIEDLEPLVVQLGTVCVQMALASLWMALGMRPAYVVGHSLGHYAALKVA
**PKS1-*Metarhizium brunneum***	VDIEDLEPLVVQLGTVCVQMALASLWMALGMRPAYVVGHSLGHYAALKVA
**PKS1-*Metarhizium guizhouense***	VDIEDLEPLVVQLGTVCVQMALASLWMALGMRPAYVVGHSLGHYAALKVA
**PKS1-*Metarhizium majus***	VDIEDLEPLVVQLGTVCVQMALASLWMALGMRPAYVVGHSLGHYAALKVA
**PKS1-*Metarhizium acridum***	VDIEDLEPLVVQLGTVCVQMALASLWIAFGMRPAYVVGHSLGHYAALKVA
**PKS1-*Metarhizium album***	VAIEHLEPLVVQLGTVCVQMALASLWIAFGMQPSYVVGHSLGHFAALKVS
**PKS-*Trichoderma reesei***	SNIDDLEPLVVQLGTTCVQMALASFWMSLGMQPAYVVGHSLGHYAALKVA
**PKS-*Colletotrichum fioriniae***	AEVESSSPVETQLSLACLQMALAKLWKSFGIEASFVLGHSLGHYAALNVA
**PKS-*Fusarium graminearum***	VEADSLSPVEIQLGLTCLQMALAKLWKSFGVEPGFVLGHSLGHYAALHVA
**PKS2-*Metarhizium robertsii***	VDMNSLEPLLVQLGTTCLQMSLVAFWKSLGVTPDFCIGHSLGEYAALQAA
**PKS2-*Metarhizium anisopliae***	VDMNSLEPLLVQLATTCLQMSLVSFWKSLGVTPDFCIGHSLGEYAALQAA
**PKS2-*Metarhizium brunneum***	VDMNSLEPLVVQLATTCLQMSLVSFWKSLGVTPDFCIGHSLGEYAALQAA
**PKS2-*Metarhizium guizhouense***	VDMNSLEPLVFQLGTTCLQTSLVSFWKSLGVTPDFCIGHSLGEYAALQAA
**PKS2-*Metarhizium majus***	VDVNSLEPLLVQLGTTCLQMSLVSFWKSLGVTPDFCIGHSLGEYAALQAA
**PKS2-*Metarhizium acridum***	VEMNNLEPLVVQLGTTCLQMSLVSFWKSLGVTPDFCIGHSLGEYAALQAA
**PKS-*Talaromyces marneffei***	MPIEEMSPVITQLGTTCLQMALANLWASWETRPTFVMGHSLGEYAALYVS
**PKS-*Penicillium oxalicum***	MPVEELSPVVTQLGTTCLQMALVDYWKGLGVTPAFVLGHSLGDYAALNSA
**PKS-*Aspergillus fumigatus***	VPVEGLSPVVTQLGTTCLQMALVDYWRGLGVSPAFVLGHSLGDYAALNAA
**PKS-*Aspergillus clavatus***	VPVEELSPVVTQLGTTCLQMALTKLWISLGVTPSFVLGHSLGEYAALNAA

**PKS1-*Metarhizium robertsii***	GVLTASDTIYLVAMRARLLQNKCSRGSHAMLAIRSSAAEIQAHLDEGIHD
**PKS1-*Metarhizium anisopliae***	GVLTASDTIYLVAMRARLLQNKCSRGSHAMLAIRSSVAEIQAHLDEGIYD
**PKS1-*Metarhizium brunneum***	GVLTASDTIYLVATRARLLQNKCSRGSHAMLAIRSSAAEIQAHLDEGIHD
**PKS1-*Metarhizium guizhouense***	GVLTASDTIYLVAMRARLLQNKCSRGSHAMLAIRSSAAAIQAHLDEGIHD
**PKS1-*Metarhizium majus***	GVLTASDTIYLVAMRARLLQNKCSRGSHAMLAIRSSADEIQAHLDEGIHD
**PKS1-*Metarhizium acridum***	GVLTASDTIYLVAMRARLLQNKCSRGSHTMLAIRSSADEMQAYLDEDIYD
**PKS1-*Metarhizium album***	GALTASDTIYLVGMRARLLQNKCSIGSHAMLAVRSSAEDVQAYLDADVHD
**PKS-*Trichoderma reesei***	GVLTASDTIYLVGMRARLLQEKCSRGSHAMLAIRSSVDQIQPYLDAALHD
**PKS-*Colletotrichum fioriniae***	GVLSASDTIYLTGTRAQLLLDNCEAGSHAMLAVRASVLQIRQFLNANIHE
**PKS-*Fusarium graminearum***	GVLSANDTIYLTGIRAQLLVDKCQAGTHSMLAVRASLLQIQQFLDANIHE
**PKS2-*Metarhizium robertsii***	GVLSVSDTIYLTGIRARMLQEKCSAGSHAMLAVRAPLARVNALLDPAIHE
**PKS2-*Metarhizium anisopliae***	GVLSVSDTIYLTGIRARMLQEKCSAGSHAMLAVRAPLARVNALLDPAIHE
**PKS2-*Metarhizium brunneum***	GVLSVSDTIYLTGIRARMLQEKCSAGSHAMLAVRAPLARVNALLDPAIHE
**PKS2-*Metarhizium guizhouense***	GVLSVSDTIYLTGIRARMLQEKCSAGSHAMLAVRAPLARVNALLDPGIHE
**PKS2-*Metarhizium majus***	GVLSVSDAIYLTGTRARMLQEKCSAGSHAMLAVRAPLARVNALLDPAIHE
**PKS2-*Metarhizium acridum***	GVLSVSDTIYLTGIRARLLQKKCSAGSHAMLAVRAPLAQVHGFFNPSIHE
**PKS-*Talaromyces marneffei***	GTLTAFDTIYLCGRRAQLLEGKCKVGTHAMLAVRSALSEITPFLNPGIHE
**PKS-*Penicillium oxalicum***	GVLSTSDTIYLCGRRAQLLTQQCQMGTHAMLAVKAAVSEIQHLLDPDVHA
**PKS-*Aspergillus fumigatus***	GVLSTSDTIYLCGRRAQLLTEQCQMGTHAMLAVKAALSDIQHLLDAEAHA
**PKS-*Aspergillus clavatus***	GILTNSDTIFLAGRRAQLLTEQCKVGTHAMLAVKSSVAQVKQFLDDGAAE

**PKS1-*Metarhizium robertsii***	IACINGPQDTVVSGCIDDIDRLSQKLMDKGIKATRVNVPFAFHSAQVDPI
**PKS1-*Metarhizium anisopliae***	IACINGPQDTVVSGCIDDIDRLSQKLMDKGIKATRVNVPFAFHSAQVDPI
**PKS1-*Metarhizium brunneum***	IACINGPQDTVVSGCIDDIDRLSQKLMDKGIKATRVNVPFAFHSAQVDPI
**PKS1-*Metarhizium guizhouense***	IACINGPQDTVVSGCIDDIDRLSQKLMDKGIKATRVNVPFAFHSAQVDPI
**PKS1-*Metarhizium majus***	IACISGPQDTVVSGCIDDIDRLSQKLTDKGIKATRVNVPFAFHSAQVDPI
**PKS1-*Metarhizium acridum***	IACINGPQDTVVSGCIDDIDRLSQKLMDNRIKVTRVNVPFAFHSAQVDPI
**PKS1-*Metarhizium album***	IACINSPQDTVVSGRVDDIDRLSERLLDKGIKATRVNVPFAFHSSQVEPI
**PKS-*Trichoderma reesei***	VACINGPQDTVVSGCVDEISSLAQKLTDNGVKATRVEVPFAFHSAQVDPV
**PKS-*Colletotrichum fioriniae***	VACINGPREVVISGKVADVNQLAERLAADNIKATRVQVPYAFHSAQVDPI
**PKS-*Fusarium graminearum***	VACVNGPREVVISGRVADIDQLVGLLSADNIKATRVKVPFAFHSAQVDPI
**PKS2-*Metarhizium robertsii***	VTCLNGPQDVVIGGRVADVEELEKELAKQDIKAVKVSVPFAFHSTQVEPI
**PKS2-*Metarhizium anisopliae***	VTCLNGPQDVVIGGRVADVEALEKELAKQDIKAVKVSVPFAFHSTQVEPI
**PKS2-*Metarhizium brunneum***	VTCLNGPQDVVIGGRVADVEELERELAKQDIKAVKVSVPFAFHSTQVEPI
**PKS2-*Metarhizium guizhouense***	VTCLNGPHDVVIGGRVADVEDLEKELAKEDIKAVKVSVPFAFHSTQVDPI
**PKS2-*Metarhizium majus***	VTCLNGPRDVVVGGRVADVEELEKELAKQDVKAAKVSVPFAFHSTQVDPI
**PKS2-*Metarhizium acridum***	VTCLNSPHDVAIGGLVADIEDLERELAENNIEAVKVSVPFAFHSTQVEPI
**PKS-*Talaromyces marneffei***	VACINAPGETVISATSQNIDALSEQLVAAGIKVTKLKVPFAFHSSQVEPI
**PKS-*Penicillium oxalicum***	VACINGPTETVISGLSGRIDELAQQCSSQNLKSTKLKVPFAFHSAQVDPI
**PKS-*Aspergillus fumigatus***	VACINGPTETVISGLSGHIDELAEQLTGQSLKSTKLKVPFAFHSAQVTPI
**PKS-*Aspergillus clavatus***	IACINAPSETVISGAVGKIEQLAEKLTNEGFKATKLKVPFAFHSAQVEPI

**PKS1-*Metarhizium robertsii***	LDELEAIASQVEFHAPRVAIGCPLLGKTFTAGETPSLEAKHIRRHCRETV
**PKS1-*Metarhizium anisopliae***	LDELEAIASQVEFHAPRVAVGCPLLGKTFKAGETPSLEAKHIRRHCRETV
**PKS1-*Metarhizium brunneum***	LDELEAVASQVEFHAPRVAIGCPLLSKTFKAGETPSLEAKHIRRHCRETV
**PKS1-*Metarhizium guizhouense***	LDELEAIASQVEFHTPRVAIGCPLLGRTFKAGETPSIEANHIRRHCRETV
**PKS1-*Metarhizium majus***	LDELEATASQVEFHAPRVAIGCPLLGKTFKAGETPSLEANHIRRHCRETV
**PKS1-*Metarhizium acridum***	LDELEAIASQVEFHTPRVAIGCPLLGKTFVAGETSSLGADHIKRHCRETV
**PKS1-*Metarhizium album***	LDELGAIASQVKFHSPCVPIGCPLQGKSLLPGETLTSEASHVKRHCRQTV
**PKS-*Trichoderma reesei***	LDELEAIASHVTFHSPRVPIGCPLLGKTFHIGETPSFDAKHISRHCREAV
**PKS-*Colletotrichum fioriniae***	LSDLDVAASRVIFHSPQIPVLCALEASIFRPGDHGSIGPLHIQRHCRETV
**PKS-*Fusarium graminearum***	LSDLDTAASRVTFHSPQIPVLCALDSSVISPGNHGVIGPLHLQRHCRETV
**PKS2-*Metarhizium robertsii***	LGEFCDAARGVPFQTQNIPVISTLLGEVVQPEATGVFGPGYLKRHCREPV
**PKS2-*Metarhizium anisopliae***	LGEFCDAARGVPFQTQTIPVVSTLLGEVVQPEAAGVFGPGYLKRHCREPV
**PKS2-*Metarhizium brunneum***	LGEFCDAARGVPFQTQTIPVISTLLGEVVHPETTGVFGPGYLKRHCREPV
**PKS2-*Metarhizium guizhouense***	LGEFCAAARGVPFQTQNIPVISTLLGEVVQPETTGVFGPEYLKRHCREPV
**PKS2-*Metarhizium majus***	LGEFCAAARGVPFQTQSMPVISTLLGEVVQPETTGVFGPEYLKRHCREPV
**PKS2-*Metarhizium acridum***	LDEFCAAAESVQFQTQRIPVISTLLGEVVLPEAKAVFGPEYLKRHCREPV
**PKS-*Talaromyces marneffei***	LEELTNAAKGVKFHKPSIPLVSPLLGEVINERNYEQLGGKYLSRHCRETV
**PKS-*Penicillium oxalicum***	LESFEESAQGVIFHEPAVPFVSALNGEVITESNYSVLGPTYMVKHCREAV
**PKS-*Aspergillus fumigatus***	LDPFEDKAQGVVFHKPSVPFVSALTGEVITEENYEVLGPRYMVKHCRETV
**PKS-*Aspergillus clavatus***	LESLAEIAQGVIFRKPTIPYVSALLGNVIDESNAHMLEASYLTRHCRETV

**PKS1-*Metarhizium robertsii***	NFLDVLRSAKDDGFVSE--KTAWIEIGPHTVCSNLVKANINQDITAVPSL
**PKS1-*Metarhizium anisopliae***	NFLDVLRSAKDDGFVSD--KTAWIEIGPHTVCSNLVKANINQDITAVPSL
**PKS1-*Metarhizium brunneum***	NFLDVLRSAKDDGLVSE--KTAWIEIGPHTVCSNLVKANINQDITAVPSL
**PKS1-*Metarhizium guizhouense***	NFFDVLRSAKDEGFVSE--KTAWIEIGPHTVCSNLVKANINQDIVAVPSL
**PKS1-*Metarhizium majus***	NFLDVLRSAKDDGFVSE--KTAWIEIGPHTVCSKLVKANISQDIVAVPSL
**PKS1-*Metarhizium acridum***	NFRDILRSAKGDGLISE--KTAWIEIGPHTVCSTLLRANINQDIVAVPSL
**PKS1-*Metarhizium album***	NFRGILQSAKSDGLVSE--KTAWIEIGPHTVCSMLVKANINHDVTAVPSL
**PKS-*Trichoderma reesei***	NFRGILQSAKEEGVISE--KTTWIEVGPHTVCSNLVKANLGQDITAVPSL
**PKS-*Colletotrichum fioriniae***	NFEGALKAAEREGLITASGSTLWIEIGPHTVCSTFLKASLG---------
**PKS-*Fusarium graminearum***	NFEGALHAAEREKIINKT-STLWIEIGPHVVCSTFLKSS-----------
**PKS2-*Metarhizium robertsii***	NFAAAVEAARDANVIHA--GTVFVEIGPHPVCLALLKSNMGPDAVTLASL
**PKS2-*Metarhizium anisopliae***	NFAAAVQAARDANVIHA--GTVFVEIGPHPVCLALLKSNMGPDAVTLASL
**PKS2-*Metarhizium brunneum***	NFAAAVQAARDANLIHA--GTVFVEIGPHPVCSALLKSNMGPDAVTLASL
**PKS2-*Metarhizium guizhouense***	NFAAAVQAGRDANLIHA--GTVFVEIGPHPVCLALLKSNMGPDAVTLASL
**PKS2-*Metarhizium majus***	DFAAAVRAGRDANLIHA--GTVFVEIGPHPVCLALLKSNMGPDAVTLASL
**PKS2-*Metarhizium acridum***	NFTAAAQAGKDANIINS--ASVFIEIGPHTVCSALLKSNIGPNAVTLPSL
**PKS-*Talaromyces marneffei***	NFLAAIEASRHAKLMSD--KTVWIEIGSHTICSGMIKSTLGPQANTVASL
**PKS-*Penicillium oxalicum***	NFLGALEATRHAKLMDD--ATLWVEVGSHPICSGMIKSTFGPQATTVPSL
**PKS-*Aspergillus fumigatus***	NFLGGLEATRHAKLMNE--TTVWLEVGSHPICSGMIKSTFGPQATTVASL
**PKS-*Aspergillus clavatus***	NFLGALEATRHASLMND--KSLWVEIGSHPVCSGMVKSTFGPQAATIPSL

**PKS1-*Metarhizium robertsii***	MRNKDGWQVL-------------
**PKS1-*Metarhizium anisopliae***	MRNKDGWQVLA------------
**PKS1-*Metarhizium brunneum***	MRNKDGWQVLA------------
**PKS1-*Metarhizium guizhouense***	MRNKDGWQVLA------------
**PKS1-*Metarhizium majus***	MRNKDGWQVLA------------
**PKS1-*Metarhizium acridum***	MRNKDGWQV--------------
**PKS1-*Metarhizium album***	MRSHDGWQVL-------------
**PKS-*Trichoderma reesei***	VRKKDG-----------------
**PKS-*Colletotrichum fioriniae***	-----------------------
**PKS-*Fusarium graminearum***	-----------------------
**PKS2-*Metarhizium robertsii***	HRKD-------------------
**PKS2-*Metarhizium anisopliae***	HR---------------------
**PKS2-*Metarhizium brunneum***	HRK--------------------
**PKS2-*Metarhizium guizhouense***	HR---------------------
**PKS2-*Metarhizium majus***	HR---------------------
**PKS2-*Metarhizium acridum***	HRKDDGWKVLADTL---------
**PKS-*Talaromyces marneffei***	RRNEDSW----------------
**PKS-*Penicillium oxalicum***	RRDDDPWKILSNSLSTLHLAGVE
**PKS-*Aspergillus fumigatus***	RRDEDPWKIISNSLSTLHLAGVE
**PKS-*Aspergillus clavatus***	RRQEDTWKVFSNSLSALYTSGI-

Alignment of PP-Binding domain

**PKS1-*Metarhizium robertsii***	K---IVAEEIGIPSASV-DNGLVFADYGVDSLLSLSISGR
**PKS1-*Metarhizium anisopliae***	K---IVAEEIGIPSASV-DNGLVFADYGVDSLLSLSISGR
**PKS1-*Metarhizium brunneum***	K---IVAEEIGIPSAGV-DNGLVFADYGVDSLLSLSISGR
**PKS1-*Metarhizium guizhouense***	K---IVSEEIGIPSASV-DNGLVFADYGVDSLLSLSISGR
**PKS1-*Metarhizium majus***	K---IVSEEIGIPSASV-DNGLVFADYGVDSLLSLSISGR
**PKS1-*Metarhizium acridum***	ALFKIVSEEIGIPSASV-QNDLVFADYGVDSLLSLSISGR
**PKS1-*Metarhizium album***	AVLRILSEEIGIPLTSL-QYDLVFADYGVDSLLSLTISGR
**PKS-*Trichoderma reesei***	AVIKILSEEIGIPLGSI-QDDLAFADYGVDSLLSLTISGR
**PKS-*Colletotrichum fioriniae***	PLLRILSEEIGLSLSDLNDDDLDFADHGVDSLLSLTLTGR
**PKS-*Fusarium graminearum***	PLLRILSEEIGLGLDVLSDDELDFADHGVDSLLSLTITGR
**PKS2-*Metarhizium robertsii***	RILAILSKEVGLSMETL-TDDLVFADYGVDSLLSLTITGR
**PKS2-*Metarhizium anisopliae***	RILAILSKEVGLSMETL-TDDLVFTDYGVDSLLSLTITGR
**PKS2-*Metarhizium brunneum***	RILAILSEEVGLSMEIL-TDDLVFADYGVDSLLSLTITGR
**PKS2-*Metarhizium guizhouense***	RILAILSEEVGLSLETL-TDDLVFADYGVDSLLSLTITGR
**PKS2-*Metarhizium majus***	I-LVMLAEEVGLSLETL-TDDLVFADYGVDSLLSLTITGR
**PKS2-*Metarhizium acridum***	RILVILAEEVGLSLETL-TDDLVFADYGVDSLLSLTITGR
**PKS-*Talaromyces marneffei***	I----LAGEVGLSEAEL-SDDLVFSDYGVDSLLALTITGK
**PKS-*Penicillium oxalicum***	L--NILAEEVGLSTSEM-TDDLNFADYGVDSLLSLTVTGR
**PKS-*Aspergillus fumigatus***	L--EILAAEVGLSEAEM-TDSLNFADYGVDSLLSLTVTGR
**PKS-*Aspergillus clavatus***	L--EILAAEVGLSESEM-TDGLNFADYGVDSLLSLTVTGR

**PKS1-*Metarhizium robertsii***	LREELDLDVESSAFETCATLADLAAQ-
**PKS1-*Metarhizium anisopliae***	LREELDLDVESSAFETCATLADLAAHL
**PKS1-*Metarhizium brunneum***	LREELDLDVESSAFETCATLADLAAHL
**PKS1-*Metarhizium guizhouense***	LREELDLDVESSAFETCATLADLATHL
**PKS1-*Metarhizium majus***	LREELDLDVESSAFETCATLADLATHL
**PKS1-*Metarhizium acridum***	LREELDLDVESSVFETCATLADLATHL
**PKS1-*Metarhizium album***	LREELDLDIESSVFETCATVGDFAVH-
**PKS-*Trichoderma reesei***	LREELDLDVESSVFEACATFADFIAHL
**PKS-*Colletotrichum fioriniae***	MREDLGIDVDSSAFITCPTLGQLKKF-
**PKS-*Fusarium graminearum***	MREELGLDVESTAFMNCPTLGSF----
**PKS2-*Metarhizium robertsii***	IREELGLDMDSSIFTHYSTLGELKAFL
**PKS2-*Metarhizium anisopliae***	IREELGLDMDSSIFTHYSTLGELKAFL
**PKS2-*Metarhizium brunneum***	IREELGLDMDSSIFTHYSTLGELKAFL
**PKS2-*Metarhizium guizhouense***	IREELDLDMDSSIFTHYSTLGELKEF-
**PKS2-*Metarhizium majus***	IREELGLDMDSSIFTHYSTLGELKAFL
**PKS2-*Metarhizium acridum***	IREELDLDVDSSTFTNCSTLGEL----
**PKS-*Talaromyces marneffei***	FREQLNLDFESSIFMDYPTVKDFK---
**PKS-*Penicillium oxalicum***	YREEIGLDLDSSVFVDQPTIKDFKQM-
**PKS-*Aspergillus fumigatus***	YREELNLDLESSVFMDYPTIKDFKAYL
**PKS-*Aspergillus clavatus***	YREELNLDFESSVFMDYPTIKDFKAYL

Alignment of PS-DH domain

```
PKS1-Metarhizium robertsii       H----------------------VVNGNRVCSSSLYTDFGVTLGSYILEKY
PKS1-Metarhizium anisopliae      H----------------------VVNGNRVCSSSLYTDFGVTLGSYILEKY
PKS1-Metarhizium brunneum        L--------------------------------------------------
PKS1-Metarhizium guizhouense     H----------------------VVNGNRVCSSSLYTDFGVTLGSYILEKY
PKS1-Metarhizium majus           H----------------------VVNGNRVCSSSLYTDFGVTLGSYILEKY
PKS1-Metarhizium acridum         Y---------------------------------------------ILETY
PKS1-Metarhizium album           H----------------------VVNGNRVCTSSLYADFGVTLGTYILDKY
PKS-Trichoderma reesei           H----------------------VVNGNRVCTSSLYTDFGVTLGNYILEKH
PKS-Colletotrichum fioriniae     A--------------------------------------------------
PKS-Fusarium graminearum         H----------------------RVNGVKVCTSSVYADVGLTLGKYILDNY
PKS2-Metarhizium robertsii       Q--------------------GHKVNGEMLCTSSLYAEIGMTLGRQLLEKY
PKS2-Metarhizium anisopliae      V--TIVVQSDFGSARLADVAQGHKVNGEMLCTSSLYAEIGMTLGRHLLEKY
PKS2-Metarhizium brunneum        V--TIVVQSDFGSARLADVAQGHKVNGEMLCTSSLYAEIGMTLGRQLLEKY
PKS2-Metarhizium guizhouense     Q--------------------GHKVNGEMLCTSSLYAEIGMTLGRQLLEKH
PKS2-Metarhizium majus           R--------------------GHKVNGEMLCTSSLYAEIGMTLGRQLLEKY
PKS2-Metarhizium acridum         E--------------------GHKVNGEMLCTSSLYAEIGMTLGRQLLEKY
PKS-Talaromyces marneffei        K-ATVITETDISDPDLCPVILGHKVNGTPLCPSSLYADIAQTLAEYLIDNF
PKS-Penicillium oxalicum         NSASVLIENDIADPELNRVIQGHKVNGAALCPSSLYADIAQTLAEFLVDKY
PKS-Aspergillus fumigatus        P-------------ELNRVIQGHKVNGVALTPSSLYADIAQTLVDHLITKY
PKS-Aspergillus clavatus         P-------------DLNRVIQGHKVNGVALTPSSLYADIAQTLVDHLITTY

PKS1-Metarhizium robertsii       RPDLQGHAVDVQDMVVNKALVHKE-----GPTMLLRIDVVLDTTDSKAASM
PKS1-Metarhizium anisopliae      RPDLQGHAVDVQDMVVNKALVHKE-----GPTMLLRIDVVLDTTDSKAASM
PKS1-Metarhizium brunneum        ----QGHAVDVQDMVVNKALVHKE-----GPTMLLRIDVVLDTTDSKAASM
PKS1-Metarhizium guizhouense     RPDLQDHAVDVQDMVVNKALVHKE-----GPTMLLRIDVVLDMTDSKAASM
PKS1-Metarhizium majus           RPDLQDHAVDVQDMVVNKALVHKE-----GPTMLLRIDVVLDMTDSKAASM
PKS1-Metarhizium acridum         RPDLKDHSVDVQDMVVNKALVHKE-----GSTMLLRINVILDMTDGKAASM
PKS1-Metarhizium album           RADLKDHSVDVQEMVVNKPLVHKE-----GLPMLLRIDVVHDMTASKSATM
PKS-Trichoderma reesei           RVDLKDHSVDVQKMVVHKALVHKE-----GQPMLLRIEVTHDVTLGKDASM
PKS-Colletotrichum fioriniae     --------VNVHHMQVYKPLILKEDASGTSLPTPFSIEVKYRID-TLTASM
PKS-Fusarium graminearum         RTDLEGYAVDVHGIEVHKPLLLKEDMNGTPQATPFRIEVRYPIQ-STTALM
PKS2-Metarhizium robertsii       RPDLDGYSTEIKDMSVDKPLILKDE----NKRTLFRAEVVHDKS-THTATM
PKS2-Metarhizium anisopliae      RPDLDGYSTEIKDMSVDKPLILKDE----NKQTLFRAEVVHDKT-THTATM
PKS2-Metarhizium brunneum        RPDLQGYSTEIQDMSVDKPLILKDE----NKQTLFRAEVVHDKS-THTASM
PKS2-Metarhizium guizhouense     RPDLQGYSTEIQDMSVDKPLILKDQ----NKQTLFRAEVVHDKS-THTAAM
PKS2-Metarhizium majus           RPDLQGSSTEIQDMSVDRPLILKDE----NKQTLFRAEVVHDKS-TPTATM
PKS2-Metarhizium acridum         RPDLQGYSTEIEDMSVDKPLILKEK----NKETLFRAEVVHDKS-TLTAAM
PKS-Talaromyces marneffei        KPELKGVGLDVADMAVPKPLIYKN-----AGPQLFRAAATADWD-ARQVSM
PKS-Penicillium oxalicum         KPEWKDRGFDVCDVVVPKPLIAK------GGKQLFRVSATATWA-EESAKM
PKS-Aspergillus fumigatus        KPEYQGLGLDVCDMTVPKPLIAK------SGDQFFRVSAVMSWA-EQKASV
PKS-Aspergillus clavatus         KPELQGLGLDVCDMTVPKPLIAK------SGDQFFRISAVMNWA-DKKAAV
```

**PKS1-*Metarhizium robertsii***	SIYSVNSK-GNKTADHAQSSLHFEQPKVWLKSWDSTQYYVERSIEWLKEKA
**PKS1-*Metarhizium anisopliae***	SIYSVNSK-GNKTADHAQSSLHFEQPKVWLKSWDSTQYYVERSIEWLKEKA
**PKS1-*Metarhizium brunneum***	SIYSVNSK-GNKTADHAQSSLHFEQPKVWLKSWDSTQYYVERSIEWLKEKA
**PKS1-*Metarhizium guizhouense***	SIYSVNSK-GNKTADHAQSSLHFEQPKVWLKSWDSTQYYVERSIEWLKEKA
**PKS1-*Metarhizium majus***	SIYSVNSK-GNKTAEHAQSSLHFERPKVWLKSWDSTQYYVERSIEWLKEKA
**PKS1-*Metarhizium acridum***	SIYSVNSK-GDKTADHAQSSLHFEQPKVWLRNWDSTQYYVERSIEWLKEKA
**PKS1-*Metarhizium album***	SIYSINAK-GNKTVDHAKSSLCFGESRAWLRSWDSTQYYVERSIEWLKEKA
**PKS-*Trichoderma reesei***	SIYSINPM-GKKTADHAQCTLSFGQPKVWLKNWDGTLYYVERSIEWLKGKA
**PKS-*Colletotrichum fioriniae***	TIRSGGSHHDGLGTKHVDCELRFENPKDWEAEWDRQAYLIKRSIEYLESRA
**PKS-*Fusarium graminearum***	SISTTGP--NGQHIKHANCELRLEHPSQWEAEWDRQAYLINRSVNYLLQRS
**PKS2-*Metarhizium robertsii***	SIYSVDSA-GNKTVDHARCLLRFADPTSWLDEWERTHYLIDRSVRWLEERA
**PKS2-*Metarhizium anisopliae***	SIYSVDSA-GNKTVDHARCLLRFADPTSWLDEWERTYYLIDRSVRWLEERA
**PKS2-*Metarhizium brunneum***	SIYSVDSA-GNKTVDHARCLLRFADPNSWLEEWERTYYLIDRSVHWLEERA
**PKS2-*Metarhizium guizhouense***	SIYSVDSA-GNKTVDHARCLLCFADPNSWLDEWERTYYLIDRSVRWLEARA
**PKS2-*Metarhizium majus***	SIYSVDSA-GNKTVDHARCLLRFADPTSWLDEWERTYYLIDRSVRWLEARA
**PKS2-*Metarhizium acridum***	SIYSVDSA-GKKTVDHAHCILRFADPKSWLDEWERTYYSIERSVRWLEARV
**PKS-*Talaromyces marneffei***	QIYSVTPE-GRKMTDHASCIIKFFDTKAAREEWKRNAYLIQRSVDRLFESA
**PKS-*Penicillium oxalicum***	QIWSVTPE-GKKILDHASCTIKFFDTAAAATEWKRSAYLIKRSIEHLKQST
**PKS-*Aspergillus fumigatus***	QVWSVNGD-GKKMAEHAHCTVKLFNCAERETEWKRNSYLIKRSVSLLQDKA
**PKS-*Aspergillus clavatus***	QVYSVNAE-GKKMIEHANCTVKLFDCADREMEWKRISYLVKRSVSLLQDKA

**PKS1-*Metarhizium robertsii***	DQGLNSRMSSGVIYKLFSSLVDYSTA-YKGMQEAIVNTEDFEATALVRFQV
**PKS1-*Metarhizium anisopliae***	DQGLNSRMSSGVIYKLFSSLVDYSTA-YKGMQEAIVNTEDFEATALVRFQV
**PKS1-*Metarhizium brunneum***	DQGLNSRMSSGVIYKLFSSLVDYSTA-YKGMQEAIVNTEDFEATALVRFQV
**PKS1-*Metarhizium guizhouense***	DQGLNSRMSSGVIYKLFSSLVDYSTA-YKGMQEAIVNTEDFEATALVRFQV
**PKS1-*Metarhizium majus***	DQGLNSRMSSGVIYKLFSSLVDYSTA-YKGMQEAIVNTEDFEATALVRFQV
**PKS1-*Metarhizium acridum***	DQGLNSRMSSGVIYKLFSSLVDYSTA-YKGMQEAIVNTEDFEATALVRFQV
**PKS1-*Metarhizium album***	DQGLNSRLSSGVIYKLFSSLVEYSSA-YKGMKEAIVNTEDFEATALVQFQV
**PKS-*Trichoderma reesei***	DQGLNSRLSSGVIYKLFSSLVDYSQA-YKGMQEAIVDAEDFEATALVQFQV
**PKS-*Colletotrichum fioriniae***	TQGLDSTLATGMIYKVFSSLVDYQQDGFKGLREVVLHSEDLEGTAKVRFRG
**PKS-*Fusarium graminearum***	AQGLDSMLATGMVYKVFSSLVDYADG-YKGLQEVVLHSQELEGTAKVRFQT
**PKS2-*Metarhizium robertsii***	EQGTDSLLSRGIVYKLFSSLVDYSPS-FKGLQEVILNSGDREAAAKVRLQA
**PKS2-*Metarhizium anisopliae***	EQGTDSLLSKGIVYKLFSSLVDYSPS-FKGLQEVILNSGDREAAAKVRLQA
**PKS2-*Metarhizium brunneum***	EQGTDSLLSRGIVYKLFSSLVDYSPS-FKGLQEVILNSGDREAAAKVRLQA
**PKS2-*Metarhizium guizhouense***	EQGTDSLLSKGIVYKLFSSLVDYSPS-FKGLQEVILNSGDREATAKVRLQA
**PKS2-*Metarhizium majus***	EQGTDSLLSKGIVYKLFSSLVDYSPS-FKGLQEVILNSGDREATAKVRLQA
**PKS2-*Metarhizium acridum***	EQGTDSLLSKGIVYKLFSSLVDYSPS-FKGLQEVILNSSDREATAKVRLQA
**PKS-*Talaromyces marneffei***	ANGDSNKLGPGMVYKLFGALVDYDKN-YKSMREVILDSENYEATALVKFQA
**PKS-*Penicillium oxalicum***	ESGQAHRMKRGMVYKLFSTLVEYDEN-YKSIQEVILDSEQHEATALVKLQA
**PKS-*Aspergillus fumigatus***	QTGEAHRMQRGMVYKLFAALVDYDEN-FKAIQEVILDSNEHEATARVKFQA
**PKS-*Aspergillus clavatus***	QTGEAHRMQRGMVYKLFAALVDYDEN-FKSIQEVVLDSKQHEATAKVKFQA

**PKS1-*Metarhizium robertsii***	DEGNFRCNPMWVDSCGQLAGFLMNGHAKT-PKDQVFINHGWQYFRTVRKFS
**PKS1-*Metarhizium anisopliae***	DEGNFRCNPMWVDSCGQLAGFLMNGHAKT-PKDQVFINHGWQYFRTVRKFS
**PKS1-*Metarhizium brunneum***	DEGNFRCNPMWVDSCGQLAGFLMNGHAKT-PKDQVFINHGWQYFRTVRKFS
**PKS1-*Metarhizium guizhouense***	DEGNFRCNPMWVDSCGQLAGFLMNGHAKT-PKDQVFINHGWQSFRTVRKFS
**PKS1-*Metarhizium majus***	DEGNFRCNPMWVDSCGQLAGFLMNGHAKT-PKDQVFINHGWQSFRTVRKFS
**PKS1-*Metarhizium acridum***	DEGNFRCNPMWVDSCGQLAGFLMNGHAKT-PKDQVFINHGWQSFRTVRKLS
**PKS1-*Metarhizium album***	DEGNFRCNPMWVDSCGQLAGFLMNGHSKT-PLDQVFINHGWQSFRTVRKFC
**PKS-*Trichoderma reesei***	DEGSFHCNPMWIDSCGQLAGFLMNGHSKT-PKDQVFINHGWQSFRMVRKFR
**PKS-*Colletotrichum fioriniae***	PRGGFYCNPLWIDSCGQTTGFLMNCHQTT-PRDYVYVNHGWKSMKLAKDFQ
**PKS-*Fusarium graminearum***	PSGGFVCNPMWIDSCGQTTGFMMNCHQTT-PNDYVYVNHGWKSMRLAKAFR
**PKS2-*Metarhizium robertsii***	KKGDFDCNPMWIDSFGQLTGFLMNGHDFT-GKDEVFINHGWRSMRCAKPFR
**PKS2-*Metarhizium anisopliae***	EKGDFDCNPMWIDSFGQLTGFLMNGHDFT-GKDEVFINHGWRSMRCAKPFR
**PKS2-*Metarhizium brunneum***	EKGDFDCNPMWIDSFGQLTGFLMNGHDFT-GKDEVFINHGWRSMRCAKPFR
**PKS2-*Metarhizium guizhouense***	EKGDFGCNPMWIDSFGQLTGFLMNGHDFT-GKDEVFINHGWRSMRCAKPFR
**PKS2-*Metarhizium majus***	ERGDFDCNPMWIDSFGQLTGFLMNGHDFT-GKDEVFINHGWRSMRCAKPFR
**PKS2-*Metarhizium acridum***	EKEDFDYNPMWIDSFGQLTGFLMNGHDFT-EKDQVFINHGWRSMRCAKQFR
**PKS-*Talaromyces marneffei***	EAANFHRNPFWIDSIGHITGFTMNANDATDSANFVYVNHGWDSMRCATKFS
**PKS-*Penicillium oxalicum***	SPGNFHRNPFWIDSFGHLSGFIMNASDATDSKNTVYVNHGWESMRCLKKFD
**PKS-*Aspergillus fumigatus***	PPGNFHRNPFWIDSFGHLSGFIMNASDATDSKNQVFVNHGWDSMRCLKKFS
**PKS-*Aspergillus clavatus***	PAGNFHRNPFWIDSFGHLSGFIMNASDATDSKNQVFVNHGWDSMRCLKKFS

**PKS1-*Metarhizium robertsii***	RDKTYRTYVRMRCVEGTTYAGDVYIFD-DEGIV---------------
**PKS1-*Metarhizium anisopliae***	RDKTYRTYVRMRCVEGTTYAGDVYIFD-DEGIV---------------
**PKS1-*Metarhizium brunneum***	RDKTYRTYVRMRCVEGTTYAGDVYIFD-DDGIV---------------
**PKS1-*Metarhizium guizhouense***	KDKTYRTYVRMRCIEGTTYAGDVYIFD-DEGIV---------------
**PKS1-*Metarhizium majus***	KDKTYRTYVRMRCIEGTTYAGDVYIFD-DEGIV---------------
**PKS1-*Metarhizium acridum***	KDKTYRTYVRMRCIEGTTYAGDVYIFD-NEGIV---------------
**PKS1-*Metarhizium album***	KDKTYRTYVRMRCIEGTTYAGDVYIFD-DEGIV---------------
**PKS-*Trichoderma reesei***	KDQTYRTYVRMRPVEGTLYAGDVYIFD-DDGIV---------------
**PKS-*Colletotrichum fioriniae***	EGTTYRTFIHMRPVDGTKYAGDLYILDENSTVIGVYGDITFQGLPRRV
**PKS-*Fusarium graminearum***	EDGTYRTYIRMRPIDSTKFAGDLYILDEDDTVVGVYGDITFQGLPRRV
**PKS2-*Metarhizium robertsii***	KDAVYRTYIRMQHVEKTKYRGDLYIIE-DGVIVAVFGGMTF-------
**PKS2-*Metarhizium anisopliae***	KDAVYRTYIRMQHVEKTKYRGDLYIIE-DGVIVAVFGGMTF-------
**PKS2-*Metarhizium brunneum***	KDAVYRTYIRMQHVEKTKYRGDLYIIE-DGVIIAVFGGMTF-------
**PKS2-*Metarhizium guizhouense***	KDAVYRTYIRMQHVEKTKYRGDLYIIE-DGVIIGVFGGMTF-------
**PKS2-*Metarhizium majus***	KDAVYRTYIRMQHVEKTKYRGDLYIIE-DGVVIAVFGGMTFL------
**PKS2-*Metarhizium acridum***	KDAVYRTYICMQNVDKTKYCGDLYIIE-DGVIIAVFGGMTVLP-----
**PKS-*Talaromyces marneffei***	ADATYRTYVRMQQWQGTIYSGDVYVFE-KDEIIAVYGGVKFQGVPRKI
**PKS-*Penicillium oxalicum***	PNVTYRTYVRMQPWQNAIFAGDVYVFD-GDDIVAVYGGVQFQALSRKI
**PKS-*Aspergillus fumigatus***	GDATYQTYVKMQPWKDSIWAGDVYVFE-GDDIIAVYGGVKFQALARKI
**PKS-*Aspergillus clavatus***	ADATYQTYVKMQPWRDSIWAGDVYVFE-GDDIIAVYGGVKFQALARKI

Alignment of TE domain

**PKS1-*Metarhizium robertsii***	TLFLLPDGSGSSFSYAPI-NAVRK--DVCVFGLNCPWLKSAEKLVQFG--
**PKS1-*Metarhizium anisopliae***	TLFLLPDGSGSSFSYAPI-NAVRK--DVCVFGLNCPWLKSAEKLVQFG--
**PKS1-*Metarhizium brunneum***	TLFLLPDGSGSSFSYAPI-NAVRK--DVCVFGLNCPWLKSAEKLVQFG--
**PKS1-*Metarhizium guizhouense***	TLFLLPDGSGSSFSYAPI-NAVRK--DVCVFGLNCPWLKSAEKLVQFG--
**PKS1-*Metarhizium majus***	TLFLLPDGSGSSFSYAPI-NAVRK--DVCVFGLNCPWLKSAEKLVQFG--
**PKS1-*Metarhizium acridum***	TLFLLPDGSGSSFSYAPI-NAVRK--DVCVYGLNCPWLKSAEKLVQFG--
**PKS1-*Metarhizium album***	TLFLLPDGSGSATSYATI-SAVGR--DICVYGLNCPWLKSAEKLVQFG--
**PKS-*Trichoderma reesei***	TLFLLPDGSGSATSYASL-PPISPNGDIAVYGLNCPWLKDAQHLVEFG--
**PKS-*Colletotrichum fioriniae***	TLFLLPDGSGSATSYAPI-NAIGK--EVCVYGLTCPWLKSADKLVQFG--
**PKS-*Fusarium graminearum***	TLFLLPDGSGSATSYASL-PPISPDGDVAVYGLNCPWLKDASYLVEFG--
**PKS2-*Metarhizium robertsii***	ILVLFPDGSGSAASYGALAPKIRR--DIAVYALNCPWRTNGEEILRLGVT
**PKS2-*Metarhizium anisopliae***	ILVLFPDGSGSAASYGALAPKIRR--DIAVYALNCPWRTNGEEILRLGVT
**PKS2-*Metarhizium brunneum***	ILVLFPDGSGSAASYGALAPKIRR--DIAVYALNCPWRTNGEEILRLGVT
**PKS2-*Metarhizium guizhouense***	ILVLFPDGSGSAASYGALAPKIRR--DIAVYALNCPWRTNGEEILRLGVS
**PKS2-*Metarhizium majus***	ILVLFPDGSGSAASYGALAPKIRR--DTAVYALNCPWRTNGEEILRLGVS
**PKS2-*Metarhizium acridum***	ILVLFPDGSGSAASYGALAPKIRK--DIAVYALNCPWRTNGEEILRLGVS
**PKS-*Talaromyces marneffei***	TLFLFPDGSGSSTSYATI-PKISP--DVCVYGLNCPYMKTPEKL-NCA--
**PKS-*Penicillium oxalicum***	KLFMFPDGSGSASSYATI-PALSP--DVCVYGLNCPYMKTPQNL-TCS--
**PKS-*Aspergillus fumigatus***	KLFLFPDGSGSASSYATI-PALSP--DICVYGLNCPYMKTPQNL-KCS--
**PKS-*Aspergillus clavatus***	KLFLFPDGSGSATSYATI-PGISP--DVCVYGLNCPYMRTPEKL-NFP--

**PKS1-*Metarhizium robertsii***	LKGLATLYVEEIRR---RAPHGP---------YNLGGWSAGGICAYEAAI
**PKS1-*Metarhizium anisopliae***	LKGLATLYVEEIRR---RAPHGP---------YNLGGWSAGGICAYEAAI
**PKS1-*Metarhizium brunneum***	LKGLATLYVEEIRR---RAPHGP---------YNLGGWSAGGICAYEAAI
**PKS1-*Metarhizium guizhouense***	LKGLATLYVEEIRR---RAPHGP---------YNLGGWSAGGICAYEAAI
**PKS1-*Metarhizium majus***	LKGLATLYVEEIRR---RAPHGP---------YNLGGWSAGGICAYEAAI
**PKS1-*Metarhizium acridum***	LKGLATLYVEEIRR---RAPHGP---------YNLGGWSAGGICAYEAAI
**PKS1-*Metarhizium album***	LKGLASLYVGEIRR---RAPHGP---------YNLGGWSAGGICAYEAAI
**PKS-*Trichoderma reesei***	LRGLAELYVSEILR---RQPRGP---------YDLGGWSAGGICAYEVSL
**PKS-*Colletotrichum fioriniae***	LKGLAALYVEEIRR---RVPHGP---------YNLGGWSAGGICAYEAAI
**PKS-*Fusarium graminearum***	LKGLTELYVNEILR---RKPQGP---------YNLGGWSAGGICAYEAAL
**PKS2-*Metarhizium robertsii***	LDQMVAKHLVEVGRILDSHRHGRPGSANASVGLALGGWSAGGILALEAVR
**PKS2-*Metarhizium anisopliae***	LDQMVAKHLVEVGRILDSHQRGRPGSSNASVGLALGGWSAGGILALEAVR
**PKS2-*Metarhizium brunneum***	LDTMVAKHLVEVGRILDSHRHGRPGSGNSSVGLALGGWSAGGILALEAVR
**PKS2-*Metarhizium guizhouense***	LDQMVAKHLVEVGRILDRHQHCRAGSGNASVGLALGGWSAGGILALEAVR
**PKS2-*Metarhizium majus***	LDQMVAKHLVEVGRILDSHQHGRPGSGNASVGLALGGWSAGGILALEAVR
**PKS2-*Metarhizium acridum***	LDQMVAKHLIEVRRILDNHQQSRL---NGSIDLALGGWSAGGILAIEGVR
**PKS-*Talaromyces marneffei***	LQDLTASYVTEIRR---RQPKGP---------YNVGGWSAGGICAYDAVR
**PKS-*Penicillium oxalicum***	LDELTEPYLAEIRR---RQPKGP---------YSFGGWSAGGICAFDAAR
**PKS-*Aspergillus fumigatus***	LEELTEPYLAEIRR---RQPTGP---------YNFGGWSAGGICAFDAAR
**PKS-*Aspergillus clavatus***	LQELTFPYVAEIRR---RQPTGP---------YNFGGWSAGGICAYDAAR

**PKS1-*Metarhizium robertsii***	QFT-REGETVERLILLDSPNP-------------------------
**PKS1-*Metarhizium anisopliae***	QFT-REGETVERLILLDSPNP-------------------------
**PKS1-*Metarhizium brunneum***	QFT-REGETVERLILLDSPNP-------------------------
**PKS1-*Metarhizium guizhouense***	QFT-REGETVERLILLDSPNPI------------------------
**PKS1-*Metarhizium majus***	QFT-REGETVERLILLDSPNPI------------------------
**PKS1-*Metarhizium acridum***	QFT-REGETVERLILLDSPNPI------------------------
**PKS1-*Metarhizium album***	QFT-REGEAVERLILLDSPNP-------------------------
**PKS-*Trichoderma reesei***	RLT-RDGEKVERLILLDSPNP-------------------------
**PKS-*Colletotrichum fioriniae***	MLT-RAGHRVDRLVLIDSPSP-------------------------
**PKS-*Fusarium graminearum***	ILT-RAGHQVDRLILIDSPNPVGL----------------------
**PKS2-*Metarhizium robertsii***	QLG-EAGVAVQKMVLLDAPNPI------------------------
**PKS2-*Metarhizium anisopliae***	QLR-EAGVAVQKMVLLDAPNPI------------------------
**PKS2-*Metarhizium brunneum***	QLG-EAGVAVQKMVLLDAPNPI------------------------
**PKS2-*Metarhizium guizhouense***	QLR-EAGVAVQKMVLLDAPNPI------------------------
**PKS2-*Metarhizium majus***	QLR-EAGVAVQKMVLLDAPNPI------------------------
**PKS2-*Metarhizium acridum***	QLR-QTGIVVQKLVLLDAPNPI------------------------
**PKS-*Talaromyces marneffei***	QLVVEQGETVERIFFLDSPFP-------------------------
**PKS-*Penicillium oxalicum***	HLIFEEGERVERLLLLDSPFPIG-----------------------
**PKS-*Aspergillus fumigatus***	QLILEEGEEVERLLLLDSPFPIGLEKLPPRLYKFFNSIGLFGDGKR
**PKS-*Aspergillus clavatus***	QLILEEGEVVERLLLLDSPFPIG-----------------------
