# Supplementary material for: Duplication of a Pks gene cluster and subsequent functional diversification facilitate environmental adaptation in Metarhizium species
Source: PLoS Genet. 2018 Jun 29;14(6):e1007472. doi: 10.1371/journal.pgen.1007472 (PMC6042797; doi:10.1371/journal.pgen.1007472)
Supplement: S5 Dataset — (PDF) [file pgen.1007472.s033.pdf]

Supplementary dataset 5

Dataset for Figure 4 [include part 1 (motif logos and sequences in each gene) and part 2 (the promoter sequences of *Pks1* and *Pks2* genes)**]**

<u>Part 1: Motif logos and motif sequences in each gene</u>

Motif 1

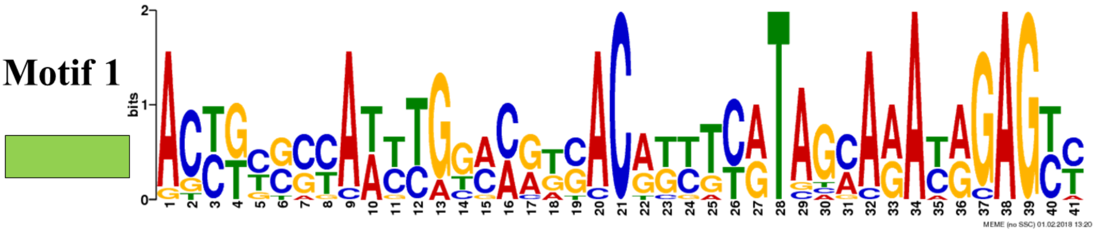

Gene Name	Sequences
**PKS1 *Metarhizium anisopliae***	**ACTTCGATAATTGTAAAGCACATTTCGTAGCAAATAGAGTT**
**PKS1 *Metarhizium brunneum***	**ACTTTGGTAATTGTAAAGCACATTTCGTAGCAAATAGAGTT**
**PKS1 *Metarhizium robertsii***	**ACTTTGGCAATTGTAAAGCACATTTCGTAGCAAATAGAGTT**
**PKS1 *Metarhizium guizhouense***	**ACTTTGCTAATTGCACAACACATCTCGTAGCAAATAGAGTT**
**PKS1 *Metarhizium majus***	**ACTTTGGTAATTGCAACACACATCTCGTAGCAAATAGAGTT**
**PKS1 *Metarhizium acridum***	**AGCGGGCCCATCAGGCGGCCCTGCTCATCAGCAATAGAGCA**
**PKS2 *Metarhizium acridum***	**GTTGGTGGATGCAGAAGTTACATGTCATGTCAAAAACAGTC**
**PKS2 *Metarhizium guizhouense***	**ACCGCCCCATCTGGCCGTGACGGTGTATAGAAGACGGAGCC**
**PKS2 *Metarhizium majus***	**ACCGCCCCATCTGGCCGTGACGGTGTATAGAAGACGGAGCC**
**PKS2 *Metarhizium brunneum***	**AGCGCCCCATCCGGCCGTGACGGTGTATACCAGACGGAGCC**
**PKS2 *Metarhizium robertsii***	**ACCGCCCCATCCGGCCGTGACAGCATATAGAAGACGGAGCC**
**PKS2 *Metarhizium anisopliae***	**ACCGCCCCATCTGGCCGTGACGCCTCATAGAAGACGGAGCC**

Motif 2

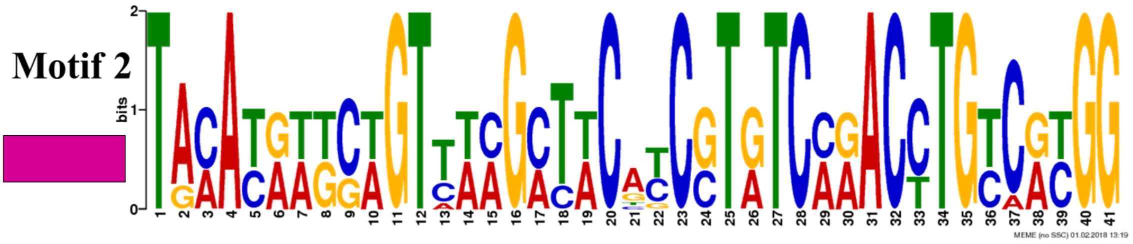

Gene Name	Sequences
PKS1 *Metarhizium anisopliae*	TAAACGTGCTGTAAAGATACGTCCTGTCCAACCTGCCACGG
PKS1 *Metarhizium brunneum*	TAAACGTGGTGTCAAGATACGTCCTATCCAACCTGCCACGG
PKS1 *Metarhizium robertsii*	TGAACGTGGTGTCAAGATACCTCCTATCCAACTTGCCACGG
PKS1 *Metarhizium guizhouense*	TAAACGTGGTGTCAAGATACTTCCTATCCAACTTGCCACGG
PKS2 *Metarhizium acridum*	TGCATGTGCTGTTAAGCTACATCGTATCCAACTTGTAACGG
PKS2 *Metarhizium guizhouense*	TACATAATCAGTTTCGCTTCACCGTGTCAGACCTGTCGTGG
PKS2 *Metarhizium majus*	TACATAATCAGTTTCGCTTCAGCGTGTCAGACCTGTCGTGG
PKS2 *Metarhizium brunneum*	TACATAATCAGTTTCGCCTCACCGTGTCAGACCTGTCGTGG
PKS2 *Metarhizium robertsii*	TACATAATCAGTTTCGCCTCACCGTGTCAGACCTGTCGTGG
PKS2 *Metarhizium anisopliae*	TACATAATCAGTTTCGCTTCACCGTGTCAGACCTGTCGTGG

Motif 3

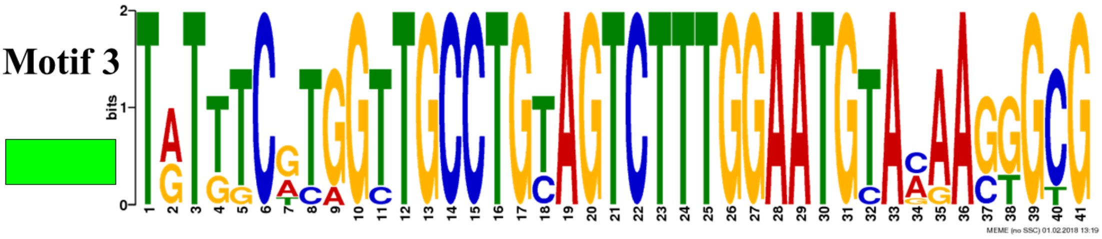

Gene Name	Sequences
PKS1 *Metarhizium anisopliae*	TGTTTCGTGGTTGCCTGTAGTCTTTGGAATGTACAAGGGCG
PKS1 *Metarhizium brunneum*	TGTTTCGTGGTTGCCTGTAGTCTTTGGAATGTACAAGGGCG
PKS1 *Metarhizium robertsii*	TGTTGCGTGGTTGCCTGTAGTCTTTGGAATGTACAAGGGCG
PKS1 *Metarhizium guizhouense*	TATTTCATGGTTGCCTGTAGTCTTTGGAATGTAAAAGGGCG
PKS1 *Metarhizium majus*	TATTTCATGGTTGCCTGTAGTCTTTGGAATGTAAAAGGGCG
PKS1 *Metarhizium album*	TATGTCTCAGTTGCCTGCAGTCTTTGGAATGCAAAACTGTG
PKS2 *Metarhizium acridum*	TATGTCGTGGCTGCCTGCAGTCTTTGGAATGTAGGACTGCG

Motif 4

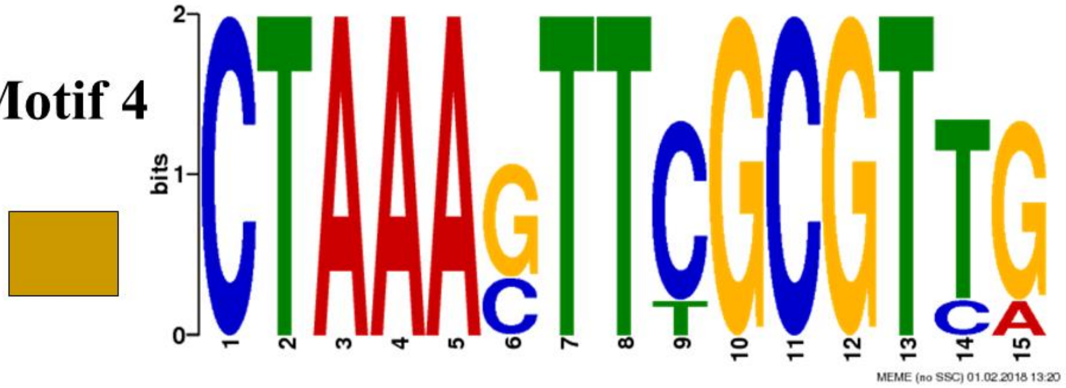

Gene Name	Sequences
PKS1 *Metarhizium anisopliae*	CTAAAGTTCGCGTTG
PKS1 *Metarhizium brunneum*	CTAAAGTTCGCGTTG
PKS1 *Metarhizium robertsii*	CTAAAGTTCGCGTTG
PKS1 *Metarhizium guizhouense*	CTAAACTTCGCGTTG
PKS1 *Metarhizium majus*	CTAAAGTTCGCGTTG
PKS2 *Metarhizium acridum*	CTAAACTTTGCGTCA

Motif 5

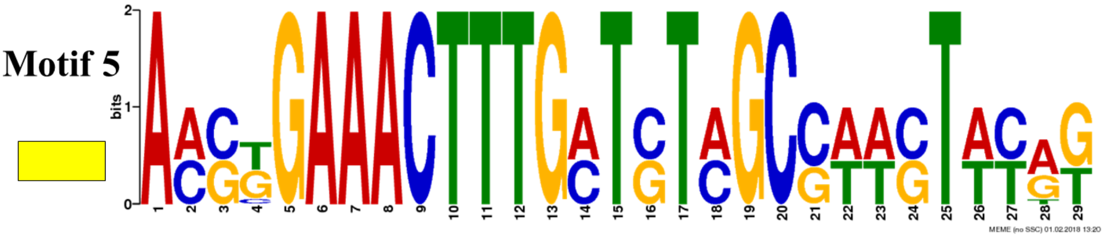

Gene Name	Sequences
PKS1 *Metarhizium anisopliae*	AAGGGAAACTTTGATCTAGCCAACTACAG
PKS1 *Metarhizium brunneum*	AAGGGAAACTTTGATCTAGCCAACTACAG
PKS1 *Metarhizium robertsii*	AAGGGAAACTTTGATCTAGCCAACTACAG
PKS1 *Metarhizium guizhouense*	AAGGGAAACTTTGATCTAGCCAACTACAG
PKS1 *Metarhizium majus*	AAGGGAAACTTTGATCTAGCCAACTACAG
PKS2 *Metarhizium acridum*	AACCGAAACTTTGATCTAGCCAACTACAG
PKS2 *Metarhizium guizhouense*	ACCTGAAACTTTGCTGTCGCGTTGTTTGT
PKS2 *Metarhizium majus*	ACCTGAAACTTTGCTGTCGCCTTGTTTGT
PKS2 *Metarhizium brunneum*	ACCTGAAACTTTGCTGTCGCGTTGTTTGT
PKS2 *Metarhizium robertsii*	ACCTGAAACTTTGCTGTCGCGTTGTTTGT
PKS2 *Metarhizium anisopliae*	ACCTGAAACTTTGCTGTCGCGTTGTTTTG

Motif 6

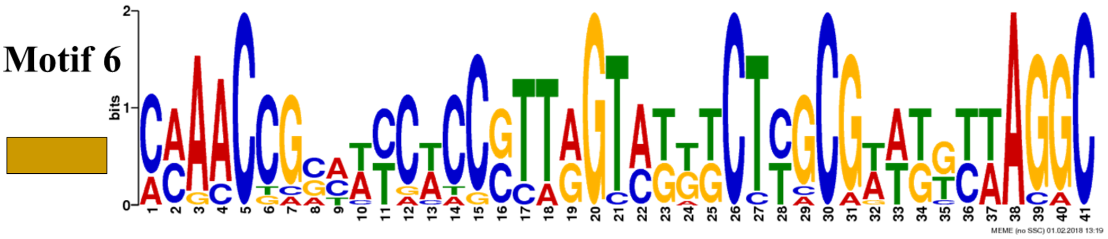

Gene Name	Sequences
PKS1 *Metarhizium anisopliae*	CAAACCGGTATCACCCTTAGTATGTCTCGCGAATGTTAGGC
PKS1 *Metarhizium brunneum*	CAAACCGGAATCACCCTTAGTATGTCTTGCGAATTTTAGGC
PKS1 *Metarhizium robertsii*	CAAACCGGAATCACCCTTAGCATGTCTTGCGAATTTTAGGC
PKS1 *Metarhizium guizhouense*	AAAACCGAAATCACCCCTAGTATGTCTTGCGAATTTTAGGC
PKS1 *Metarhizium majus*	AAACCGAAATCACCCCCTAGTATGTCTTGCGAATTTTACGC
PKS2 *Metarhizium acridum*	AAAACCGGAATCAACGTTAGTATATCTTGCAGATCTTAGAC
PKS2 *Metarhizium guizhouense*	CCAACCGCCTCCTCCGTTGGTCGTGCCCACGTTGGCAAGGC
PKS2 *Metarhizium majus*	CCAACCGCCTCCTCCGTTGGTCGTGCTCGCGTTGGCAAGGC
PKS2 *Metarhizium brunneum*	CCGCCTCCTCCGTTGGTTGGTAGTGCTCCCGTTGGCAAGGC
PKS2 *Metarhizium robertsii*	CCAACCGCCTCCTCCGTAGGTCGTGCTCGCGTTGGCAAGGC
PKS2 *Metarhizium anisopliae*	CCAACCGCCTCCTCCGTAGGTCGTGCTCGCGTTGGCAAGGC

Motif 7

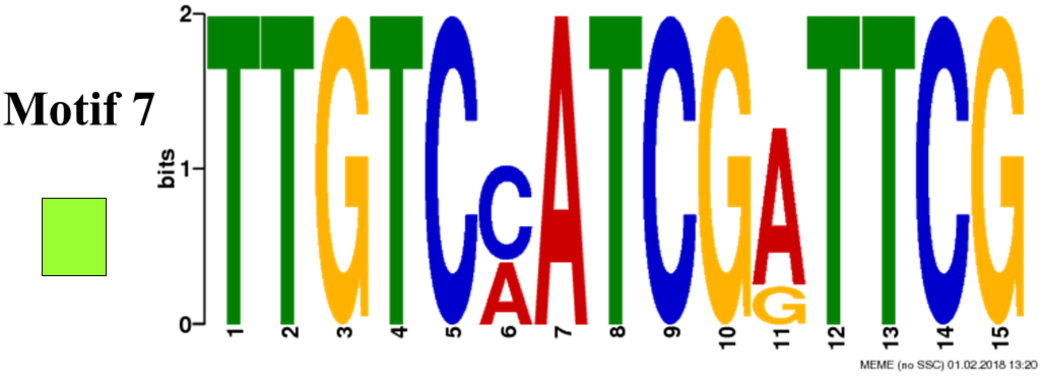

Gene Name	Sequences
PKS1 *Metarhizium anisopliae*	TTGTCCATCGGTTCG
PKS1 *Metarhizium brunneum*	TTGTCCATCGATTCG
PKS1 *Metarhizium robertsii*	TTGTCCATCGATTCG
PKS1 *Metarhizium guizhouense*	TTGTCAATCGATTCG
PKS1 *Metarhizium majus*	TTGTCAATCGATTCG

Motif 8

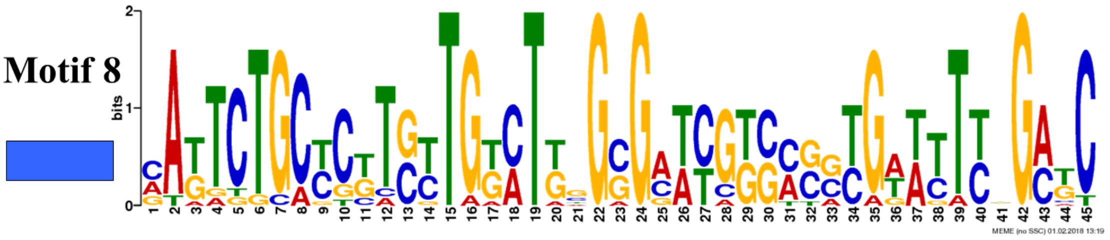

Gene Name	Sequences
PKS1 *Metarhizium anisopliae*	GATTCTGCCCTTGTTATCTTGGCGATCGTGCCGTGAACTTGGATC
PKS1 *Metarhizium brunneum*	GATTCTGCCCTTGTTGTCTTGGCGATCGTGCCGTGAACTTGGATC
PKS1 *Metarhizium robertsii*	GATTCTGCCCTTGTTGTCTTGGCGATCGTGCCGTGATCTTGGATC
PKS1 *Metarhizium guizhouense*	AATTCTGCCCTTGTTGTCTTAGCGATCGTGCCGTGAATTTCGATC
PKS1 *Metarhizium majus*	AATTCTGCCCTTGTTGTCTTGGCGATCGTGCCGTGAATTTCGATC
PKS1 *Metarhizium acridum*	AAAACTGATGCAGTTGGCTTGGAGGTCCGCTGATAGTGACTGAAT
PKS1 *Metarhizium album*	CATGGTGCGGCTCCTGACTAGGCGGTCATCCCATGGACTTGGACC
PKS2 *Metarhizium acridum*	ATTTTGCACTTTGTTGTCTGAGCGATCCTGCTGTGAATTTCGATC
PKS2 *Metarhizium guizhouense*	CAGTCTGCTCGTCCTGGATGCGGGCATGGCAGCCGTTTTCTGCGC
PKS2 *Metarhizium majus*	CAGTCTGCTCGCCCTGGATGCGGGAATGGCAGCCGTTTTCTGCGC
PKS2 *Metarhizium brunneum*	CAGTCTGCTCGTCGTGGATGCGGGCATGGCAGCCGTTTTCTGCGC
PKS2 *Metarhizium robertsii*	CAGTCTGCTCGTCCTGGATGTGGGCATGGCAGCCGTTTTCAGCGC
PKS2 *Metarhizium anisopliae*	CAGTCTGCTCGTCCTGGATGTGGGCATGGCAGCCGTTTTCAGCGC

Motif 9

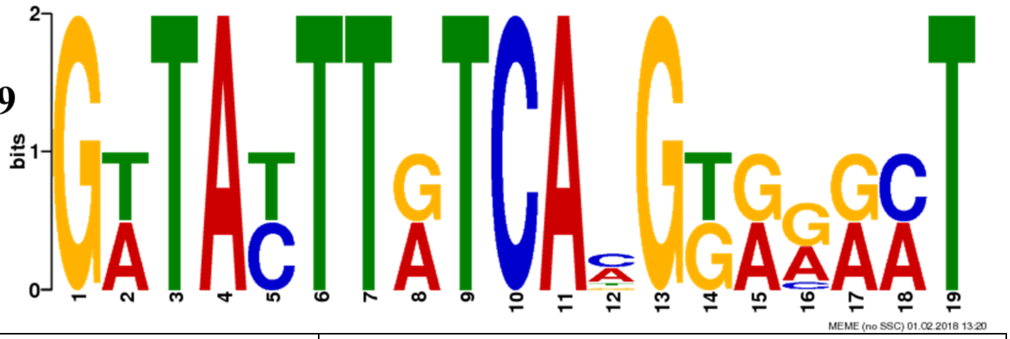

Gene Name	Sequences
PKS1 *Metarhizium anisopliae*	GTTACTTGTCACGTAGAAT
PKS1 *Metarhizium brunneum*	GTTACTTGTCACGTAGAAT
PKS1 *Metarhizium robertsii*	GTTACTTGTCACGTAGAAT
PKS1 *Metarhizium guizhouense*	GTTACTTGTCATGTAGAAT
PKS1 *Metarhizium majus*	GTTACTTGTCACGTAGAAT
PKS2 *Metarhizium guizhouense*	GATATTTATCAAGGGAGCT
PKS2 *Metarhizium majus*	GATATTTATCAGGGGCGCT
PKS2 *Metarhizium brunneum*	GATATTTATCAAGGGAGCT
PKS2 *Metarhizium robertsii*	GATATTTATCAAGGGAGCT
PKS2 *Metarhizium anisopliae*	GATATTTATCAAGGGAGCT

Motif 10

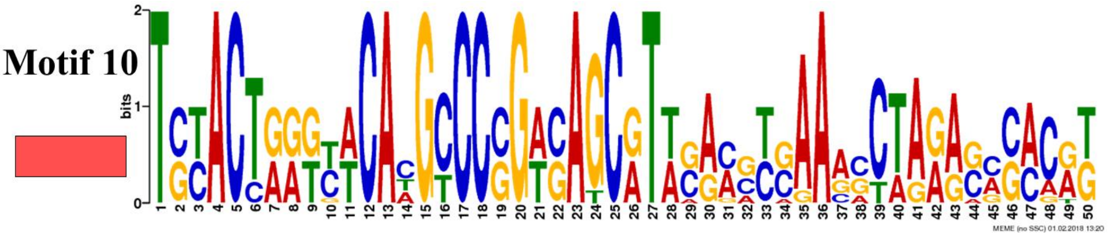

Gene Name	Sequences
PKS1 *Metarhizium anisopliae*	TCTACTAAGCACAAGCCCCGACAGCGTTGACGCCAAACCTAGACCGACGT
PKS1 *Metarhizium brunneum*	TCTACTAAGCACAAGCCCCGACAGCGTTGACGCCAAACCTAGACCGACGT
PKS1 *Metarhizium robertsii*	TCTACTAAGCACAAGCCCCGACAGCGTTGACGCCAAACCTAGACCGACGT
PKS1 *Metarhizium guizhouense*	TCTACTAAGCACATGCCCCGACAGCGTTGACGCCAAACCTAGACCGACGT
PKS1 *Metarhizium majus*	TCTACCAAGCACATGCCCCGACAGCGTTGACGCCAAACCTAGACCGACGT
PKS1 *Metarhizium album*	TCTACCGGGGACACGTCCCGACATCGTTAAGCTAAAAGCTAGAAACCGTT
PKS2 *Metarhizium guizhouense*	TGCACTGGTTTCACGCCCGGTGAGCATACAACTGAAGGCAAAGGACCCAG
PKS2 *Metarhizium majus*	TGCACTGGTTTCATGCCCGGTGAGCATACAACTGGAGGCAAAGGACCCAG
PKS2 *Metarhizium brunneum*	TGCACTGGTTTCACGCCCGGTGAGCATACGACTGAAGGCAAAGGACCCAG
PKS2 *Metarhizium robertsii*	TGCACTGGTTTCACGTCCGGTGAGCATACGAATGAACATTGAAGGCAAAG
PKS2 *Metarhizium anisopliae*	TGCACTGGTTTCACGTCCGGTGAGCATACGAATGAACATTGAAGGCAAAG

Motif 11

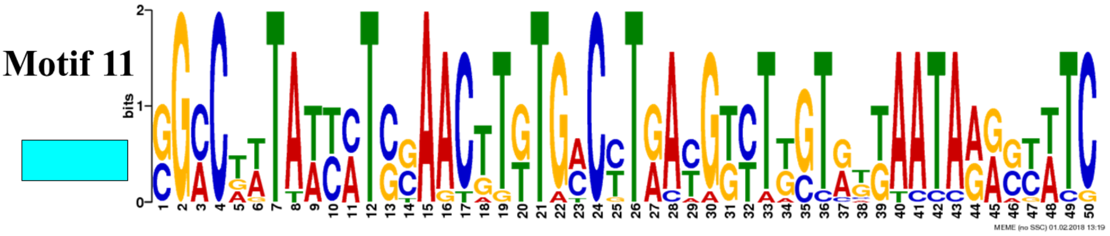

Gene Name	Sequences
PKS1 *Metarhizium anisopliae*	GGCCGTTATTCTCGAACTTGTGACCTAACGTCTTGTAGTAATAAAGTTTC
PKS1 *Metarhizium brunneum*	GGCCGTTATTCTCGAACTTGTGACCTAACGTCTTGTAGTAATAAAGTTTC
PKS1 *Metarhizium robertsii*	GGCCGTTATTCTCGAACTTGTGACCTAACGTCTTGTAGTAATAAAGTTTC
PKS1 *Metarhizium guizhouense*	GGCCATTATTCTCGAACGTGTGACCTAACGTCTTCTAGTAATAAAGTTTC
PKS1 *Metarhizium majus*	GGCCATTATTCTCGAACGTGTGACCTAACGTCTTCCAGTAATAAAGTTTC
PKS1 *Metarhizium acridum*	GGCCTGTTTCATCTAGTAGGTGTCGTGAAATCAAGTCCTTCCCAGAGACG
PKS2 *Metarhizium acridum*	GGCCATTATTCTCGAACGTGTAACCTGCCGTCTTCTGATAATAAAGTTTC
PKS2 *Metarhizium guizhouense*	CGACTATAACATGCAACTTTTGCCTTGATGGTTGGTGTGAATAGGCCATC
PKS2 *Metarhizium majus*	CGACTATAACATGCAACTTTTGCCTTGATGGTTGGTGTGAATAGGCCATC
PKS2 *Metarhizium brunneum*	CGACTATAACATGCAACTTTTGCCTTGATGGTTGGTGTGAATAGGCCATC
PKS2 *Metarhizium robertsii*	CGACTATAACATGCAACTTTTGCCTTGATGGTTGGTGTGAATAGGCCATC
PKS2 *Metarhizium anisopliae*	CGACTATAACATGCAACTTTTGCCTTGATGGTTGGTGTGAATAGGCCATC

Motif 12

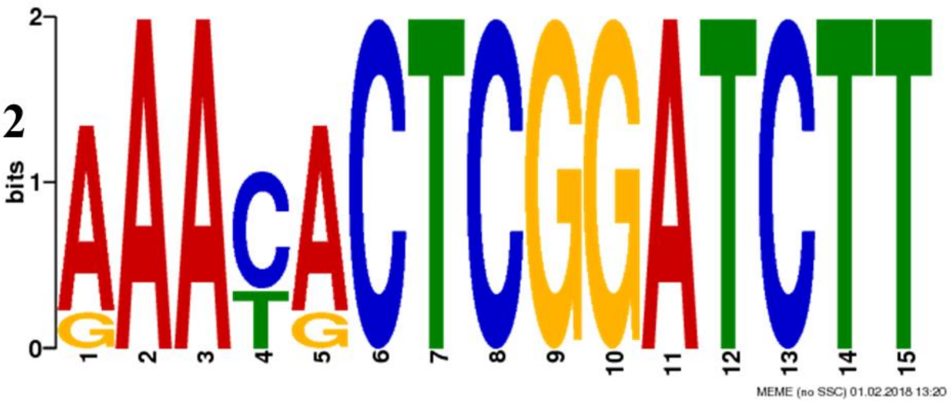

Gene Name	Sequences
PKS1 *Metarhizium anisopliae*	AAACACTCGGATCTT
PKS1 *Metarhizium brunneum*	AAACACTCGGATCTT
PKS1 *Metarhizium robertsii*	AAACACTCGGATCTT
PKS1 *Metarhizium guizhouense*	AAATACTCGGATCTT
PKS1 *Metarhizium majus*	AAATACTCGGATCTT
PKS2 *Metarhizium acridum*	GAACGCTCGGATCTT

Motif 13

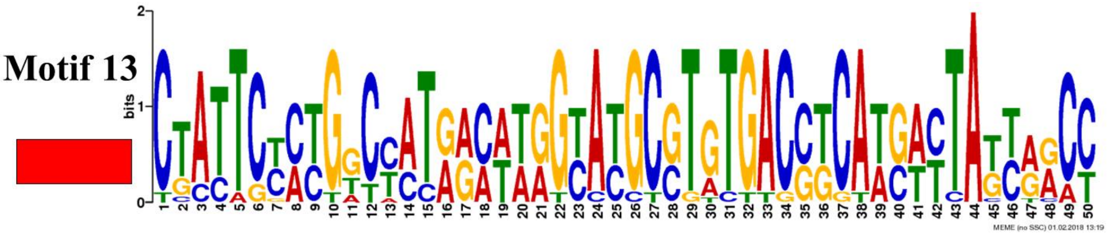

Gene Name	Sequences
PKS1 *Metarhizium anisopliae*	CTATTCCCTGGCCATGACATGGTATGCGTGTGACCTCATGATTATTAACC
PKS1 *Metarhizium brunneum*	CTATTCCCTGGCCATGACATGGTATGCGTGTGACCTCATGATTATTAACC
PKS1 *Metarhizium robertsii*	CTATTCCCTGGCCATGACATGGTATGCGTGTGACCTCATGATTATTAACT
PKS1 *Metarhizium guizhouense*	CTATTCCCTGGCCCTGAAATGGTATGCGTGTGACCTCATGATTATTAACT
PKS1 *Metarhizium majus*	CTATTCCCTGGCCCTGAAATGGTATGCGTGTGACCTCATGATTATTAACT
PKS1 *Metarhizium acridum*	TCACAGTCTGTTAACGAATTGTTCTCCGTTCTTGCTGTTGACCACCACAC
PKS1 *Metarhizium album*	CTACTGGCTTGTCCCAAAATGGCATGCGGGTGACCTCATGACTATCTGAC
PKS2 *Metarhizium acridum*	CTACTCCCTGGCCCTGGAATGGTATGTGTGTGACCTCATGATTATTAACT
PKS2 *Metarhizium guizhouense*	CGATTCTACGTCTATAGCTAAGCACGCCTGTGACGGCAACTCTAGCGGCC
PKS2 *Metarhizium majus*	CGATTCTACGTCTATAGCTAAGCACGCCTGTGACGGCAACTCTAGCGGCC
PKS2 *Metarhizium brunneum*	CTCTTCTACGACTATAGCTAAGCACGCCTATGACGGCAACTCTAGCGGCC
PKS2 *Metarhizium robertsii*	CTCTTCTACGACTATAGCTAAGCACGCCTATGACGGCAACTCTAGCGGCC
PKS2 *Metarhizium anisopliae*	CGATTCTACGTCTATAGCTAAGCACGCCTATGACGGCAACTCTAGTGGCC

Motif 14

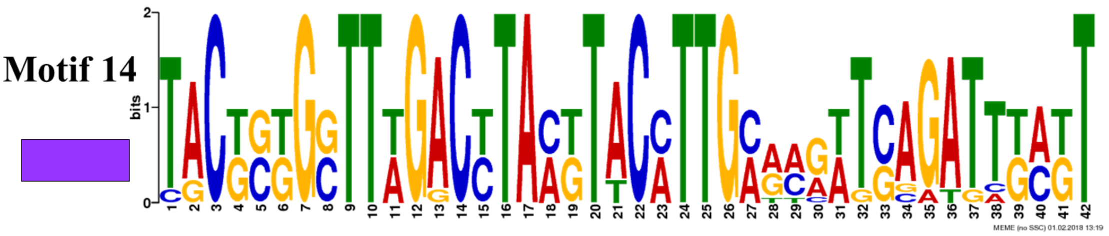

Gene Name	Sequences
PKS1 *Metarhizium anisopliae*	TACTCGGGTTTGACTTAAGTACATTGAGCAATGAGATTGCTT
PKS1 *Metarhizium brunneum*	TACTCGGGTTTGGCTTAAGTACATTGAGCAATGAGATTGCTT
PKS1 *Metarhizium robertsii*	TACTCGGGTTTGACTTAAGTACATTGAGCAATGAGATTGCTT
PKS1 *Metarhizium guizhouense*	TGCTCGGGTTTGACTTAAGTACATTGAGCCATGAGATTGCTT
PKS1 *Metarhizium majus*	TGCTCGGGTTTGACTTAAGTACATTGATTGAGCCATGAGATT
PKS2 *Metarhizium guizhouense*	TACGGTGCTTAGACCTACTTACCTTGCAAGTTCAGATTTAGT
PKS2 *Metarhizium majus*	CACGGTGCTTAGACCTACTTACCTTGCAAGTTCAGATTTAGT
PKS2 *Metarhizium brunneum*	TACGGTGCTTAGACCTACTTACCTTGCAAGTTCGGATCTAGT
PKS2 *Metarhizium robertsii*	TACGGTGCTTAGACCTACTTTCCTTGCAAGTTCAGATTTAGT
PKS2 *Metarhizium anisopliae*	TACGGTGCTTAGACCTACTTTCCTTGCAAGTTCAGATTTAGT

Motif 15

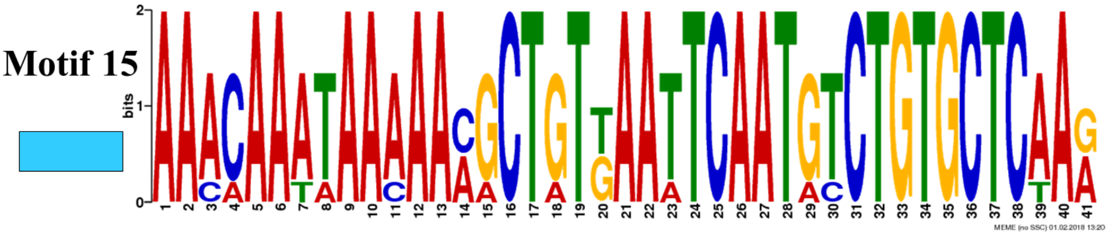

Gene Name	Sequences
PKS1 *Metarhizium anisopliae*	AAACAAATAAAAACGCTGTGAATTCAATGTCTGTGCTCAAG
PKS1 *Metarhizium brunneum*	AAACAAATAAAAACGCTGTGAATTCAATGTCTGTGCTCAAG
PKS1 *Metarhizium robertsii*	AAACAAATAAAAACGCTGTGAATTCAATGTCTGTGCTCAAG
PKS1 *Metarhizium guizhouense*	AAACAAATAAAAAAGCTGTTAATTCAATGTCTGTGCTCAAA
PKS1 *Metarhizium majus*	AACAAATAAAAAAAGCTGTTAATTCAATGCCTGTGCTCAAA
PKS2 *Metarhizium acridum*	AAACAAATAACAAAACTATTAAATCAATATCTGTGCTCTAA

Motif 16

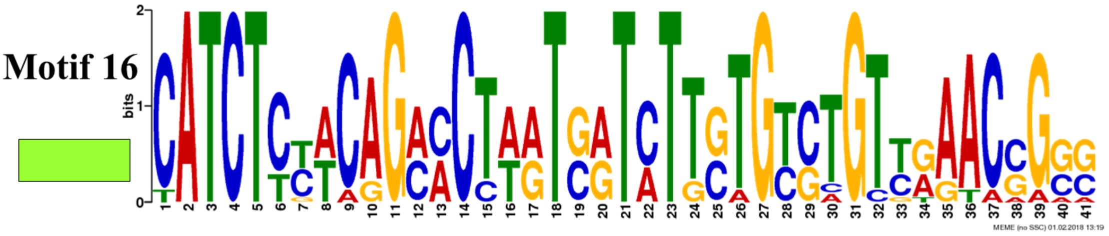

Gene Name	Sequences
PKS1 *Metarhizium anisopliae*	CATCTCTACAGACCTAATGATCTTGTGTCTGTTGAACCGGG
PKS1 *Metarhizium brunneum*	CATCTCTACAGACCTAATGATCTTGTGTCTGTTGAACCGGG
PKS1 *Metarhizium robertsii*	CATCTCTACAGACCTAATGATCTTGTGTCTGTTGAACCGGG
PKS1 *Metarhizium guizhouense*	CATCTTTACAGACCTAATGATCTTGTGTCTGTTGAACCGGG
PKS1 *Metarhizium majus*	CATCTTTACGGACCCAATGATCTTGTGTCTGTTGAACCGGG
PKS2 *Metarhizium acridum*	TATCTTCAAGGACCTAATGATCTTGAGTCCGTTGGACCGGA
PKS2 *Metarhizium guizhouense*	CATCTCCTCAGCACTTGTCGTATGCTGCGTGTCAAAAGGCC
PKS2 *Metarhizium majus*	CATCTCCTCAGCACCTGTCGTCTGCTGCCAGCGTGTCAAAG
PKS2 *Metarhizium brunneum*	CATCTCGTCAGCACTTGTCGTATTCTGCGTGTCAAACGGCC
PKS2 *Metarhizium robertsii*	CATCTCCTCAGCACTTGTCGTATTCTGTGTGTCAAACGGCC
PKS2 *Metarhizium anisopliae*	CATCTCCTCAGCACTTGTCGTATTCTGCGTGTCAAACGGCC

Motif 17

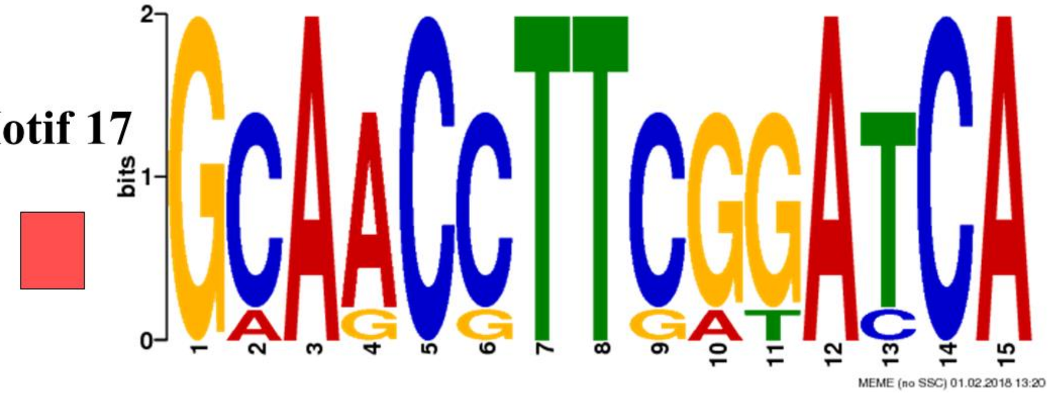

Gene Name	Sequences
PKS1 *Metarhizium anisopliae*	GCAACCTTCGGATCA
PKS1 *Metarhizium brunneum*	GCAGCCTTCGGATCA
PKS1 *Metarhizium robertsii*	GCAACCTTCGGATCA
PKS1 *Metarhizium guizhouense*	GCAACCTTCGGATCA
PKS1 *Metarhizium majus*	GCAACCTTCGGATCA
PKS1 *Metarhizium album*	GAAACCTTGGTACCA
PKS2 *Metarhizium acridum*	GCAACGTTCAGATCA

Motif 18

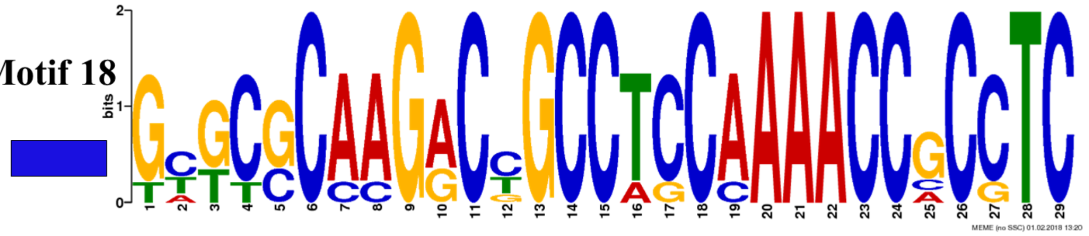

Gene Name	Sequences
PKS1 *Metarhizium album*	TCTTCCCCGACGGCCAGCCAAACCCCGTC
PKS2 *Metarhizium guizhouense*	GTGCGCAAGACTGCCTCCAAAACCGCCTC
PKS2 *Metarhizium majus*	GTGCGCAAGACTGCCTCCAAAACCGCCTC
PKS2 *Metarhizium brunneum*	GAGCCCAAGACCGCCTCCAAAACCACCTC
PKS2 *Metarhizium robertsii*	GCGCGCAAGGCCGCCTCCAAAACCGCCTC
PKS2 *Metarhizium anisopliae*	GCTCGCAAGGCCGCCTCCAAAACCGCCTC

Motif 19

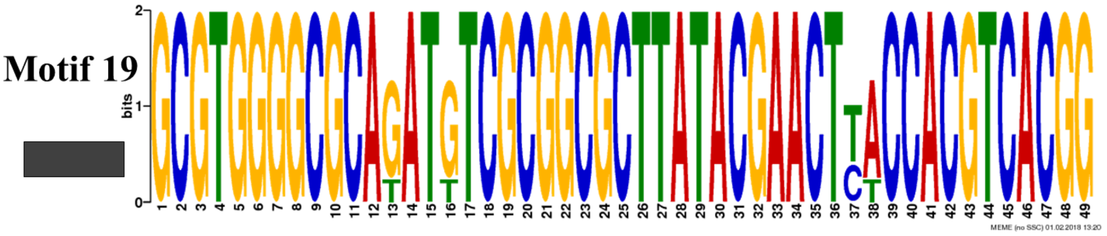

Gene Name	Sequences
PKS2 *Metarhizium guizhouense*	GCGTGGGGCGCAGATGTCGCGGCGCTTATACGAACTTACCACGTCACGG
PKS2 *Metarhizium majus*	GCGTGGGGCGCATATGTCGCGGCGCTTATACGAACTTACCACGTCACGG
PKS2 *Metarhizium brunneum*	GCGTGGGGCGCAGATTTCGCGGCGCTTATACGAACTTTCCACGTCACGG
PKS2 *Metarhizium robertsii*	GCGTGGGGCGCAGATGTCGCGGCGCTTATACGAACTCACCACGTCACGG
PKS2 *Metarhizium anisopliae*	GCGTGGGGCGCAGATGTCGCGGCGCTTATACGAACTCACCACGTCACGG

Motif 20

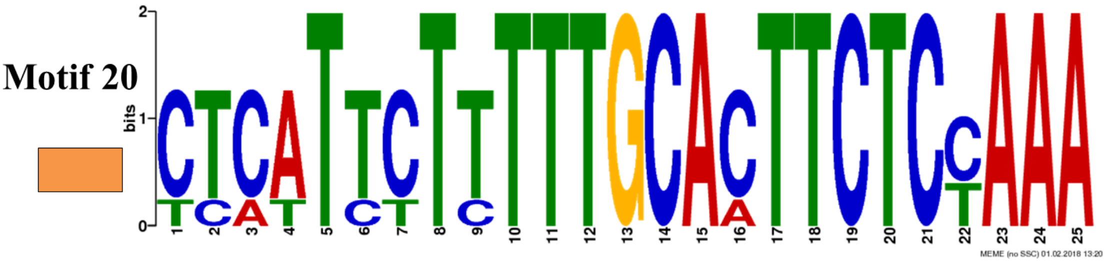

Gene Name	Sequences
PKS2 *Metarhizium guizhouense*	CTCATTCTTTTTGCACTTCTCCAAA
PKS2 *Metarhizium majus*	TCATTCTTCTTTGCACTTCTCCAAA
PKS2 *Metarhizium brunneum*	CTCATTCTTTTTGCACTTCTCTAAA
PKS2 *Metarhizium robertsii*	CTCATTCTTTTTGCACTTCTCTAAA
PKS2 *Metarhizium anisopliae*	CTCATTCTTTTTGCAATTCTCCAAA

Part 2: Promoter sequences of *Pks1* and *Pks2* genes

>MAA_PKS1 [Metarhizium robertsii ARSEF 23]

GCAAGTTTACTTTGGCAATTGTAAAGCACATTTCGTAGCAAATAGAGTTTGAACGTGGTGTCAAGATACCTC
CTATCCAACTTGCCACGGTGTGCTTGCTTGTTGCGTGGTTGCCTGTAGTCTTTGGAATGTACAAGGGCGCTA
AAGTTCGCGTTGGACAAGGGAAACTTTGATCTAGCCAACTACAGCAAACCGGAATCACCCTTAGCATGTCTT
GCGAATTTTAGGCTATTGTCCATCGATTCGTGATTCTGCCCTTGTTGTCTTGGCGATCGTGCCGTGATCTTGGA
TCCATTCTACGTGACAAGTAACTTCTGCTATCTACTAAGCACAAGCCCCGACAGCGTTGACGCCAAACCTAGA
CCGACGTTCCAGGCCGTTATTCTCGAACTTGTGACCTAACGTCTTGTAGTAATAAAGTTTCAAACACTCGGAT
CTTACTATTCCCTGGCCATGACATGGTATGCGTGTGACCTCATGATTATTAACTGTGACCACTTGGTAAGCAAT
CTCATTGCTCAATGTACTTAAGTCAAACCCGAGTAAAACAAATAAAAACGCTGTGAATTCAATGTCTGTGCTC
AAGCATCTCTACAGACCTAATGATCTTGTGTCTGTTGAACCGGGTGCAACCTTCGGATCAAC

>MAN_PKS1 [Metarhizium anisopliae ARSEF 549]

GCAAGTTTACTTCGATAATTGTAAAGCACATTTCGTAGCAAATAGAGTTTAAACGTGCTGTAAAGATACGTCC
TGTCCAACCTGCCACGGTGTGCTTGCTTGTTTCGTGGTTGCCTGTAGTCTTTGGAATGTACAAGGGCGCTAA
AGTTCGCGTTGGACAAGGGAAACTTTGATCTAGCCAACTACAGCAAACCGGTATCACCCTTAGTATGTCTCG
CGAATGTTAGGCTATTGTCCATCGGTTCGTGATTCTGCCCTTGTTATCTTGGCGATCGTGCCGTGAACTTGGAT
CCATTCTACGTGACAAGTAACTTCTGCCATCTACTAAGCACAAGCCCCGACAGCGTTGACGCCAAACCTAGA
CCGACGTTCCAGGCCGTTATTCTCGAACTTGTGACCTAACGTCTTGTAGTAATAAAGTTTCAAACACTCGGAT
CTTACTATTCCCTGGCCATGACATGGTATGCGTGTGACCTCATGATTATTAACCGTGACCACTTGGTAAGCAAT
CTCATTGCTCAATGTACTTAAGTCAAACCCGAGTAAAACAAATAAAAACGCTGTGAATTCAATGTCTGTGCTC
AAGCATCTCTACAGACCTAATGATCTTGTGTCTGTTGAACCGGGTGCAACCTTCGGATCAAC

>MBR_PKS1 [Metarhizium brunneum ARSEF 3297]

GCAAGTTTACTTTGGTAATTGTAAAGCACATTTCGTAGCAAATAGAGTTTAAACGTGGTGTCAAGATACGTCC
TATCCAACCTGCCACGGTGTGCTTGCTTGTTTCGTGGTTGCCTGTAGTCTTTGGAATGTACAAGGGCGCTAA
AGTTCGCGTTGGACAAGGGAAACTTTGATCTAGCCAACTACAGCAAACCGGAATCACCCTTAGTATGTCTTG
CGAATTTTAGGCTATTGTCCATCGATTCGTGATTCTGCCCTTGTTGTCTTGGCGATCGTGCCGTGAACTTGGAT
CCATTCTACGTGACAAGTAACTTCTGCTATCTACTAAGCACAAGCCCCGACAGCGTTGACGCCAAACCTAGA
CCGACGTTCCAGGCCGTTATTCTCGAACTTGTGACCTAACGTCTTGTAGTAATAAAGTTTCAAACACTCGGAT
CTTACTATTCCCTGGCCATGACATGGTATGCGTGTGACCTCATGATTATTAACCGTGACCACTTGGTAAGCAAT
CTCATTGCTCAATGTACTTAAGCCAAACCCGAGTAAAACAAATAAAAACGCTGTGAATTCAATGTCTGTGCTC
AAGCATCTCTACAGACCTAATGATCTTGTGTCTGTTGAACCGGGTGCAGCCTTCGGATCAAC

>MGU_PKS1 [Metarhizium guizhouense ARSEF 977]

CAAGTTTACTTTGCTAATTGCACAACACATCTCGTAGCAAATAGAGTTTAAACGTGGTGTCAAGATACTTCCTA
TCCAACTTGCCACGGTGTGCTTACTTATTTCATGGTTGCCTGTAGTCTTTGGAATGTAAAAGGGCGCTAAACT
TCGCGTTGGATAAAGGGAAACTTTGATCTAGCCAACTACAGAAAACCGAAATCACCCCTAGTATGTCTTGCG
AATTTTAGGCTATTGTCAATCGATTCGCAATTCTGCCCTTGTTGTCTTAGCGATCGTGCCGTGAATTTCGATCC
ATTCTACATGACAAGTAACTTCTGCATCTACTAAGCACATGCCCCGACAGCGTTGACGCCAAACCTAGACCGA
CGTTCCAGGCCATTATTCTCGAACGTGTGACCTAACGTCTTCTAGTAATAAAGTTTCAAATACTCGGATCTTAC
TATTCCCTGGCCCTGAAATGGTATGCGTGTGACCTCATGATTATTAACTGCCGGCCAGAGGGTAAGCAATCTC

ATGGCTCAATGTACTTAAGTCAAACCCGAGCAAAACAAATAAAAAAGCTGTTAATTCAATGTCTGTGCTCAAA
CATCTTTACAGACCTAATGATCTTGTGTCTGTTGAACCGGGTGCAACCTTCGGATCAAC

>MAJ_PKS1 [Metarhizium majus ARSEF 297]
AGAGCACTCAAATCATAGCGTGGCAATAAAGCTTCCAGCAAGTTTACTTTGGTAATTGCAACACACATCTCGT
AGCAAATAGAGTTTTAAACATATTTCATGGTTGCCTGTAGTCTTTGGAATGTAAAAGGGCGCTAAAGTTCGC
GTTGGATAAAGGGAAACTTTGATCTAGCCAACTACAGAAAACCGAAATCACCCCCTAGTATGTCTTGCGAAT
TTTACGCTATTGTCAATCGATTCGCAATTCTGCCCTTGTTGTCTTGGCGATCGTGCCGTGAATTTCGATCCATT
CTACGTGACAAGTAACTTCTGCATCTACCAAGCACATGCCCCGACAGCGTTGACGCCAAACCTAGACCGACG
TTCCAGGCCATTATTCTCGAACGTGTGACCTAACGTCTTCCAGTAATAAAGTTTCAAATACTCGGATCTTACTA
TTCCCTGGCCCTGAAATGGTATGCGTGTGACCTCATGATTATTAACTGCCACCAGAAGGGTAAGCAATCTCAT
GGCTCAATCAATGTACTTAAGTCAAACCCGAGCAAAACAAATAAAAAAAGCTGTTAATTCAATGCCTGTGCT
CAAACATCTTTACGGACCCAATGATCTTGTGTCTGTTGAACCGGGTGCAACCTTCGGATCAAC

>MAC_PKS1 [Metarhizium acridum CQMa 102]
CAAAACAAATCACATCTTGAAGCAAACAGAGTTTGCATGTGCTGTTAAGCTACATCGTATCCAACTTGTAACG
GTGTGCTTACTTATGTCGTGGCTGCCTGCAGTCTTTGGAATGTAGGACTGCGCTAAACTTTGCGTCAGATAAC
CGAAACTTTGATCTAGCCAACTACAGAAAACCGGAATCAACGTTAGTATATCTTGCAGATCTTAGACCATTCT
GAATTGCGCTATTTTGCACTTTGTTGTCTGAGCGATCCTGCTGTGAATTTCGATCCATTTGACTGTTTTTGACA
TGACATGTAACTTCTGCATCCACCAACAGCGTTAACCCAAAGTTAGACCGACGTTCCAGGCCATTATTCTCGA
ACGTGTAACCTGCCGTCTTCTGATAATAAAGTTTCGAACGCTCGGATCTTGCTACTCCCTGGCCCTGGAATGG
TATGTGTGTGACCTCATGATTATTAACTACGGCCACTTTTTAAGCAATCTCATCGCTGGAAGCGGATTTTGTAA
TCTTTCTGCAGTACTTAAGTCAACTCTAAGGAAAACAAATAACAAAACTATTAAATCAATATCTGTGCTCTAAT
ATCTTCAAGGACCTAATGATCTTGAGTCCGTTGGACCGGACGCAACGTTCAGATCAAC

>MAM_PKS1 [Metarhizium album ARSEF 1941]
ACTTGCCGGTCGGCATGCTTACATATGTCTCAGTTGCCTGCAGTCTTTGGAATGCAAAACTGTGGCTTTTAAG
GTTCACGTGGCCTGCTGTCTTTGGAATGCACAACCGCGGTTGTTAAAGTTGACCTTGAATGGGCGAAACTTT
GAATAGATGGAGCCAAGAAGGAAAAAATCCCCGAACACTCCACGGCACGCCGTGTACATTCCGGGCAAGA
AAAGTCCATTCGCCATGGTGCGGCTCCTGACTAGGCGGTCATCCCATGGACTTGGACCACTCTGCGTGAAAT
ATACAACTTCTGTTTCTACCGGGGACACGTCCCGACATCGTTAAGCTAAAAGCTAGAAACCGTTCTAGACCA
CTCTTCCCCGACGGCCAGCCAAACCCCGTCTGGTACCAAGGTTTCAAACGCTTCGCTTTCACTACTGGCTTG
TCCCAAAATGGCATGCGGGTGACCTCATGACTATCTGACCATGGACAAAGTAATCTCGCATTATATAGTTTGTT
CCTAGTACTTAAGTGAAAAGTAAAGCAAGAAAAGTCGAATTGCGGCGTGTTGAATACATATCTTTGATATCCT
CTAGGGCCCAACTACCTCGCGTCTGTCACATTGTTGGCTTCGAGTCTCGACGCCTGCCTGCATCAAC

>MAA_PKS2 [Metarhizium robertsii ARSEF 23]
TGTTGATATTTATCAAGGGAGCTGCACGTACGGTGCTTAGACCTACTTTCCTTGCAAGTTCAGATTTAGTGCG
TGGGGCGCAGATGTCGCGGCGCTTATACGAACTCACCACGTCACGGCGCTTCGTATACAAACAACGCGACA
GCAAAGTTTCAGGTCGGCGCTGAAAACGGCTGCCATGCCCACATCCAGGACGAGCAGACTGCCTACATAAT
CAGTTTCGCCTCACCGTGTCAGACCTGTCGTGGACTAACCTGATCCAACCGCCTCCTCCGTAGGTCGTGCTC
GCGTTGGCAAGGCTGAGCAACATGCGCGCAAGGCCGCCTCCAAAACCGCCTCCTGCACTGGTTTCACGTCC
GGTGAGCATACGAATGAACATTGAAGGCAAAGGACCCAGTGTTGCGGTGTGCCGGCCGCTAGAGTTGCCG
TCATAGGCGTGCTTAGCTATAGTCGTAGAAGAGAATCGACAGGGTTGGCTCCGTCTTCTATATGCTGTCACGG

CCGGATGGGGCGGTGGCCGTTTGACACACAGAATACGACAAGTGCTGAGGAGATGACGACTATAACATGC
AACTTTTGCCTTGATGGTTGGTGTGAATAGGCCATCTTTCCTCATTCTTTTTGCACTTCTCTAAACCTTGCGAC

>MAN_PKS2 [Metarhizium anisopliae ARSEF 549]
TTTTGTTGATATTTATCAAGGGAGCTGCACGTACGGTGCTTAGACCTACTTTCCTTGCAAGTTCAGATTTAGT
GCGTGGGGCGCAGATGTCGCGGCGCTTATACGAACTCACCACGTCACGGCGCTTCGTATACAAAACAACGC
GACAGCAAAGTTTCAGGTCGGCGCTGAAAACGGCTGCCATGCCCACATCCAGGACGAGCAGACTGCCTAC
ATAATCAGTTTCGCTTCACCGTGTCAGACCTGTCGTGGGCTAACCTGATCCAACCGCCTCCTCCGTAGGTCGT
GCTCGCGTTGGCAAGGCTGAGCAACATGCTCGCAAGGCCGCCTCCAAAACCGCCTCCTGCACTGGTTTCAC
GTCCGGTGAGCATACGAATGAACATTGAAGGCAAAGGACCCAGTGTTGCGGTGTACCGGCCACTAGAGTTG
CCGTCATAGGCGTGCTTAGCTATAGACGTAGAATCGACAGGGTTGGCTCCGTCTTCTATGAGGCGTCACGGC
CAGATGGGGCGGTGGCCGTTTGACACGCAGAATACGACAAGTGCTGAGGAGATGACGACTATAACATGCA
ACTTTTGCCTTGATGGTTGGTGTGAATAGGCCATCTTTCCTCATTCTTTTTGCAATTCTCCAAAACCTTGCGAC

>MBR_PKS2 [Metarhizium brunneum ARSEF 3297]
TTTTGTTGATATTTATCAAGGGAGCTGCACGTACGGTGCTTAGACCTACTTACCTTGCAAGTTCGGATCTAGT
GCGTGGGGCGCAGATTTCGCGGCGCTTATACGAACTTTCCACGTCACGGTGCTTCGTATACAAACAACGCG
ACAGCAAAGTTTCAGGTCGGCGCAGAAAACGGCTGCCATGCCCGCATCCACGACGAGCAGACTGCCTACAT
AATCAGTTTCGCCTCACCGTGTCAGACCTGTCGTGGACTAACCTGATCCAACCGCCTCCTCCGTTGGTTGGTA
GTGCTCCCGTTGGCAAGGCTGAGCAACATGAGCCCAAGACCGCCTCCAAAACCACCTCCTGCACTGGTTTC
ACGCCCGGTGAGCATACGACTGAAGGCAAAGGACCCAGTGTTGCGGTGTGCCGGCCGCTAGAGTTGCCGT
CATAGGCGTGCTTAGCTATAGTCGTAGAAGAGAATCGACAGGGTTGGCTCCGTCTGGTATACACCGTCACGG
CCGGATGGGGCGCTGGCCGTTTGACACGCAGAATACGACAAGTGCTGACGAGATGACGACTATAACATGCA
ACTTTTGCCTTGATGGTTGGTGTGAATAGGCCATCTTTCCTCATTCTTTTTGCACTTCTCTAAACCTTGCGAC

>MGU_PKS2 [Metarhizium guizhouense ARSEF 977]
GACTTGGCTTTTGTTGATATTTATCAAGGGAGCTGCACGTACGGTGCTTAGACCTACTTACCTTGCAAGTTCA
GATTTAGTGCGTGGGGCGCAGATGTCGCGGCGCTTATACGAACTTACCACGTCACGGTGCTTCGTATACAAA
CAACGCGACAGCAAAGTTTCAGGTCGGCGCAGAAAACGGCTGCCATGCCCGCATCCAGGACGAGCAGACT
GCCTACATAATCAGTTTCGCTTCACCGTGTCAGACCTGTCGTGGACTAACCTGATCCAACCGCCTCCTCCGTT
GGTCGTGCCCACGTTGGCAAGGCTGAGCAACATGTGCGCAAGACTGCCTCCAAAACCGCCTCCTGCACTGG
TTTCACGCCCGGTGAGCATACAACTGAAGGCAAAGGACCCAGTGTTGCGGTGTACCGGCCGCTAGAGTTGC
CGTCACAGGCGTGCTTAGCTATAGACGTAGAATCGACAGGGTTGGCTCCGTCTTCTATACACCGTCACGGCC
AGATGGGGCGGTGGCCTTTTGACACGCAGCATACGACAAGTGCTGAGGAGATGACGACTATAACATGCAAC
TTTTGCCTTGATGGTTGGTGTGAATAGGCCATCTTTCCTCATTCTTTTTGCACTTCTCCAAAACCTCGCGAC

>MAJ_PKS2 [Metarhizium majus ARSEF 297]
GGCTTTTGTTGATATTTATCAGGGGCGCTGCACGCACGGTGCTTAGACCTACTTACCTTGCAAGTTCAGATTT
AGTGCGTGGGGCGCATATGTCGCGGCGCTTATACGAACTTACCACGTCACGGTGCTTCGTATACAAACAAGG
CGACAGCAAAGTTTCAGGTCGGCGCAGAAAACGGCTGCCATTCCCGCATCCAGGGCGAGCAGACTGCCTA
CATAATCAGTTTCGCTTCAGCGTGTCAGACCTGTCGTGGACTAACCCTGATCCAACCGCCTCCTCCGTTGGTC
GTGCTCGCGTTGGCAAGGCTGAGCAACACGTGCGCAAGACTGCCTCCAAAACCGCCTCCTGCACTGGTTTC
ATGCCCGGTGAGCATACAACTGGAGGCAAAGGACCCAGTATTGCGGTGTACCGGCCGCTAGAGTTGCCGTC
ACAGGCGTGCTTAGCTATAGACGTAGAATCGGCAGGGTTGGCTCCGTCTTCTATACACCGTCACGGCCAGAT

GGGGCGGTGGCCTTTGACACGCTGGCAGCAGACGACAGGTGCTGAGGAGATGACGACTATAACATGCAAC
TTTTGCCTTGATGGTTGGTGTGAATAGGCCATCTTTCCTCATTCTTCTTTGCACTTCTCCAAAACCTCGCGAC

>MAC_PKS2 [Metarhizium acridum CQMa 102]
CGGTTCATCATTTGGCCTGTTTCATCTAGTAGGTGTCGTGAAATCAAGTCCTTCCCAGAGACGGCTGTACTAA
CAAGCTCAGCAAATATGGCAACGCGGGATGCCGCTACCCCAATGGGCAAGATATCCATGTGGCGGGAATGT
GCGTGGGCTCGTTGGCAGCAGCAGCAGTCAGTTGCTCCCGCTCCATAGGAGATCTAATAATAACTGGCATCG
TTGCCATCCGGGCCGCCTTGAGAGTAGGCCAGCGGGCCCATCAGGCGGCCCTGCTCATCAGCAATAGAGCA
GTACCTCCCACACATTGGTCGTACGCCGTTTCAACCGAGTCACTGCCGCTGGACTTGATAACCGATGCACTT
GCAAAGTTTGCAGAAAACATGGTATAGTGTGGTGGTCAACAGCAAGAACGGAGAACAATTCGTTAACAGA
CTGTGACAGGATACCTCTCCTCTAAGTAATCCCTATATCAGCGCTATCGGTTTAGATTCAGTCACTATCAGCGG
ACCTCCAAGCCAACTGCATCAGTTTTGGCAAGAAAACACACCTTCACACAAACCAATCTCTATCCCCATCTGG
GCTCCATATCATGGACCTCACATTTTTGGGGATATTGATATCGAAACCATTATAAAGAGTCTACATCCA
